# Predictors of cigarette smoking and physical inactivity among teachers during the SARS-CoV-2 pandemic in Germany: a cross-sectional analysis of a nationwide online survey

**DOI:** 10.3389/fpubh.2025.1458314

**Published:** 2025-04-28

**Authors:** Viktoria Eggert, Theresa Dicks, Kristin Kalo, Till Beutel, Carolina Zähme, Stephan Letzel, Clemens Koestner, Pavel Dietz

**Affiliations:** ^1^Institute of Occupational, Social and Environmental Medicine, University Medical Center of the Johannes Gutenberg University of Mainz, Mainz, Germany; ^2^Institute for Teachers' Health, University Medical Center of the Johannes Gutenberg University of Mainz, Mainz, Germany

**Keywords:** SARS-CoV-2, COVID-19, teachers, physical inactivity, cigarette smoking

## Abstract

**Background:**

The SARS-CoV-2 pandemic significantly impacted professional and private lives, which influenced social and health-related behavior. Schools in particular were greatly affected as restrictions made teaching more challenging, leading to new stresses and additional workloads. Prior to the pandemic, teachers were already facing many physical and psychological stressors that were exacerbated by the pandemic. This may have resulted in a deterioration in the teachers' health behaviors. Therefore, the aim of this study was to examine the prevalence of cigarette smoking and physical activity among German teachers during the SARS-CoV-2 pandemic, to assess possible changes considering cigarette smoking and physical activity habits during the pandemic compared to the pre-pandemic period, and to identify predictors of teachers' cigarette smoking and physical inactivity during the pandemic.

**Methods:**

In March 2021, a nationwide online survey was conducted among teachers in Germany. A total of 31,089 participants entered the analysis. Data on cigarette smoking and physical activity as well as sociodemographic, workplace-related, psychological, SARS-CoV-2-related, and health-related items were collected using established instruments and, if necessary, self-developed items. Two binary logistic regressions with stepwise inclusion of six different variable groups were performed to predict cigarette smoking and physical inactivity.

**Results:**

Among all surveyed teachers, 13.9% reported smoking cigarettes, and 76.6% did not meet the physical activity recommendations. The regression analyses revealed 16 significant predictors of cigarette smoking and six significant predictors of physical inactivity.

**Conclusions:**

The predictors revealed in the present study can help target interventions for teachers who are at higher risk for unhealthy behaviors during the SARS-CoV-2 pandemic and potential future pandemics. In particular, the alarming finding that more than three-quarters of teachers were physically inactive during the pandemic should place special emphasis on improving physical activity.

## 1 Introduction

Cigarette smoking and physical inactivity are two of the major risk factors for noncommunicable diseases (NCDs), which are the leading cause of death globally (1) and account for nearly 90% of all deaths in high-income Western countries, such as Germany, according to data from the European Environment Agency (2). Every year, more than 8.7 million people worldwide die due to tobacco use (3) and 3.2 million deaths can be attributed to physical inactivity (4).

Cigarette smoking is associated with a higher risk for coronary heart disease, arteriosclerosis, pulmonary diseases like chronic obstructive pulmonary disease (COPD), several types of cancer, and many other diseases (5–8). Even second-hand tobacco smoke can increase the risk of developing several diseases, such as ischemic heart disease, stroke, asthma, and lung cancer (5). Regarding the new severe acute respiratory syndrome coronavirus type 2 (SARS-CoV-2) and the associated new coronavirus disease 2019 (COVID-19), smoking is also a risk factor for the severe progression of the disease (9) and increases the risk of dying due to the disease (10).

Physical inactivity is associated with an increased risk for coronary heart disease, hypertension, type 2 diabetes, and different cancers as well as dementia, stroke, and depression (11). Being physically inactive for many weeks, months, or even years is also associated with increased systemic inflammation (12). Furthermore, physical inactivity can increase the risk of hospitalization, admission to the intensive care unit, and even death for patients with COVID-19 (13).

The National Center for Health Statistics (14) defines current smoking as “an adult who has smoked 100 cigarettes in his or her lifetime and who currently smokes cigarettes. Beginning in 1991, this group was divided into ‘everyday' smokers or ‘somedays' smokers”. According to the Global Burden of Disease Study of 2021, 1.14 billion people worldwide were designated as current smokers in 2019. The age-standardized prevalence of current smoking tobacco use among persons aged 15 years and older was 32.7% among men and 6.6% among women. The prevalence exceeded 20% among men in 151 countries and among women in 42 countries (15). Although the prevalence of cigarette smoking has declined since 1990, population growth has led to a significant increase in the total number of smokers (15).

In Germany, the difference between men and women in terms of their smoking behavior is much smaller. The prevalence of current cigarette smoking is 29.9% for men and 23.0% for women (15). Among men, smoking prevalence decreases from the age of 45, while among women, a significant decrease is not observed until the age of 65 (16). Among both women and men, cigarette smoking is significantly less prevalent in higher education groups than in lower education groups, and this clear difference is evident for almost all age groups (16).

Regarding physical activity, the World Health Organization (WHO) recommends adults aged 18–64 years at least 150–300 min per week of moderate-intensity aerobic physical activity or at least 75–150 min of vigorous-intensity aerobic physical activity as well as additional muscle-strengthening activities at moderate or greater intensity (17). Physical inactivity is therefore defined as not meeting these recommendations (18). Nevertheless, Guthold et al. (19) stated that in 2016, more than a quarter of adults around the world were insufficiently physically active. With the exception of East and Southeast Asia, women are less physically active than men in all regions of the world. In high-income countries, 36.8% of the population showed insufficient physical activity, compared to 16.2% in low-income countries, and between 2001 and 2016, there were only marginal and insignificant decreases in the levels of insufficient physical activity (19). This means that there has been no progress in reducing global levels to meet the 2025 global physical activity target of a 10% relative reduction in the prevalence of insufficient physical activity by 2025 (20) and even less progress to meet the extended global physical activity target of a 15% relative reduction by 2030 (21).

In Germany, only 44.8% of women and 51.2% of men met the recommendations for physical activity. While the percentage of adults aged 18 to 29 is the highest, it decreases among the age groups and is lowest among adults aged 65 or above (22). Both women and men are more likely to achieve the recommendations if they are in the higher education group than if they are in the medium and low education group (22).

The SARS-CoV-2 pandemic has led to the implementation of restrictions on social life and contacts, which resulted in major changes in many aspects of everyday life (23). These restrictions included advisories to stay at home, bans on gatherings, and closures of “nonessential” businesses like gyms, hair salons, or restaurants (24).

Studies from different countries show that health-related factors and behaviors changed due to the restrictions. For instance, studies from Italy, Belgium, and the United States reported an increase in tobacco consumption attributed to increased stress, more time at home, and boredom (25–27). Conversely, a recent systematic review by Almeda and Gómez-Gómez (28) revealed a decline in cigarette smoking during the SARS-CoV-2 pandemic in the majority of the included cohort studies which might be related to the reduction in social gatherings among other things.

The restrictions concerning the use of public spaces in particular have changed opportunities to be physically active. For example, sports classes did not take place, and recreational opportunities were limited. Furthermore, distances that were actively traveled before the pandemic were now reduced due to home office, among other things (29). In a systematic review from Stockwell et al. (30), the majority of the included studies reported a decrease in physical activity during the pandemic. In addition, studies from France, Sweden, and the United Kingdom showed that moderate-to-vigorous physical activity levels decreased, whereas there was a slight increase in strength training during the pandemic (31–33).

The SARS-CoV-2 pandemic also led to wide-ranging changes in many companies and facilities. Measures such as home office and reduced working hours have been introduced to reduce contacts within companies (34). Likewise, schools in Germany had to be closed in March 2020, as in many countries around the world, to prevent further spread of the disease. As a result, teaching formats had to be changed to online instruction, which presented challenges for teachers and students (35). For many teachers, this was the first time they had to use digital tools for teaching. Even after the schools reopened, there were major changes in the work of the teachers. The measures to contain the spread of the virus required adjustments and made teachers' work more difficult (36).

Teachers are a large occupational group, as there are currently approximately 800,000 teachers in Germany (37). They have important educational and pedagogical tasks and contribute to the stability of society as well as to the further development of future generations (38). Therefore, from a public health point of view and overall social perspective, it is important to focus on teachers' health. Prior to the pandemic, teachers were already burdened by time pressure, many working hours, loud noises, a high number of students per class as well as high levels of tension, limited recovery during the day, and mixing of work and leisure time (38–40). These aspects can have an impact on the health of teachers if they are not mastered. With the SARS-CoV-2 pandemic, new stressors appeared for teachers. Previous studies have already shown that the pandemic had a negative influence on teachers' mental health, as they observed higher emotional burdens as well as higher depression and anxiety symptoms during the SARS-CoV-2 pandemic compared with the general population (41, 42).

Further research has shown that work-related stress as well as job strain can have an impact on risky health behaviors like smoking, alcohol consumption, dietary fat intake, and physical inactivity (43–46). Griep et al. (47) reported associations between high job strain and physical inactivity in women and men. Heikkilä et al. (48) showed that individuals exposed to occupational stress were less likely to maintain a healthy lifestyle, and Kouvonen et al. (49) demonstrated that employees with high work stress were more likely to be smokers. They also found out that high work stress was associated with higher smoking intensity (49).

Given that the SARS-CoV-2 pandemic may have created many changes as well as new challenges and burdens for teachers, a lack of knowledge exists with regard to the impact of this new situation on teachers' health risk behaviors, such as cigarette smoking and physical inactivity. Therefore, the present study aimed to address this knowledge gap by (i) assessing the prevalence of cigarette smoking and physical activity among teachers in Germany during the SARS-CoV-2 pandemic, (ii) determining changes in cigarette smoking and physical activity among teachers during the SARS-CoV-2 pandemic, and (iii) identifying potential predictors of teachers' cigarette smoking and physical inactivity.

## 2 Materials and methods

### 2.1 Study design and survey procedure

Between March 1 and March 31, 2021, a cross-sectional nationwide online survey was conducted among German teachers of all school types. The survey was designed using LimeSurvey software (LimeSurvey GmbH, Hamburg, Germany). Participants were recruited through cooperation with the Ministry of Education in Rhineland-Palatinate, the Education and Science Workers' Union, the German Teachers Association, and the Project “Monitor Lehrerbildung”. An unconditional non-monetary incentive was offered (EUR 2,000 donation to the German Children's Fund) to foster willingness to participate, and one reminder email was sent during the survey period.

Participation in the survey was voluntary and anonymous, and informed consent was obtained digitally in advance. Ethical approval to conduct this study was given by the ethical committee of the Medical Association of Rhineland-Palatinate (2020-15531).

### 2.2 Measures

The online survey consisted of approximately 350 items, which were mostly taken from validated questionnaires and supplemented with self-constructed or adapted items, if necessary. The questions covered a wide range of topics, including sociodemographic and workplace-related questions, SARS-CoV-2-specific strains and challenges in schools for teachers, the implementation, communication, and compliance with hygienic guidelines or plans (both general and school-based), the impact of school operations during the SARS-CoV-2 pandemic on teachers, and examples of proven measures. A list of all surveyed items can be found in Supplementary Table S1.

To ensure correct understanding and associations of the items as well as linguistic and grammatical quality, the survey was pretested in several steps by experts from the Institute for Teachers' Health and the Institute of Occupational, Social and Environmental Medicine of the University of Mainz as well as a small sample of teachers.

#### 2.2.1 Dependent variables

The two dependent variables were cigarette smoking and physical inactivity. The question asked for cigarette smoking was “Do you currently smoke?” with four response options: “Yes, daily”, “Yes, occasionally”, “No, not anymore” and “No, I have never smoked”. To classify the participants into physically inactive or active, the construct of physical activity was assessed using the following question: “On how many days in the past week were you physically active for 30 min or more so that your breathing rate was elevated? This may include sports, exercise, and brisk walking or cycling for recreation or to get to places, but not housework or physical activities that are part of your job.” Response options were “0 days”, “1 day”, “2 days”, “3 days”, “4 days”, “5 days”, “6 days”, and “7 days” (50).

In addition, two questions were asked to assess possible changes in the habits of cigarette smoking and physical activity during the SARS-CoV-2 pandemic compared to the pre-pandemic period. The questions asked were, “How would you describe this aspect compared to before the COVID-19 pandemic?” Response options for cigarette smoking were: “Currently much more than before the COVID-19 pandemic”, “Currently somewhat more than before the COVID-19 pandemic”, “About the same as before the COVID-19 pandemic”, “Currently somewhat less than before the COVID-19 pandemic”, and “Currently much less than before the COVID-19 pandemic”. Response options for physical activity were “Currently much more frequent than before the COVID-19 pandemic”, “Currently somewhat more frequent than before the COVID-19 pandemic”, “About as frequent as before the COVID-19 pandemic”, “Currently somewhat less frequent than before the COVID-19 pandemic”, and “Currently much less frequent than before the COVID-19 pandemic”.

#### 2.2.2 Independent variables

To predict cigarette smoking and physical inactivity, 49 independent variables with regard to the research questions were selected from the questionnaire. Physical activity and cigarette smoking were also used as independent variables to test as possible predictors of the other dependent variable. A detailed list of the variables, including the specific questions, scales, and the respective references, is given in Table 1. The 49 independent variables were classified into the following six different groups: (1) sociodemographic variables (four variables, e.g., gender and age); (2) work-related variables—organizational or general conditions (12 variables, e.g., school type, professional group, and work schedule); (3) work-related variables—work-related impacts and attitudes (11 variables, e.g., global job satisfaction, workload, and work-privacy conflict); (4) psychological variables (five variables, e.g., emotional exhaustion, loneliness, and depression); (5) SARS-CoV-2-related variables (13 variables, e.g., private burdens, helplessness, and health concerns); and (6) health-related variables (four variables, e.g., somatic complaints and substance use).

**Table 1:** List of all dependent and independent variables differentiated in the six variable groups.

**Dimensions**	**Variable**	**Scale/reference**	**Items**	**“Example of questions”; answering options**
Dependent variables	Physical activity	Milton et al. (50)	1	“On how many days in the past week were you physically active for 30 min or more so that your breathing rate was elevated? This may include sports, exercise, and brisk walking or cycling for recreation or to get to places, but not housework or physical activities that are part of your job.”; 0 days/1 day/2 days/3 days/4 days/5 days/6 days/7 days
	Cigarette smoking	Self-constructed item	1	“Do you currently smoke?”; yes, daily/yes, occasionally/no, not anymore/no, I have never smoked
Sociodemographic variables	Gender	Self-constructed item	1	“Gender”; female/male/diverse
	Age	Self-constructed item	1	“Age (in years)”
	People in the household	Self-constructed item	1	“Number of people in your household (Note: Please count yourself in this)”
	Number of minor children in the household	Self-constructed item	1	“Of which minor children living in own household”
Work-related variables (organizational/general conditions)	School type	Self-constructed item	1	“What type of school do you work at?”; primary school/secondary general school/secondary school/comprehensive school/academic secondary school/special needs school/vocational school/other
	Professional Group	Self-constructed item	1	“Which professional group do you belong to?”; teacher/teaching aid/candidate/other
	School Management Team	Self-constructed item	1	“Are you part of the school management team?”; yes/no
	Employment	Self-constructed item	1	“Employment”; civil servant/employed, permanent contract/employed, temporary contract/other
	Work schedule	Self-constructed item	1	“Work schedule”; full-time/part-time
	Subjects taught	Self-constructed item	1	“What subjects do you teach?”
	Number of classes taught	Self-constructed item	1	“How many classes do you teach?”
	Grade levels taught (lowest & highest)	Self-constructed item	2	“What grade levels do you currently teach?”; from…until…
	Federal State	Self-constructed item	1	“In which state is your office located?”
	Multiple departments	Self-constructed item	1	“Do you work at more than one office (e.g., two schools or school and seminary)?”; yes/no
	Close student contact	Self-constructed item	1	“Are there care situations that involve close contact with students (e.g., all-day school, working groups, liaison teacher)?”; yes/no
	Care of students	Self-constructed item	1	“Are you involved in the care of students (e.g., at a special education school)?”; yes/no
Work-related variables (work-related impacts and attitudes)	Global job satisfaction	Self-constructed item	1	“How satisfied are you with your professional situation overall?”; not at all/little/somewhat/very much/extremely
	Time requirements	COPSOQ 2020 (84)	1	“How often does it happen that you do not have enough time to complete all your tasks?”; never/almost never/rarely/sometimes/often/always
	Predictability of work	COPSOQ 2020 (84)	1	“Are you informed well in advance of changes in your workplace, such as important decisions, changes, or plans for the future?”; to a very low degree/to a low degree/in part/to a high degree/to a very high degree
	Information needed for work	COPSOQ 2020 (84)	1	“Are you getting all the information you need to do your job well?”; to a very low degree/to a low degree/in part/to a high degree/to a very high degree
	Influence on work	COPSOQ 2020 (84)	1	“Do you have much influence over decisions that affect your work?”; never/almost never/rarely/sometimes/often/always
	Influence on the amount of work	COPSOQ 2020 (84)	1	“Do you have any influence on the amount of work you are assigned?”; never/almost never/rarely/sometimes/often/always
	Work-related emotional demands	COPSOQ 2020 (84)	1	“Is your work emotionally demanding?”; to a very low degree/to a low degree/in part/to a high degree/to a very high degree
	Workability	Self-constructed item	1	“When you think about your state of health and your professional capacity, do you think you will be able to work until you reach retirement age?”; no way/rather no/unsure/rather yes/sure
	Workload	Self-constructed item	1	“Please check how much the following problem has burdened you during the last week. How much do you feel burdened by your professional situation?”; not at all/little/somewhat/very much/extremely
	Work-Privacy-Conflict	COPSOQ 2020 (84)	2	1. “My work takes up so much energy that it has a negative impact on my personal life.”; to a very low degree/to a low degree/in part/to a high degree/to a very high degree 2. “My work takes up so much time that it has a negative impact on my personal life.”; to a very low degree/to a low degree/in part/to a high degree/to a very high degree
	Meaningfulness of work	COPSOQ 2020 (84)	1	“Is your work meaningful?”; to a very low degree/to a low degree/in part/to a high degree/to a very high degree
Psychological variables	Emotional exhaustion	West et al. (97)	1	“How often do you feel burned out from your work?”; never/at least a few times a year/at least once a month/a few times a month/once a week/several times a week/daily/no answer
	Depersonalization	West et al. (97)	1	“How often do you feel that you have become more uncaring in your interactions with other people since you started this job?”; never/at least a few times a year/min. once a month/sometimes per month/once a week/multiple times a week/daily/no answer
	Loneliness	Beutel et al. (98)	1	“I am often alone, have few contacts”; no does not apply/yes applies and has not burdened me/yes applies and has burdened me little/yes applies and has burdened me moderately/yes applies and has burdened me a lot/no answer
	General anxiety	GAD2 (99)	2	“During the past 2 weeks, how often did you feel affected by the following complaints?” 1. Not being able to stop or control worries; not at all/on some days/on more than half the days/almost every day 2. nervousness, anxiety, or tension; not at all/on some days/on more than half the days/almost every day
	Depression	PHQ2 (99)	2	“During the past 2 weeks, how often did you feel affected by the following complaints?” 1. Little interest or pleasure in your activities; not at all/on some days/on more than half the days/almost every day 2. Dejection, melancholy, and hopelessness; not at all/on some days/on more than half the days/almost every day
SARS-CoV-2-related variables	Burden due to changes in school organizational processes	Self-constructed item	1	“Do you find this change stressful?”; not at all/to a very small extent/to a small extent/to some extent/to a great extent/to a very great extent
	Burden due to an increase in the amount of information for school-related matters (e.g., emails, messengers, notices, verbal)	Self-constructed item	1	“Do you find this change stressful?”; not at all/to a very small extent/to a small extent/to some extent/to a great extent/to a very great extent
	Burden due to an increase in the amount of work	Self-constructed item	1	“Do you find this change stressful?”; not at all/to a very small extent/to a small extent/to some extent/to a great extent/to a very great extent
	Burden due to problems with the implementation of the educational mission	Self-constructed item	1	“Do you find this change stressful?”; not at all/to a very small extent/to a small extent/to some extent/to a great extent/to a very great extent
	Burden of higher expectations of your students' guardians for work	Self-constructed item	1	“Do you find this change stressful?”; not at all/to a very small extent/to a small extent/to some extent/to a great extent/to a very great extent
	Burden of difficulty in achieving the intended learning goals with the students	Self-constructed item	1	“Do you find this change stressful?”; not at all/to a very small extent/to a small extent/to some extent/to a great extent/to a very great extent
	Private burdens	Self-constructed item	1	“Since the beginning of the COVID-19 pandemic, private stresses have increased overall in my life.”; do not agree at all/tend not to agree/partly/tend to agree/agree completely/no answer
	Household conflicts	Self-constructed item	1	“Since the onset of the COVID-19 pandemic, there has been increased conflict in my household.”; do not agree at all/tend not to agree/partly/tend to agree/agree completely/no answer
	Restrictions in leisure activities	Self-constructed item	1	“Since the onset of the COVID-19 pandemic, there have been restrictions in my leisure activities (e.g., sports, club activities, meetings with friends)”; do not agree at all/tend not to agree/partly/tend to agree/agree completely/no answer
	Helplessness	Self-constructed item	1	“I feel helpless in the face of the current COVID-19 pandemic”; do not agree at all/tend not to agree/partly/tend to agree/agree completely
	Uncertainty	Self-constructed item	1	“Not knowing how long the current COVID-19 pandemic will last worries me.”; do not agree at all/tend not to agree/partly/tend to agree/agree completely
	Health concerns	Self-constructed item	1	“The prospect of working at my school/service during the COVID-19 pandemic worries me in terms of my health.”; do not agree at all/tend not to agree/partly/tend to agree/agree completely
	Expected course of disease	Self-constructed item	1	“If you were to be diagnosed with COVID-19, how likely do you think it would develop into a severe disease?”; 0 (extremely unlikely)−100 (extremely likely)
Health-related variables	WHO-self rated general health	Cislaghi and Cislaghi (100)	1	“How would you describe your health in general?”; very poor/poor/not quite satisfactory/satisfactory/good/very good
	Somatic complaints in the last 4 weeks	PHQ15 (101)	15	“During the past 4 weeks, how much did you feel affected by the following complaints?” Abdominal pain/back pain/pain in arms, legs, or joints (knees, hips, etc.)/menstrual pain or other problems with menstruation/pain or problems with intercourse/headache/pain in sternum/dizziness/fainting spells/palpitations or rapid heartbeat/shortness of breath/constipation, nervous bowel, or diarrhea/nausea, gas, or indigestion/difficulty falling asleep or staying asleep through the night or increased sleep/fatigue or feeling like you have no energy; not impaired/slightly impaired/severely impaired.
	Substance Use	Self-constructed item	1	“I have been using more addictive substances (e.g., alcohol, tranquilizers) since the onset of the COVID-19 pandemic.”; do not agree at all/tend not to agree/partly/tend to agree/agree completely

### 2.3 Data analysis

Data cleaning was performed to exclude cases that had dropped out at the beginning of the survey or had only answered the sociodemographic questions but no further questions regarding the research questions. Furthermore, implausible values (e.g., stated age outside the working age range: below 18 years or above 67 years) were marked as missing. Duplicates were removed from the data set using a pseudonymization code.

Statistical analysis was performed using SPSS Statistics 27 (IBM, Armonk, NY). To demonstrate the sample characteristics, descriptive analyses were conducted. For continuous scaled variables, descriptive statistics are presented as means with standard deviations (SD), and non-continuous scaled variables are shown as numbers and percentages. For further analyses, certain variables were operationalized. Some categorical variables were converted into dummy variables. For the gender variable, the category “diverse” was excluded from further analyses because of the small number of cases compared to the other two categories (*n* = 139).

Pretests were performed for each of the 49 independent variables (84 after dummy-coding categorial variables) using Spearman's correlation for continuous variables (Supplementary Table S2) and Pearson's chi-square (χ^2^) test for categorical variables (Supplementary Tables S3, S4). Only variables that showed a significant association with cigarette smoking or physical activity (*p* ≤ 0.05) were included in further analyses.

To predict cigarette smoking and physical inactivity, two binary logistic regressions with stepwise inclusion of the six variable groups in each regression model were performed. Therefore, the dependent variables of cigarette smoking and physical inactivity were dichotomized. Cigarette smoking was dichotomized into “do not smoke” (= 0) and “smoke” (= 1), and physical inactivity was dichotomized into “physically active” (= 0) and “physically inactive” (= 1). The single item developed by Milton et al. was designed for the purpose of evaluating physical activity in relation to the national recommendation of a minimum of 30 min of physical activity on five or more days per week, with a single question (51). In accordance to this, the answers “5 days”, “6 days”, and “7 days” were defined as “physically active”, while all other answers to this variable were defined as “physically inactive”. This classification has been used in different studies before, including two recently published articles by Nicholls and Watson (52) and Staley et al. (53). To check for multicollinearity, a collinearity matrix and the variance inflation factor (VIF) were determined. Furthermore, the minimum sample size of the regression model was calculated using the formula *n* = 100 + 50i, where *i* is the number of independent variables proposed by Bujang et al. (54). This resulted in a minimum sample size of *n* = 4,300, calculated for the 84 independent variables used in this study.

## 3 Results

A total of 39,359 teachers from all 16 federal states in Germany participated in the study. After data cleaning, a sample of *N* = 31,089 was used for further analysis. Overall, 77.5% of the participants reported being female, 22.0% were male, and 0.4% were diverse. The age of the participants ranged from 18 to 67 years, and the average age was 45.8 (± 10.5) years. Of the participants, 94.8% were teachers, 2.0% were teaching aids, and 3.2% stated they were candidates for teaching. Furthermore, most of the participants worked at primary schools (32.2%), followed by academic secondary schools (19.5%), and comprehensive schools (14.4%). Detailed sample characteristics are presented in Table 2.

**Table 2:** Sample characteristics.

**Variable**	***n* (%)^a, b^**	**Range, *M* (*SD*)**
**Gender**	**31,089 (100.0)**	
Female	24,099 (77.5)	
Male	6,851 (22.0)	
Diverse	139 (0.4)	
**Age**	**31,089 (100.0)**	18–67, 45.8 (10.5)
18–30 years	2,473 (8.0)	
31–43 years	10,957 (35.2)	
44–55 years	10,799 (34.7)	
56–67 years	6,860 (22.1)	
**Persons in household** ^**c**^	**30,706 (100.0)**	1–9, 2.7 (1.2)
1 person	4,540 (14.8)	
2 people	11,373 (37.0)	
3 people	5,538 (18.0)	
4 people	6,844 (22.3)	
5+ people	2,411 (7.9)	
**Number of minor children in household**	**29,096 (100.0)**	0–9, 1.75 (1.0)
0 children	16,468 (56.6)	
1 child	5,082 (17.5)	
2 children	5,839 (20.1)	
3+ children	1,707 (5.9)	
**School type** ^**d**^	**27,960 (100.0)**	
Primary school	9,030 (32.3)	
Secondary general school	539 (1.9)	
Secondary school	2,162 (7.7)	
Comprehensive school	4,016 (14.4)	
Academic secondary school	5,451 (19.5)	
Special needs school	2,696 (9.6)	
Vocational school	2,699 (9.7)	
Other	1,367 (4.9)	
**Professional group**	**30,313 (100.0)**	
Teacher	28,748 (94.8)	
Teaching aid	605 (2.0)	
Candidate	960 (3.2)	
**Being part of the school management**	**30,981 (100.0)**	
Yes	3,290 (10.6)	
No	27,691 (89.4)	
**Employment**	**31,019 (100.0)**	
Civil servant	23,192 (74.8)	
Employed, permanent contract	6,606 (21.3)	
Employed, fixed-term contract	940 (3.0)	
Other	281 (0.9)	
**Work schedule**	**30,959 (100.0)**	
Full-time	18,662 (60.3)	
Part-time	12,297 (39.7)	
**Subjects taught**	**29,733 (100.0)**	
German	15,395 (18.3)	
Foreign languages	9,324 (11.1)	
STEM	15,850 (18.9)	
Social sciences	14,967 (17.8)	
Musical subjects	10,230 (12.2)	
Religion, philosophy, ethics	5,914 (7.0)	
Physical education	5,779 (6.9)	
Other	6,445 (7.7)	
**Number of classes taught**	**30,337 (100.0)**	0–39, 4.7 (3.2)
**Lowest grade level taught**	**29,804 (100.0)**	1–13, 5.0 (3.0)
**Highest grade level taught**	**28,252 (100.0)**	1–13, 8.2 (3.7)
**Federal state**	**30,792 (100.0)**	
Baden-Württemberg	5,935 (19.3)	
Bavaria	913 (3.0)	
Berlin	2,496 (8.1)	
Brandenburg	903 (2.9)	
Bremen	431 (1.4)	
Hamburg	1,374 (4.5)	
Hesse	2,994 (9.7)	
Mecklenburg-Western Pomerania	488 (1.6)	
Lower Saxony	3,430 (11.1)	
North Rhine-Westphalia	5,520 (17.9)	
Rhineland-Palatinate	2,839 (9.2)	
Saarland	216 (0.7)	
Saxony	985 (3.2)	
Saxony-Anhalt	370 (1.2)	
Schleswig-Holstein	1,328 (4.3)	
Thuringia	570 (1.9)	
**Working at multiple departments**	**30,836 (100.0)**	
Yes	3,936 (12.8)	
No	26,900 (87.2)	
**Situations with close student contact**	**30,836 (100.0)**	
Yes	20,125 (65.3)	
No	10,711 (34.7)	
**Involvement in the care of students**	**30,851 (100.0)**	
Yes	2,213 (7.2)	
No	28,638 (92.8)	

^*a*^The values in bold indicate the total number of participants who answered the respective questions. ^*b*^The number of participants *(n)* may differ between items, because responding to the questions was voluntary, and therefore, not all participants answered all the questions. ^*c*^Including the participants. ^*d*^Multiple answers were possible for the question on the type of school. Only those participants who gave exactly one answer are shown in this evaluation. M, means; SD, standard deviation; STEM, science, technology, engineering, and mathematics.

### 3.1 Prevalence of cigarette smoking and physical activity

**Figure 1:**
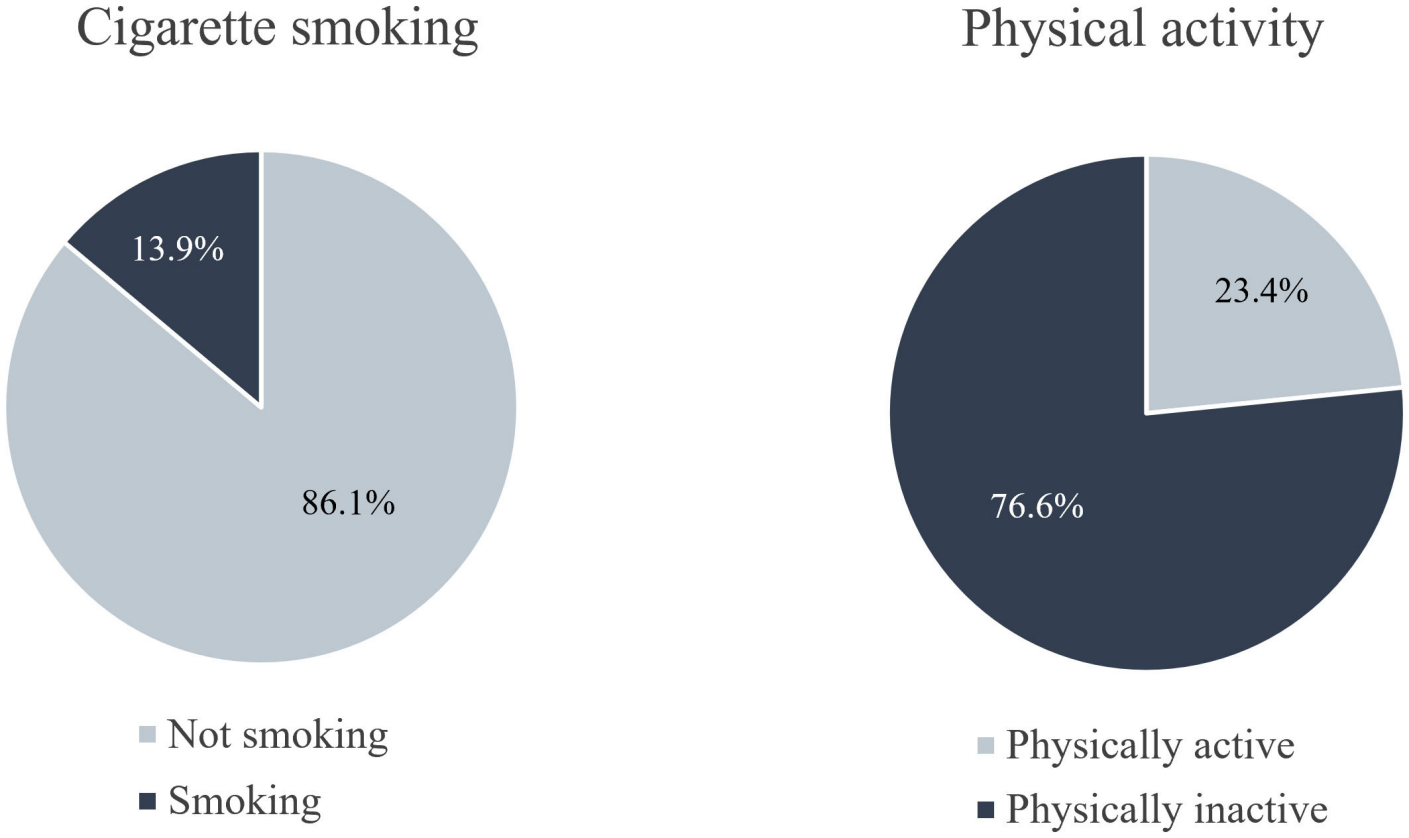
Distribution of cigarette smoking and physical activity among teachers in Germany during the SARS-CoV-2 pandemic in March 2021.

Of the 31,089 participants, 21,305 answered the question on cigarette smoking, and 21,273 responded to the question on physical activity. Of the 21,273 teachers who filled out the question on physical activity, more than three-quarters (76.6%) were physically inactive. In addition, almost one-seventh (13.9%) of teachers reported smoking (Figure 1).

**Figure 2:**
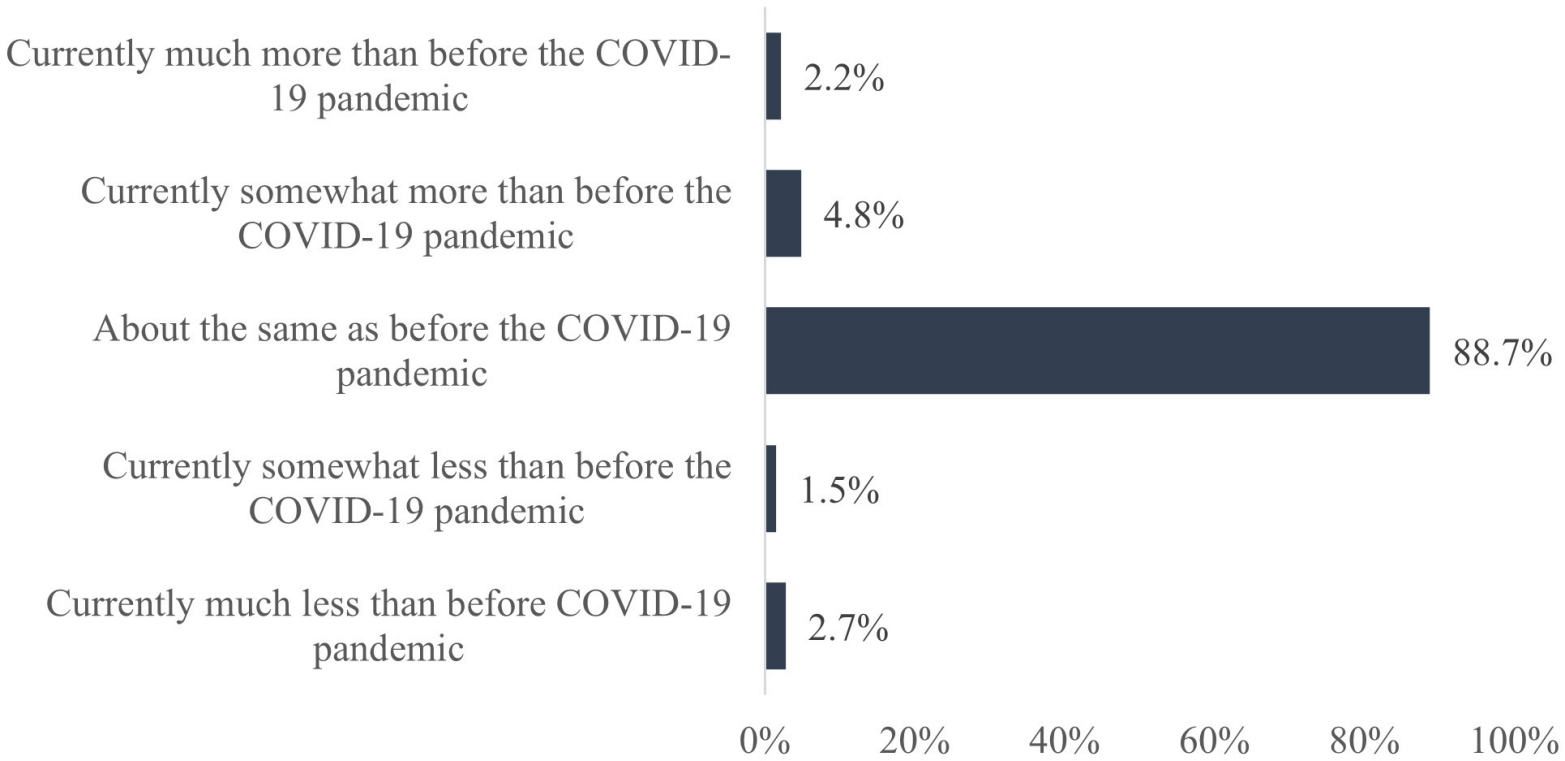
Change in cigarette smoking due to the SARS-CoV-2 pandemic compared to the pre-pandemic period.

**Figure 3:**
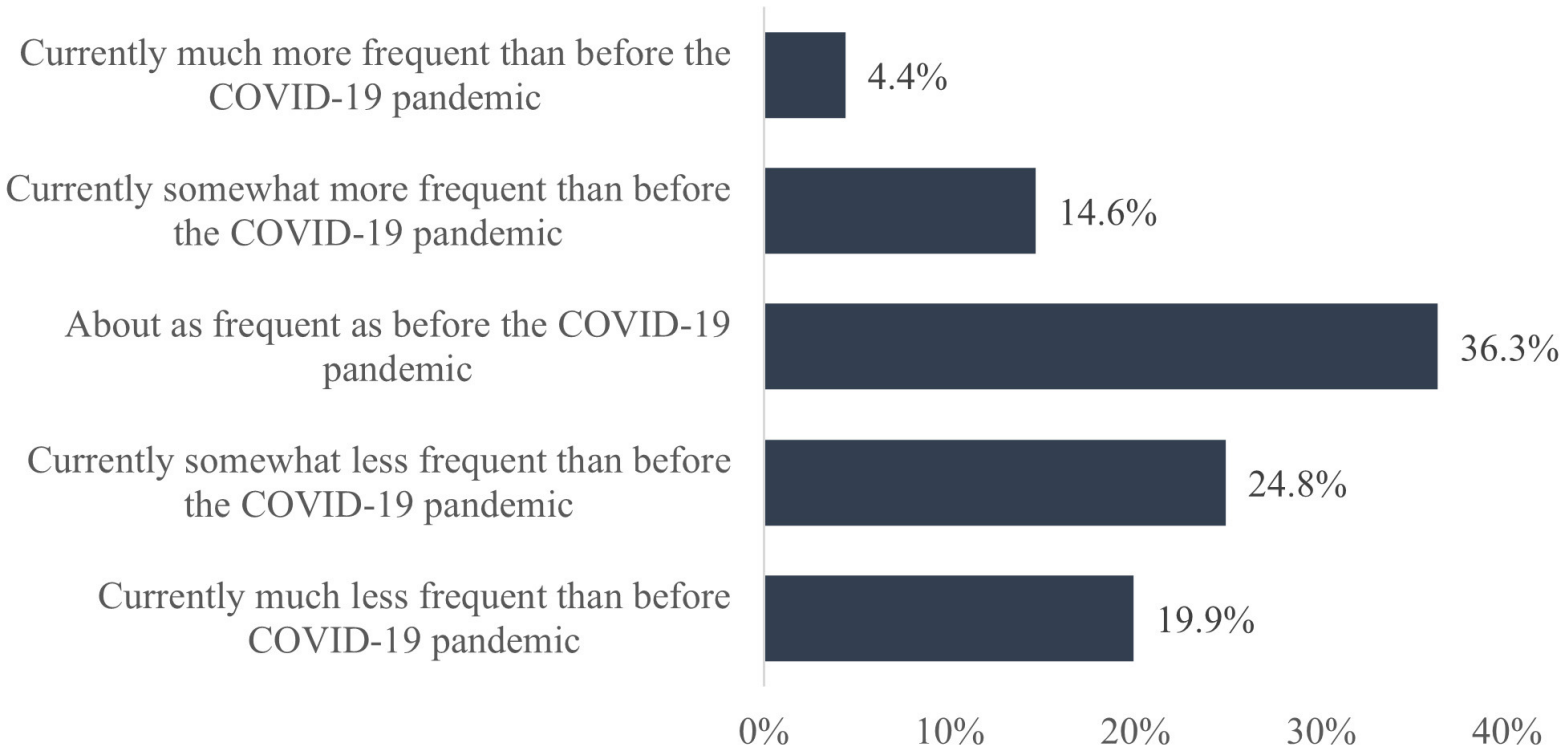
Change in physical activity due to the SARS-CoV-2 pandemic compared to the pre-pandemic period.

Additionally, 19,318 teachers answered the question regarding changes in their cigarette smoking habits during the SARS-CoV-2 pandemic compared to the pre-pandemic period. The results show that the majority of teachers (88.7%) smoked about as many cigarettes as before the pandemic (Figure 2). Regarding physical activity, this question was answered by 21,136 teachers, and in this case, the results showed that 36.3% were equally and 44.7% less frequently physically active compared to before the pandemic (Figure 3).

### 3.2 Predictors of cigarette smoking

The pretests revealed 56 variables that were significantly associated with cigarette smoking (Supplementary Tables S2, S3). The binary logistic regression included *n* = 9,414 cases, exceeding the previously calculated minimum sample size for the regression (see Section 2.3). The overall model of the stepwise binary logistic regression was statistically significant, χ^2^ (23) = 615.506, *p* < 0.001. Furthermore, no collinearity of the chosen variables was given (the average VIF was 1.7, the lowest was 1.0, and the highest was 7.3). After stepwise inclusion of each variable group, the explained variance (Nagelkerke *R*^2^) increased with each included group. Starting at 1.9% in the first step, the explained variance increased to 11.6% after the last (sixth) step (please see Supplementary Table S5 for all steps individually). The overall percentage of accuracy in classification was 86.5%.

In the final step of the regression, 16 independent variables were identified that significantly predicted cigarette smoking. These can be found in Table 3. According to the six variable groups, the distribution of the significant variables was as follows^1^: (1) “gender—female”, and “persons in household”, (2) “school type” (primary school, academic secondary school), “work schedule – full-time”, “subject taught” (German, STEM, social sciences), “federal state – Berlin”, (3) “global job satisfaction”, “influence on work”, (5) “burden due to problems with the implementation of the educational mission”, “health concerns”, “expected course of disease”, (6) “physical activity”, and “substance use”.^2^

**Table 3:** Significant predictors of cigarette smoking.

	**OR (95% CI)**	***p***
**Sociodemographic variables**		
Gender—female	0.692 (0.595–0.804)	<0.001
Persons in household	0.809 (0.764–0.856)	<0.001
**Work-related variables— organizational/general conditions**		
Primary school	0.736 (0.624–0.868)	<0.001
Academic secondary school	0.757 (0.640–0.895)	0.001
Work schedule—full-time	1.153 (1.001–1.328)	0.048
German	1.248 (1.088–1.430)	0.002
STEM	0.861 (0.756–0.981)	0.025
Social sciences	1.212 (1.065–1.379)	0.004
Federal state—Berlin	1.990 (1.633–2.425)	<0.001
**Work-related variables—work related impacts and attitudes**		
Global job satisfaction	0.906 (0.832–0.986)	0.022
Influence on work	1.102 (1.030–1.178)	0.005
**SARS-CoV-2-related variables**		
Burden due to problems with the implementation of the educational mission	1.100 (1.013–1.193)	0.023
Health concerns	0.920 (0.868–0.975)	0.005
Expected course of disease	1.152 (1.086–1.222)	<0.001
**Health-related variables**		
Physical activity	0.945 (0.915–0.975)	<0.001
Substance use	1.586 (1.507–1.669)	<0.001

Significant predictors of cigarette smoking in a binary logistic regression analysis with stepwise inclusion of the six independent variable groups: sociodemographic, work-related (organizational or general conditions), work-related (work-related impacts and attitudes), psychological, SARS-CoV-2-related, and health-related variables. Observed cases: *n* = 9,414; R^2^ = 0.116; χ^2^ (23) = 615.506; *p* < 0.001; please find the results of the binary logistic regression model for all included (significant and non-significant) variables in Supplementary Table S5. OR, odds ratio; CI, confidence interval.

Female teachers had a lower likelihood of cigarette smoking than male teachers. The more people living in the household, the less likely teachers were to smoke cigarettes. Working in a primary or academic secondary school was also negatively related to cigarette smoking, meaning that those teachers had a lower likelihood of smoking. Teachers who worked full-time were more likely to smoke cigarettes than teachers who worked part-time. While there was a negative association between teaching STEM subjects and smoking cigarettes, teachers who taught German or social sciences were more likely to smoke. Furthermore, teachers who worked in Berlin were more likely to smoke cigarettes. In addition, the more satisfied teachers were with their jobs, the less likely they were to smoke. On the other hand, the more influence they had on decisions about their work, the more likely they were to smoke. Experiencing stress due to increasing problems in implementing the educational mission was positively related to smoking cigarettes, and the higher the probability of severe COVID-19 infection was assessed, the more likely teachers were to smoke. On the other hand, teachers who were more concerned about their health were less likely to smoke. Among the health-related variables, physical activity was negatively related to smoking, whereas substance use was related to a higher likelihood of smoking.

### 3.3 Predictors of physical inactivity

After the pretests, 48 variables were significantly associated with physical inactivity (Supplementary Tables S2, S4). The previously calculated minimum sample size of 4,300 cases was exceeded, given that 6,260 cases were included in the binary logistic regression. The overall model of the stepwise binary logistic regression was statistically significant, χ^2^ (13) = 159.724, *p* < 0.001. Testing for multicollinearity also revealed no collinearity of the variables (the average VIF was 1.7, the lowest was 1.0, and the highest was 7.3). The explained variance of the model after the sixth step was 3.9%, and it was correctly classified as 78.2%. The explained variance increased with each step, starting with 0.4% in the first step (all steps individually can be found in Supplementary Table S6).

The last step of the binary logistic regression revealed six significant predictors for physical inactivity^3^: (1) “age”, (2) “school type—primary school”, and “subjects taught—physical education”, (3) “work-related emotional demands”, (5) “restrictions in leisure activities”, and (6) “self-rated general health” (see text footnote ^2^). Table 4 shows the odds ratios of the significant predictors of the binary logistic regression after the sixth step.

**Table 4:** Significant predictors of physical inactivity.

	**OR (95% CI)**	***p***
**Sociodemographic variables**		
Age	0.989 (0.983–0.995)	<0.001
**Work-related variables—organizational/general conditions**		
Primary school	1.203 (1.051–1.377)	0.007
Physical education	0.678 (0.583–0.789)	<0.001
**Work-related variables—work related impacts and attitudes**		
Work-related emotional demands	0.820 (0.750–0.897)	<0.001
**SARS-CoV-2-related variables**		
Restrictions in leisure activities	1.216 (1.103–1.340)	<0.001
**Health-related variables**		
Self-rated general health	0.837 (0.788–0.889)	<0.001

Significant predictors of physical inactivity in a binary logistic regression analysis with stepwise inclusion of the six independent variable groups: sociodemographic, work-related (organizational or general conditions), work-related (work-related impacts and attitudes), psychological, SARS-CoV-2-related, and health-related variables. Observed cases: *n* = 6,260; R^2^ = 0.039; χ^2^ (13) = 159.724; *p* < 0.001; please find the results of the binary logistic regression model for all included (significant and non-significant) variables in Supplementary Table S6. OR, odds ratio; CI, confidence interval.

The older the teachers were, the more likely they were to be physically active. Teachers who worked in primary schools had a higher likelihood of being physically inactive, whereas teachers who taught physical education were more likely to be physically active. Work-related emotional demands were negatively related to physical inactivity, meaning that the higher the emotional demands, the lower the likelihood of not being physically active for at least 30 min on at least five days a week. Teachers who reported having had limitations in leisure activities had a higher likelihood of being physically inactive, and the better the teachers' self-rated general health was, the less likely they were to be physically inactive.

## 4 Discussion

The present study examined cigarette smoking and physical inactivity of teachers during the SARS-CoV-2 pandemic. In addition, relevant predictors for cigarette smoking and physical inactivity among German teachers were identified. The results are discussed by attempting to compare the present findings with other studies that examined teachers. When this was not possible, other populations, such as the general population or other occupational groups, were considered. The findings of the binary logistic regressions may provide a basis for targeted health-related interventions by pointing out highly burdened groups.

### 4.1 Prevalence of cigarette smoking and physical activity

During the pandemic, the overall prevalence of cigarette smoking among German teachers was 13.9%. Compared to the general population, the prevalence of cigarette smoking among teachers was much lower than that of the general population in Germany [23% (55)]. This is in line with the results of studies prior to the pandemic. For example, Scheuch et al. (38) stated that teachers had better health behaviors than the general population. Regarding smoking, they showed that there were half as many smokers among teachers as among the general population (38). Similar results were found by Seibt et al. (56) who showed that teachers smoked less compared to a regional employment sample. There are several possible explanations for why teachers smoke less than people in other professions. One reason could be that teachers are a professional group with a high level of education. It is generally known that people with a university degree tend to smoke less than people with lower school qualifications (57). Another reason could be the role model function of teachers. Many teachers are aware of the possible influence of their personal behavior on children's behavior (58).

Furthermore, the results of the present study show that the majority of teachers (88.7%) smoked about as much as before the pandemic. This can also be seen in a study by Klosterhalfen et al. (59), who analyzed smoking behavior among the general population in Germany. According to their study, most of the participants smoked about as much as before the pandemic.

Moreover, 76.6% of the teachers in the present study were classified as physically inactive, as they reported being active for a minimum of 30 min on less than 5 days per week during the pandemic. This prevalence is much higher than that found in a study by Wilke et al. (60) who performed a multinational survey in 14 countries among the general population. They reported that 37.5% of the general population did not meet the physical activity recommendations during the pandemic, and that the number of people meeting the recommendations decreased by almost 20% during that time. This is in contrast to data from pre-pandemic studies reporting a smaller prevalence of physical inactivity among teachers compared to the general population (38).

As a second result, considering the physical activity status of teachers, the present study revealed that 44.7% of teachers surveyed were less active than before the pandemic. This is in line with the results of other studies. In a systematic review by Stockwell et al. (30), for example, the authors also stated that physical activity decreased during the pandemic, and Wilke et al. (60) reported that overall self-reported physical activity declined by 41%.

### 4.2 Predictors of cigarette smoking and physical inactivity

With regard to the second research question of identifying predictors for cigarette smoking and physical inactivity among teachers during the pandemic, the present results show that out of the six variable groups, 16 independent variables were significantly related to cigarette smoking. Among the sociodemographic variables, “gender” and “number of people living in the household” were significant variables. Similarly to these results, other authors have described that female teachers, as well as women in general, smoke less than male teachers and men in general (15, 38). The present results further show that the more people living in a household, the less likely teachers are to smoke cigarettes. One possible explanation for this could be that teachers with more people living in their households might have children and smoke less to protect their children's health.

For work-related variables regarding organizational or general conditions, the school types “primary school” and “academic secondary school” were negatively related to cigarette smoking. To date, there is limited research concerning smoking behavior and different school types. Nevertheless, an older study from Bewley et al. (61) confirms these results, stating that academic secondary school teachers smoke less than teachers of other school types. A recent study by Temam et al. (62), on the other hand, showed that there were no significant differences between primary and secondary school teachers. Nonetheless, it is difficult to compare the results of the present study to those from other studies, since other studies did not subdivide school types as much. Moreover, the differences in educational systems between countries, especially with regard to school types, make the studies hardly comparable.

Furthermore, the variables “work schedule” and “subject taught” were significantly related to cigarette smoking. Teachers who worked full-time had a higher likelihood of smoking cigarettes. Similar results can be found, for example, in a study by Angrave et al. (63) who reported that long working hours are likelier to result in smoking. In addition, they provided some evidence that long working hours might lead to increased cigarette consumption among smokers. A possible explanation for this could be that long working hours may act as a stressor for workers since they tend to feel less happy and relaxed as well as more anxious when at work (64, 65). People who smoke may experience smoking as stress relieving (66). Currently, no research is available regarding the associations between the subjects taught by teachers and cigarette smoking. Furthermore, it is not apparent from the present data set why such a relationship might exist. It would be interesting to investigate this as well as the influence of the individual school types on the smoking behavior of teachers in further studies.

Another significant variable of the second variable group to predict cigarette smoking was the federal state of Berlin. Being a teacher in Berlin was positively related to smoking cigarettes. In a health report by Lampert et al. (67), the authors compared people's health behavior among the 16 federal states of Germany. Regarding smoking behavior, they showed that Berlin was among the states with the most people smoking. This might be an explanation for the results of the present study that teachers working in Berlin have a higher likelihood of cigarette smoking.

Among the variables of work-related impacts and attitudes, a small protective association regarding cigarette smoking could be detected for “global job satisfaction”. The higher the job satisfaction, the lower the likelihood of smoking. This is consistent with the results of previous studies (68, 69). On the other hand, the variable “influence on work” was significantly related to a higher likelihood of smoking. To date, there have been no studies on the influence on work-related decisions and the likelihood of smoking cigarettes. Nor do the present data explain this relationship. Therefore, it would be interesting to examine this question in future studies.

Interestingly, and also a little bit surprisingly, none of the psychological variables were significantly related to cigarette smoking. While other studies showed an association between cigarette smoking and psychological factors—especially for depression and anxiety (70, 71)—this does not seem to be the case for German teachers.

Among the SARS-CoV-2-related variables, three variables significantly predicted cigarette smoking: “burden due to problems with the implementation of the educational mission”, “health concerns”, and “expected course of disease”. Teachers who were concerned about their health due to working at the school during the pandemic were less likely to smoke, and teachers who reported a higher likelihood of severe COVID-19 illness were more likely to smoke. As reported by several authors, cigarette smoking increases the risk and severity of pulmonary infections due to upper respiratory tract damage and decreases lung immune function (72, 73). With regard to the new SARS-CoV-2 virus, smokers were also found to be at an increased risk of infection and more severe disease progression (9, 74). This might explain why teachers who were concerned about their health tried to stay healthy and minimize their risk of infection by not smoking, and it might also explain the association between a worse expected disease outcome and smoking.

Out of the last variable group (health-related variables), “physical activity” and “substance use” significantly predicted smoking. Teachers who were more active had a lower likelihood of smoking, and teachers who had a higher substance use since the beginning of the pandemic had a higher likelihood of smoking. According to many different authors, health risk behaviors are interrelated and often occur in combination with one another (75–78). In particular, a positive correlation has been reported, for example, between smoking and alcohol consumption (75, 77, 79). On the other hand, people who are physically active may be less prone to unhealthy habits, such as tobacco use. Johnson et al. (80) and Walker et al. (81) for example reported a negative association between physical activity and cigarette smoking.

The results of the second binary logistic regression revealed six variables that were significantly related to physical inactivity. Among the sociodemographic variables, only “age” was a significant predictor. As age increased, the likelihood of being physically inactive decreased. This is in contrast to the results of a study by Richter et al. (22) who showed that the percentage of German adults meeting the WHO recommendations of being physically active for at least 150 minutes per week decreased with age and was highest among the youngest age group and lowest among the oldest age group.

Out of the second variable group concerning work-related organizational or general conditions, the school type “primary school” and the subject “physical education” significantly predicted physical inactivity. Teachers who worked at a primary school had a higher likelihood of being physically inactive than teachers working at other school types. Seibt et al. (82) showed similar results among teachers in the federal state of Saxony, Germany. According to their analyses, teachers working in a primary school had the lowest prevalence of physical activity compared to the other school types prior to the pandemic. On the other hand, physical education teachers were more likely to be physically active. Similar results were reported in a study by Aydogmuş et al. (83) who analyzed the physical activity levels among physical education teachers in Turkey. Their results showed that physical education teachers continued to be active during the SARS-CoV-2 pandemic.

The third variable group, “work-related impacts and attitudes”, only revealed one variable (“work-related emotional demands”) significantly predicting physical inactivity. Higher emotional demands were related to a higher likelihood of being physically active. Work-related emotional demands are considered psychological stresses and strains at work and are one part of the Copenhagen Psychological Questionnaire (COPSOQ III; (84)). Studies investigating the influence of occupational stress on leisure-time physical activity have generally found that high occupational stress is related to lower levels of physical activity (85, 86). This is in contrast to our findings. However, these studies did not specifically examine the influence of emotional demands on physical activity.

Similar to the first binary logistic regression for cigarette smoking, this regression analysis did not reveal significant predictors of the fourth variable group (psychological variables) for physical inactivity among German teachers. In contrast, previous studies have reported associations between psychological variables such as loneliness, depression, or anxiety and physical inactivity (87–89). However, not all of these studies have examined these associations among teachers.

Among the variables in the fifth group, only the variable “restrictions in leisure activities” significantly predicted physical inactivity. Teachers who reported having had restrictions on their leisure activities were less likely to be physically active. Due to restrictions to mitigate the spread of the SARS-CoV-2 virus, gyms and sports clubs were closed or were only able to allow a very small number of people to be there at the same time (90). In 2021, there were an estimated 27 million members of sports clubs in Germany (91) and about 9.3 million members of fitness studios (92). The closure of these facilities deprived millions of Germans of the opportunity to engage in regular sports activities. Even though many fitness coaches and experts offered online training or exercise videos, only a small number of people used these opportunities during the pandemic (93).

Out of the last variable group concerning health-related variables, “self-rated general health” was a significant predictor of physical inactivity. This finding shows that the better the self-rated general health of teachers, the less likely they were to be physically inactive. It is well known that physical activity is beneficial to health and physical performance, and that physically active adults have better self-rated health than non-active individuals (94, 95). Conversely, a logical conclusion would be that individuals, or, in this case, teachers, who rate their health as good or very good are more likely to be physically active.

### 4.3 Practical implications

The results of the present study provide a good overview of the prevalence of two important health behaviors: cigarette smoking and physical activity. Given the alarming finding that more than three-quarters of teachers were classified physically inactive, special emphasis should be placed on improving physical activity, as it is a crucial factor in somatic and mental health. Especially in times of limited physical activity opportunities, such as during a pandemic with restrictions in daily life, alternative physical activity options should be made more attractive so that teachers can be active despite additional workloads. At-risk groups for increased smoking and physical inactivity should particularly be the focus of targeted interventions, taking into account the predictors demonstrated in the present study. Factors associated with stress for teachers, such as long working hours and low job satisfaction as well as health-related factors, should be considered and targeted for improvement, as they are related to higher smoking and poorer physical activity behaviors.

Given the paucity of studies on school-related factors affecting smoking and physical inactivity among teachers, further studies should focus on these topics to verify the present findings.

### 4.4 Limitations

The present study has limitations that need to be considered. The dependent variables were recorded with only one item each. This limits the interpretability of the results, as several questions could have been helpful, especially with regard to physical activity levels. Also, despite the fact that the single-item measure appears to be an indicator of the number of days with ≥30 min of physical activity, this question does not provide information about the total amount of time spent being physically active or the type of activity performed. Consequently, respondents who are physically active for longer periods of time on fewer days may be categorized as physically inactive with this question. Furthermore, the question on physical activity used in this study only recorded the activity behavior of the past week. Even though short recall periods seem to be more precise and decrease the magnitude of reporting error in physical activity estimates, they do not estimate habitual physical activity or long-term adherence which would need to be assessed using long-term recall periods (96). Therefore, with the results of the preset study, illnesses or unusually high levels of stress or workload during the surveyed period may allow misleading conclusions to be drawn about general activity behavior of teachers.

Another limitation of this study is the small *R*^2^, therefore, the results of the binary logistic regression must be interpreted with caution. It seems that the variables studied do not explain the variance with respect to the two dependent variables. For this reason, it would be advisable for future studies to investigate other school- or work-related factors that may explain the smoking and physical activity behaviors of teachers. On the other hand, the present study has a very large number of cases, so the results still have high significance.

In the case processing of the linear regression for cigarette smoking, which was computed only to check for multicollinearity, two variables were excluded by the statistical program (“primary school” and occupational group “teacher”). As it is not possible to gain insight into the processing of the data by the statistical program, it is a matter of speculation as to why they were excluded from the linear regression. However, since these variables are of particular content relevance, they were not excluded from the binary logistic regression, the main analysis of this study. Therefore, the results related to these two variables must be interpreted with caution, as they may be biased.

Furthermore, the cross-sectional design of this study limits the investigation to a specific timeframe of the pandemic. It is possible that a different time period of the pandemic would have shown different results. In particular, the fact that the study took place in the midst of the third wave of the pandemic, during which there was a large increase in the number of cases, could bias the results. It is possible that teachers were exposed to more stress and uncertainty during this time, making them less able to focus on their health.

## 5 Conclusion

Overall, this study showed that teachers had better smoking behavior during the SARS-CoV-2 pandemic compared to data from the general population. Nevertheless, factors like working full time, teaching German or social science, working in the state of Berlin, expecting a bad course of disease, or having increased substance use during the pandemic seem to amplify the likelihood of smoking cigarettes. On the other hand, the prevalence of teachers' physical inactivity was alarmingly high during the pandemic. Factors such as younger age, experiencing restrictions in leisure activities, and having bad self-rated health seem to increase the likelihood of being physically inactive. Efforts to reduce the identified prevalence should be made with these factors in mind.

The present results reflect the picture during a particular phase of the pandemic. However, what this means for teachers after the pandemic is currently unknown. It would be interesting to find out in further studies how teachers' smoking behavior and physical activity changed after the pandemic. It is also recommended that the factors found in this study be considered and improved where possible to facilitate better health behaviors in teachers in future situations, such as the SARS-CoV-2 pandemic. Teachers are an important occupational group that educates future generations. Especially in this group, health is essential. For this reason, a special focus should continue to be placed on their behaviors, such as smoking habits or physical activity, and these should be supported through interventions. A possible example of this could be fitness programs for teachers at schools, which could be implemented during breaks or after work.

Future studies should validate the findings of this study, especially considering school-specific variables. In addition, more variables should be examined to explain the associations with smoking and physical inactivity since only a fraction of the variance could be explained by the variables used in the regressions.

## Data Availability

The raw data supporting the conclusions of this article will be made available by the authors, without undue reservation.

## Footnotes

^1^The exact list of the six variable groups can be found in Section 2.2.2.

^2^There were no significant variables out of the fourth variable group.

^3^The numbers in brackets are again associated with the variable groups from Section 2.2.2.
