# Supplementary material for: Predictors of cigarette smoking and physical inactivity among teachers during the SARS-CoV-2 pandemic in Germany: a cross-sectional analysis of a nationwide online survey
Source: Front Public Health. 2025 Apr 28;13:1458314. doi: 10.3389/fpubh.2025.1458314 (PMC12066700; doi:10.3389/fpubh.2025.1458314)
Supplement: Supplementary file 1 [file Data_Sheet_1.pdf]

Supplementary Material

Table S1. List of all items included in the online-survey.

lfd.Nr. je Abschnitt	Item Kürzel	Übergeordnetes Thema / Konstrukt	Item / Fomulierung	Antwortskala / Abstufungen	Herkunft (Instrument)	Name der Skala	Originale Formulierung?	Skala vollständig?	Ausrichtung Item Zustimmung / hohe Ausprägung = positiv oder negativ?	Quelle Itemformulierung	Quelle Validität	Quelle Reliabilität
1. Soziodemografische Variablen & Angaben zum Arbeitsplatz												
Soziodemografie												
1	A1	Geschlecht	Geschlecht	/weiblich<br>/männlich<br>/divers	eigenes Item	-	-	-	-	-	-	-
2	A2	Alter	Alter (in Jahren)	[Freitext]	eigenes Item	-	-	-	-	-	-	-
3	A3	Personen in Haushalt	Anzahl der Personen in Ihrem Haushalt?	[Freitext]	eigenes Item	-	-	-	-	-	-	-
4	A4	Anzahl minderjährige Kinder im Haushalt	Davon minderjährige Kinder im eigenen Haushalt lebend	[Freitext]	eigenes Item	-	-	-	-	-	-	-
5	SW1	Schwangerschaftsstatus	*(Filterfrage, wenn A1 = weiblich ODER divers)*<br>Sind sie aktuelle schwanger?	/ja<br>/nein<br>/keine Antwort	eigenes Item							
Angaben zum Arbeitsplatz												
6	A5	Schulart	An welcher Schulart sind Sie tätig? (Mehrfachnennung möglich)	/Grundschule<br>/Hauptschule<br>/Realschule<br>/Realschule plus (Kombination aus Haupt- und Realschule)<br>/Integrierte Gesamtschule (Kombination aus Haupt- und Realschule sowie Gymnasium)<br>/Gymnasium<br>/Förderschule<br>/Berufsschule<br>/Andere Schulart [Freitext]	eigenes Item	-	-	-	-	-	-	-
7	A6	Berufsgruppe	Welcher Berufsgruppe gehören Sie an?	/Lehrkraft<br>/Pädagogische Fachkraft / Unterrichtshilfe / Assistent*in o.ä.<br>/Anwäter*in<br>/Sonstige	eigenes Item	-	-	-	-	-	-	-
10	A7	Schulleitungsteam	Sind Sie Teil des Schulleitungsteams?	/ja<br>/nein	eigenes Item		-	-	-			
8	A8	Beschäftigungsverhältnis	Beschäftigungsverhältnis	/verbeamtet<br>/beschäftigt, unbefristeter Vertrag<br>/beschäftigt, befristeter Vertrag<br>/Sonstiges	eigenes Item	-	-	-	-	-	-	-
9	A9	Vollzeit/Teilzeit	Arbeitszeitmodell	/Vollzeit<br>/Teilzeit	eigenes Item	-	-	-	-	-	-	-
11	A10	Unterrichtsfächer	Welche Fächer unterrichten Sie?	[Freitext]<br>[Freitext]<br>[Freitext]<br>[Freitext]<br>[Freitext]<br>[Freitext]	eigenes Item		-	-	-			
12	A11	Anzahl Klassen	Wie viele Klassen unterrichten Sie?	[Freitext]	eigenes Item	-	-	-	-	-	-	-
13	A12	Unterrichtete Klassenstufen	Welche Klassenstufen unterrichten Sie aktuell?	von [Freitext]<br>bis [Freitext]	eigenes Item	-	-	-	-	-	-	-
14	A13	Bundesland Dienstelle	In welchem Bundesland befindet sich Ihre Dienststelle?	/Baden-Württemberg<br>/Bayern<br>/Berlin<br>/Brandenburg<br>/Bremen<br>/Hamburg<br>/Hessen<br>/Meklenburg-Vorpommern<br>/Nordrhein-Westfalen<br>/Niedersachsen<br>/Rheinland-Pfalz<br>/Saarland<br>/Sachsen<br>/Sachsen-Anhalt<br>/Schleswig-Holstein<br>/Thüringen	eigenes Item	-	-	-	-	-	-	-

15	A14	Multiple Dienststellen	Arbeiten Sie an mehr als einer Dienststelle (z. B. zwei Schulen oder Schule und Studienseminar)?	/ja<br>/nein	eigenes Item	-	-	-	-	-	-	-
16	A15	Situationen mit engem Schülerkontakt	Gibt es Betreuungssituationen, die engen Schülerkontakt mit sich bringen (z. B. Ganztagsschule, AGs, Vertrauenslehrer*in)?	/ja<br>/nein			-	-	-	-	-	
17	A16	Pflege Schüler*innen	Sind Sie in die Pflege von Schüler*innen involviert (z.B. an einer Förderschule)?	/ja<br>/nein	eigenes Item	-	-	-	-	-	-	-

2. Identifizierung von SARS-CoV-2-spezifischen Belastungen und Herausforderungen

2a) Organisatorisch

Erleben Sie aufgrund der COVID-19-Pandemie Veränderungen in nachfolgend genannten Bereichen im Vergleich zur Zeit vor der COVID-19-Pandemie?

1	CAO1	Veränderung schulorganisatorischer Prozesse	Schulorganisatorische Prozesse im Allgemeinen	/Ja<br>/Nein	eigenes Item	-	-	-	-	-	-	-
2	CAO1a	Bewertung	(Filterfrage, falls „Ja") Empfinden Sie diese Veränderung als belastend?	/in sehr hohem Maße<br>/in hohem Maße<br>/zum Teil<br>/in geringem Maße<br>/in sehr geringem Maße<br>/gar nicht	eigenes Item	-	-	-	negativ	-	-	-
3	CAO2	Veränderung der Stundenplangestaltung	Stundenplangestaltung (z. B. Vorlaufzeit, Vollständigkeit oder Klarheit)	/Ja<br>/Nein	eigenes Item	-	-	-	-	-	-	-
4	CAO2a	Bewertung	(Filterfrage, falls „Ja") Empfinden Sie diese Veränderung als belastend?	/in sehr hohem Maße<br>/in hohem Maße<br>/zum Teil<br>/in geringem Maße<br>/in sehr geringem Maße<br>/gar nicht	eigenes Item	-	-	-	negativ	-	-	-
5	CAO3	Veränderung der Raumnutzung	Einschränkungen bei der Raumnutzung (z. B. Platzmangel)	/Ja<br>/Nein	eigenes Item	-	-	-	-	-	-	-
6	CAO3a	Bewertung	(Filterfrage, falls „Ja") Empfinden Sie diese Veränderung als belastend?	/in sehr hohem Maße<br>/in hohem Maße<br>/zum Teil<br>/in geringem Maße<br>/in sehr geringem Maße<br>/gar nicht	eigenes Item	-	-	-	negativ	-	-	-
7	CAO4	Veränderung der Pausengestaltung	Pausengestaltung (z. B. getrennte Kleingruppen, Veränderung der Anzahl an Pausenaufsichtspersonen)	/Ja<br>/Nein	eigenes Item	-	-	-	-	-	-	-
8	CAO4a	Bewertung	(Filterfrage, falls „Ja") Empfinden Sie diese Veränderung als belastend?	/in sehr hohem Maße<br>/in hohem Maße<br>/zum Teil<br>/in geringem Maße<br>/in sehr geringem Maße<br>/gar nicht	eigenes Item	-	-	-	negativ	-	-	-
9	CAO5	Veränderung des Austauschs mit Kolleg*innen	Möglichkeit zum Austausch mit Kolleg*innen	/Ja<br>/Nein	eigenes Item	-	-	-	-	-	-	-
10	CAO5a	Bewertung	(Filterfrage, falls „Ja") Empfinden Sie diese Veränderung als belastend?	/in sehr hohem Maße<br>/in hohem Maße<br>/zum Teil<br>/in geringem Maße<br>/in sehr geringem Maße<br>/gar nicht	eigenes Item	-	-	-	negativ	-	-	-
11	CAO6	Veränderung der Zusammenarbeit mit Kolleg*innen	Zusammenarbeit mit Kolleg*innen (z.B. Absprachen oder zur Unterrichtsvorbereitung)	/Ja<br>/Nein	eigenes Item	-	-	-	-	-	-	-
12	CAO6a	Bewertung	(Filterfrage, falls „Ja") Empfinden Sie diese Veränderung als belastend?	/in sehr hohem Maße<br>/in hohem Maße<br>/zum Teil<br>/in geringem Maße<br>/in sehr geringem Maße<br>/gar nicht	eigenes Item	-	-	-	negativ	-	-	-
13	CAO7	Veränderung der Bereitstellung von Arbeits- / Unterrichtsmatierial	Gegenseitiges Bereitstellen von Arbeits- und Unterrichtsmaterialien im Kollegium.	/Ja<br>/Nein	angelehnt an BUGIS-R-2011 (Item 2.10)	-	nein	nein	-	Niedersächsisches Kultusministerium (2011) - bugis R-2011	-	-
14	CAO7a	Bewertung	(Filterfrage, falls „Ja") Empfinden Sie diese Veränderung als belastend?	/in sehr hohem Maße<br>/in hohem Maße<br>/zum Teil<br>/in geringem Maße<br>/in sehr geringem Maße<br>/gar nicht	eigenes Item	-	-	-	negativ	-	-	-
15	CAO8	Veränderung Erhalt relevanter Informationen	Erhalt aller Informationen, die Sie für die Ausübung Ihrer Tätigkeit benötigen	/Ja<br>/Nein	Angelehnt an COPSOQ-3, Skala Vorhersehbarkeit der Arbeit	Vorhersehbarkeit der Arbeit	nein	nein	-	-	-	-
16	CAO8a	Bewertung	(Filterfrage, falls „Ja") Empfinden Sie diese Veränderung als belastend?	/in sehr hohem Maße<br>/in hohem Maße<br>/zum Teil<br>/in geringem Maße<br>/in sehr geringem Maße<br>/gar nicht	eigenes Item	-	-	-	negativ	-	-	-
17	CAO9	Veränderung der Informationsmenge für schulische Belange	Zunahme der Informationsmenge für schulische Belange (z.B. E-Mails, Messenger, Aushänge, Mündliches)	/Ja<br>/Nein	eigenes Item	-	-	-	-	-	-	-
18	CAO9a	Bewertung	(Filterfrage, falls „Ja") Empfinden Sie diese Veränderung als belastend?	/in sehr hohem Maße<br>/in hohem Maße<br>/zum Teil<br>/in geringem Maße<br>/in sehr geringem Maße<br>/gar nicht	eigenes Item	-	-	-	negativ	-	-	-
19	CAO10	Veränderung Konferenzen / Dienstbesprechungen	Konferenzen und Dienstbesprechungen	/Ja<br>/Nein	angelehnt an BUGIS-R-2011 (Item 1.1)	-	nein	nein	-	-	-	-

20	CAO10a	Bewertung	(Filterfrage, falls „Ja“)<br>Empfinden Sie diese Veränderung als belastend?	/in sehr hohem Maße<br>/in hohem Maße<br>/zum Teil<br>/in geringem Maße<br>/in sehr geringem Maße<br>/gar nicht	eigenes Item	-	-	-	negativ	-	-	-
21	CAO11	Veränderung Klarheit des Verantwortungsbereichs	Klarheit bezüglich Ihres Verantwortungsbereichs	/Ja<br>/Nein	angelehnt an COPSOQ-3 Skala<br>Rollenklarheit	-	nein	nein	-	-	-	-
22	CAO11a	Bewertung	(Filterfrage, falls „Ja“)<br>Empfinden Sie diese Veränderung als belastend?	/in sehr hohem Maße<br>/in hohem Maße<br>/zum Teil<br>/in geringem Maße<br>/in sehr geringem Maße<br>/gar nicht	eigenes Item	-	-	-	negativ	-	-	-
23	CAO12	Veränderung der emotionalen Anforderungen	Mehr emotionale Anforderungen der Arbeit (z B. Verbergen von Gefühlen)	/Ja<br>/Nein	angelehnt an COPSOQ-3 Skala<br>emotionale Anforderungen	-	nein	nein	-	-	-	-
24	CAO12a	Bewertung	(Filterfrage, falls „Ja“)<br>Empfinden Sie diese Veränderung als belastend?	/in sehr hohem Maße<br>/in hohem Maße<br>/zum Teil<br>/in geringem Maße<br>/in sehr geringem Maße<br>/gar nicht	eigenes Item	-	-	-	negativ	-	-	-
25	CAO13	Veränderung Arbeitsmenge	Erhöhung der Arbeitsmenge	/Ja<br>/Nein	Angelehnt an<br>GFB Cockpit IfL	-	-	-	-	-	-	-
26	CAO13a	Bewertung	(Filterfrage, falls „Ja“)<br>Empfinden Sie diese Veränderung als belastend?	/in sehr hohem Maße<br>/in hohem Maße<br>/zum Teil<br>/in geringem Maße<br>/in sehr geringem Maße<br>/gar nicht	eigenes Item	-	-	-	negativ	-	-	-
27	CAO14	Vertrauen & Gerechtigkeit	Gerechte Konfliktlösung durch die Schulleitung	/Ja<br>/Nein	angelehnt an die COPSOQ-Skala „Vertrauen & Gerechtigkeit “		nein	nein	-	https://www.copsoq.de/copsoq-fragebogen/	Ramkissoon (2019) - Dissecting the effect of workplace exposures on workers'rating of psychological health and safety	Bethelsen et al. (2020) - Validation of the Copenhagen Psychosocial Questionnaire Version III
28	CAO14a	Bewertung	(Filterfrage, falls „Ja“)<br>Empfinden Sie diese Veränderung als belastend?	/in sehr hohem Maße<br>/in hohem Maße<br>/zum Teil<br>/in geringem Maße<br>/in sehr geringem Maße<br>/gar nicht	eigenes Item		-	-	negativ	-	-	-
29	CAO15	Vertrauen & Gerechtigkeit	Gerechte Verteilung der Arbeit	/Ja<br>/Nein	angelehnt an die COPSOQ-Skala „Vertrauen & Gerechtigkeit “	Vertrauen & Gerechtigkeit	nein	nein	-	https://www.copsoq.de/copsoq-fragebogen/	Ramkissoon (2019) - Dissecting the effect of workplace exposures on workers'rating of psychological health and safety	Bethelsen et al. (2020) - Validation of the Copenhagen Psychosocial Questionnaire Version III
30	CAO15a	Bewertung	(Filterfrage, falls „Ja“)<br>Empfinden Sie diese Veränderung als belastend?	/in sehr hohem Maße<br>/in hohem Maße<br>/zum Teil<br>/in geringem Maße<br>/in sehr geringem Maße<br>/gar nicht	eigenes Item		-	-	negativ	-	-	-
31	CAO16	Wertschätzung	Anerkennung und Wertschätzung Ihrer Arbeit durch die Schulleitung	/Ja<br>/Nein	angelehnt an die COPSOQ-Skala „Vertrauen & Gerechtigkeit “		nein	ja	-	https://www.copsoq.de/copsoq-fragebogen/	Ramkissoon (2019) - Dissecting the effect of workplace exposures on workers'rating of psychological health and safety	Bethelsen et al. (2020) - Validation of the Copenhagen Psychosocial Questionnaire Version III
32	CAO16a	Bewertung	(Filterfrage, falls „Ja“)<br>Empfinden Sie diese Veränderung als belastend?	/in sehr hohem Maße<br>/in hohem Maße<br>/zum Teil<br>/in geringem Maße<br>/in sehr geringem Maße<br>/gar nicht	eigenes Item		-	-	negativ	-	-	-

2b) Pädagogisch

Pädagogische Belastungen und Herausforderungen für Lehrkräfte

Erleben Sie aufgrund der COVID-19-Pandemie Veränderungen in nachfolgend genannten Bereichen im Vergleich zur Zeit vor der COVID-19-Pandemie?

37	CAP1	Veränderung durch Distanzunterricht	Probleme bei der Umsetzung des Bildungsauftrags	/Ja<br>/Nein	eigenes Item	-	-	-	-	-	-	-
38	CAP1a	Bewertung	(Filterfrage, falls „Ja“)<br>Empfinden Sie diese Veränderung als belastend?	/in sehr hohem Maße<br>/in hohem Maße<br>/zum Teil<br>/in geringem Maße<br>/in sehr geringem Maße<br>/gar nicht	eigenes Item	-	-	-	negativ	-	-	-
39	CAP2	Veränderung Didaktik Präsenz	Notwendigkeit zur Änderung in der Didaktik des Präsenzunterrichts	/Ja<br>/Nein	eigenes Item	-	-	-	-	-	-	-
40	CAP2a	Bewertung	(Filterfrage, falls „Ja“)<br>Empfinden Sie diese Veränderung als belastend?	/in sehr hohem Maße<br>/in hohem Maße<br>/zum Teil<br>/in geringem Maße<br>/in sehr geringem Maße<br>/gar nicht	eigenes Item	-	-	-	negativ	-	-	-

41	CAP3	Veränderung Umsetzung Bildungsauftrag	Schwierigkeiten bei der Umsetzung von Distanzunterricht	/Ja<br>/Nein	eigenes Item	-	-	-	-	-	-	-
42	CAP3a	Bewertung	(Filterfrage, falls „Ja") Empfinden Sie diese Veränderung als belastend?	/in sehr hohem Maße<br>/in hohem Maße<br>/zum Teil<br>/in geringem Maße<br>/in sehr geringem Maße<br>/gar nicht	eigenes Item	-	-	-	negativ	-	-	-

Belastungen und Herausforderungen bei der Umsetzung des Bildungsauftrages

Erleben Sie aufgrund der COVID-19-Pandemie Veränderungen in nachfolgend genannten Bereichen im Vergleich zur Zeit vor der COVID-19-Pandemie?

42	CAP4	Veränderung schulische Entwicklung Schüler*innen	Eine Verschlechterung der <u>schulischen</u> Entwicklung Ihrer Schüler*innen	/Ja<br>/Nein	eigenes Item	-	-	-	-	-	-	-
43	CAP4a	Bewertung	(Filterfrage, falls „Ja") Empfinden Sie diese Veränderung als belastend?	/in sehr hohem Maße<br>/in hohem Maße<br>/zum Teil<br>/in geringem Maße<br>/in sehr geringem Maße<br>/gar nicht	eigenes Item	-	-	-	negativ	-	-	-
44	CAP5	Veränderung persönliche Entwicklung Schüler*innen	Eine Verschlechterung der <u>persönlichen</u> Entwicklung Ihrer Schüler*innen	/Ja<br>/Nein	eigenes Item	-	-	-	-	-	-	-
45	CAP5a	Bewertung	(Filterfrage, falls „Ja") Empfinden Sie diese Veränderung als belastend?	/in sehr hohem Maße<br>/in hohem Maße<br>/zum Teil<br>/in geringem Maße<br>/in sehr geringem Maße<br>/gar nicht	eigenes Item	-	-	-	negativ	-	-	-
46	CAP6	Veränderung Kontakt zu Schüler*innen	Einen schlechteren Kontakt zu Ihren Schüler*innen	/Ja<br>/Nein	eigenes Item	-	-	-	-	-	-	-
47	CAP6a	Bewertung	(Filterfrage, falls „Ja") Empfinden Sie diese Veränderung als belastend?	/in sehr hohem Maße<br>/in hohem Maße<br>/zum Teil<br>/in geringem Maße<br>/in sehr geringem Maße<br>/gar nicht	eigenes Item	-	-	-	negativ	-	-	-
48	CAP7	Veränderung Kontakt zu Erziehungsberechtigten	Einen schlechteren Kontakt zu den Erziehungsberechtigten Ihrer Schüler*innen	/Ja<br>/Nein	eigenes Item	-	-	-	-	-	-	-
49	CAP7a	Bewertung	(Filterfrage, falls „Ja") Empfinden Sie diese Veränderung als belastend?	/in sehr hohem Maße<br>/in hohem Maße<br>/zum Teil<br>/in geringem Maße<br>/in sehr geringem Maße<br>/gar nicht	eigenes Item	-	-	-	negativ	-	-	-
50	CAP8	Veränderung Erwartungen von Erziehungsberechtigten	Höhere Erwartungen der Erziehungsberechtigten Ihrer Schüler*innen an Ihre Arbeit	/Ja<br>/Nein	eigenes Item	-	-	-	-	-	-	-
51	CAP8a	Bewertung	(Filterfrage, falls „Ja") Empfinden Sie diese Veränderung als belastend?	/in sehr hohem Maße<br>/in hohem Maße<br>/zum Teil<br>/in geringem Maße<br>/in sehr geringem Maße<br>/gar nicht	eigenes Item	-	-	-	negativ	-	-	-
52	CAP9	Veränderung Erreichung Lernziele	Schwierigkeiten beim <u>Erreichen der angestrebten Lernziele</u> mit Ihren Schüler*innen	/Ja<br>/Nein	eigenes Item	-	-	-	-	-	-	-
53	CAP9a	Bewertung	(Filterfrage, falls „Ja") Empfinden Sie diese Veränderung als belastend?	/in sehr hohem Maße<br>/in hohem Maße<br>/zum Teil<br>/in geringem Maße<br>/in sehr geringem Maße<br>/gar nicht	eigenes Item	-	-	-	negativ	-	-	-

2d) Umstellung auf digitale Unterrichtsformate / Homeschooling

Distanzunterricht

54	CAD1	Umstellung auf Distanzunterricht	Haben Sie Ihren Unterricht während der COVID-19-Pandemie (phasenweise) von Präsenzunterricht auf Distanzunterricht umgestellt?	/Ja<br>/Nein	eigenes Item	-	-	-	-	-	-	-
55	CAD1a	Bewertung	(Filterfrage, falls „Ja") Empfinden Sie diese Veränderung als belastend?	/in sehr hohem Maße<br>/in hohem Maße<br>/zum Teil<br>/in geringem Maße<br>/in sehr geringem Maße<br>/gar nicht	eigenes Item							
56	CAD2	Aktueller Unterrichtsmodus	In welchem Modus unterrichten Sie aktuell?	/Präsenzunterricht<br>/Distanzunterricht<br>/Beide Formen	eigenes Item	-	-	-	-	-	-	-
57	CAD3	Umstellung auf Distanzunterricht	(Filterfragen folgend, falls CAD1 = Ja ODER CAD2 = Distanzunterricht ODER CAD2 = Beide Formen, dann CAD3-CAD8 anzeigen)<br>**Welche Formen der Unterstützung haben Sie Ihren Schüler*innen während des Distanzunterrichts angeboten?**<br>(Mehrfachnennungen möglich)	/Online-Lernplattformen (z.B. Moodle)<br>/Videos aus dem Internet<br>/selbst erstellte Videos<br>/Papierausdruck (Download)<br>/Papierausdruck (per Post oder zur Abholung)<br>/Internetseiten<br>/E-Mail-Kontakte<br>/Messenger-Kontakte<br>/Telefonate<br>/gemeinsamer webbasierter Unterricht (z. B. per Videoanruf)<br>/persönliche Gespräche<br>/Präsentationen<br>/Apps	Angelehnt an: Bundesweite Elternbefragung zu Homeschooling während der Covid 19-Pandemie.Zentrum für Empirische Pädagogische Forschung / Institut für Bildung im Kindes- und Jugendalter / Uni Koblenz-Landau, 2021	-	-	-	-	https://www.zepf.eu/wp-content/uploads/2020/06/Bericht_HOMEschooling2020.pdf	-	-

58	CAD4	Austausch bei Distanzunterricht	Wie häufig tauschten Sie sich durchschnittlich mit Ihren Schüler*innen während des Distanzunterrichts aus?<br>(Mehrfachnennungen möglich)	/mehrmals täglich<br>/einmal täglich<br>/mehrmals pro Woche<br>/einmal pro Woche<br>/weniger als einmal pro Woche<br>/fast nie / nie	Angelehnt an: Bundesweite Elternbefragung zu Homeschooling während der Covid 19-Pandemie.Zentrum für Empirische Pädagogische Forschung / Institut für Bildung im Kindes- und Jugendalter / Uni Koblenz-Landau, 2021	-	-	-	positiv	https://www.zepf.eu/wp-content/uploads/2020/06/Bericht_HOMEschooling2020.pdf	-	-
59	CAD5	Technische Probleme Distanzunterricht	Der Distanzunterricht wurde von technischen Problemen erschwert (z. B. Verbindungsabbrüche, Softwarefehler, Probleme bei der Bedienung).	/Ja<br>/Nein	eigenes Item	-	-	-	negativ	-	-	-
60	CAD5a	Bewertung	(Filterfrage, falls „Ja“)<br>Empfinden Sie diese Veränderung als belastend?	/in sehr hohem Maße<br>/in hohem Maße<br>/zum Teil<br>/in geringem Maße<br>/in sehr geringem Maße<br>/gar nicht	eigenes Item	-	-	-	negativ	-	-	-
61	CAD6	Technische Ausstattung Distanzunterricht	Der Distanzunterricht wurde durch unzureichende technische Ausstattung erschwert (z. B. nicht vorhandene oder veraltete Geräte).	/Ja<br>/Nein	eigenes Item	-	-	-	negativ	-	-	-
62	CAD6a	Bewertung	(Filterfrage, falls „Ja“)<br>Empfinden Sie diese Veränderung als belastend?	/in sehr hohem Maße<br>/in hohem Maße<br>/zum Teil<br>/in geringem Maße<br>/in sehr geringem Maße<br>/gar nicht	eigenes Item	-	-	-	negativ	-	-	-
63	CAD6b	Bewertung	Falls CAD6 „zum Teil“ODER "in hohem Maße" ODER " in sehr hohem Maße":<br>Durch wessen technische Ausstattung wurde die Umstellung auf Distanzunterricht erschwert?<br>(Mehrfachnennungen möglich)	/Technische Ausstattung der Schüler*innen<br>/Schule<br>/Eigene technische Ausstattung	eigenes Item	-	-	-	negativ	-	-	-
64	CAD7	Überforderung durch Distanzunterricht	Der Distanzunterricht brachte mit sich, dass Sie sich häufig von den Aufgaben überfordert fühlten.	/Ja<br>/Nein	Eng angelehnt an Items zu Qualifikationsmängeln aus dem FGBU	Qualifikationsmängel	nein	nein	negativ	Dettmers, Jan & Krause, Andreas. (2020). Der Fragebogen zur Gefährdungsbeurteilung psychischer Belastungen (FGBU). Zeitschrift für Arbeits- und Organisationspsychologie. 64. 99-119. 10.1026/0932-4089/a000318.	-	-
65	CAD7a	Bewertung	(Filterfrage, falls „Ja“)<br>Empfinden Sie diese Veränderung als belastend?	/in sehr hohem Maße<br>/in hohem Maße<br>/zum Teil<br>/in geringem Maße<br>/in sehr geringem Maße<br>/gar nicht	eigenes Item	-	-	-	negativ	-	-	-
66	CAD8	Probleme der Leistungsbewertung im Distanzunterricht	Der Distanzunterricht erschwerte die Leistungsbewertung Ihrer Schüler*innen.	/Ja<br>/Nein	eigenes Item	-	-	-	negativ	-	-	-
67	CAD8a	Bewertung	(Filterfrage, falls „Ja“)<br>Empfinden Sie diese Veränderung als belastend?	/in sehr hohem Maße<br>/in hohem Maße<br>/zum Teil<br>/in geringem Maße<br>/in sehr geringem Maße<br>/gar nicht	eigenes Item	-	-	-	negativ	-	-	-
Einstellungen zu digitalen Unterrichtsformaten												
68	CAD9	Nutzung digitaler Unterrichtsformate in Vergangenheit	Wie oft haben Sie **vor Beginn der COVID-19-Pandemie** digitale Unterrichtsformate eingesetzt? (Einsatz von Laptops / Tablets, Online-Lernplattformen, Bereitstellung von Downloads etc.)	/Täglich<br>/Mehrmals in der Woche<br>/Etwa einmal in der Woche<br>/Etwa einmal im Monat<br>/Seltener als einmal im Monat<br>/Fast nie / nie	eigenes Item	-	-	-	positiv	-	-	-
69	CAD10	Bewertung digitaler Unterrichtsformate	Die Nutzung digitaler Unterrichtsformate...<br>**… bewerte ich insgesamt positiv.**	/Stimme überhaupt nicht zu<br>/Stimme eher nicht zu<br>/Teils/teils<br>/Stimme eher zu<br>/Stimme voll und ganz zu<br>/Keine Antwort	eigenes Item	-	-	-	positiv	-	-	-
70	CAD11	Bewertung digitaler Unterrichtsformate bzgl. Leistungsstand Schüler*innen	Die Nutzung digitaler Unterrichtsformate...<br>**… hatte positive Auswirkungen auf den Leistungsstand meiner Schüler*innen.**	/Stimme überhaupt nicht zu<br>/Stimme eher nicht zu<br>/Teils/teils<br>/Stimme eher zu<br>/Stimme voll und ganz zu<br>/Keine Antwort	eigenes Item	-	-	-	positiv	-	-	-
71	CAD12	Umstellung auf digitale Unterrichtsformate	Die Nutzung digitaler Unterrichtsformate...<br>**… verstärkte soziale Ungleichheit zwischen Schüler*innen (z. B. aufgrund unterschiedlicher Unterstützungsmöglichkeiten durch deren Erziehungsberechtigte).**	/Stimme überhaupt nicht zu<br>/Stimme eher nicht zu<br>/Teils/teils<br>/Stimme eher zu<br>/Stimme voll und ganz zu<br>/Keine Antwort	angelehnt an "Das Deutsche Schulbarometer Spezial Corona-Krise" FORSA 04/2019	Lernrückstände und soziale Ungleichheit	nein	nein	negativ	https://www.bosch-stiftung.de/de/news/das-deutsche-schulbarometer-coronakrise-zeigt-nachholbedarf-bei-digitalen-lernformaten	-	-

72	CAD13	Umstellung auf digitale Unterrichtsformate	Die Nutzung digitaler Unterrichtsformate...<br>**… stellt eine Chance dar.**	/Stimme überhaupt nicht zu<br>/Stimme eher nicht zu<br>/Teils/teils<br>/Stimme eher zu<br>/Stimme voll und ganz zu<br>/Keine Antwort	eigenes Item	-	-	-	positiv	-	-	-
73	CAD13a	Umstellung auf digitale Unterrichtsformate	Falls CAD13 teils / teils oder darüber:<br>**Für wen stellt die Umstellung auf digitale Unterrichtsformate eine Chance dar?**<br>(Mehrfachnennung möglich)	/Schüler*innen<br>/Familien der Schüler*innen<br>/Schulen<br>/Lehrkräfte<br>/Gesellschaft insgesamt	eigenes Item	-	-	-	-	-	-	-
74	CAD14	Gesamtkonzept digitaler Unterricht	An Ihrer Schule / Dienststelle liegt ein einheitliches Gesamtkonzept zur Durchführung digitalen Unterrichts vor	/Stimme überhaupt nicht zu<br>/Stimme eher nicht zu<br>/Teils/teils<br>/Stimme eher zu<br>/Stimme voll und ganz zu<br>/Keine Antwort	eigenes Item	-	-	-	-	-	-	-
75	CAD15	Rückmeldung Lernergebnisse in digitalen Unterrichtsformaten	Sie haben Schüler*innen Rückmeldungen zu deren Lernergebnissen aus digitalen Unterrichtsformaten gegeben.	/Stimme überhaupt nicht zu<br>/Stimme eher nicht zu<br>/Teils/teils<br>/Stimme eher zu<br>/Stimme voll und ganz zu<br>/Keine Antwort	Angelehnt an: Bundesweite Elternbefragung zu Homeschooling während der Covid 19-Pandemie.Zentrum für Empirische Pädagogische Forschung / Institut für Bildung im Kindes- und Jugendalter / Uni Koblenz-Landau, 2020	-	-	-	positiv	https://www.zepf.eu/wp-content/uploads/2020/06/Bericht_HOMEschooling2020.pdf	-	-
76	BEL	Weitere Belastungen / Herausforderungen	Alles in allem, welche noch nicht erfragten Belastungen oder Herausforderungen stehen für Sie in Zusammenhang mit der COVID-19-Pandemie?<br>*(Bitte formulieren Sie Ihre Antwort knapp / in Stichworten)*	[Freitext]	eigenes Item	-	-	-	-	-	-	-

3. Umsetzung, Kommunikation und Einhaltung von Hygieneplänen

3a) Persönlich

1	IFP1	Bewertung aktuelle Maßnahmen gegen Corona-Virus	Wie bewerten Sie die getroffenen Maßnahmen (AHA-Regeln) zur Eindämmung des SARS-CoV-2-Virus?	/Absolut sinnlos und unangemessen<br>/Überwiegend sinnlos und unangemessen<br>/Weder noch<br>/Überwiegend sinnvoll und angemessen<br>/Absolut sinnvoll und angemessen	Aus Befragung der Medizinische Hochschule Hannover	-	ja	-	-	https://ww2.unipark.de/uc/MHH_Umfrage_COVID-19/	-	-
2	IFP2	Bewertung Mund-Nasen-Bedeckung	Wie schätzen Sie das Tragen einer Mund-Nase-Bedeckung ein?	/Absolut sinnlos und unangemessen<br>/Überwiegend sinnlos und unangemessen<br>/Weder noch<br>/Überwiegend sinnvoll und angemessen<br>/Absolut sinnvoll und angemessen	eigenes Item	-	-	-	-	-	-	-
3	IFP3	Bewertung Abstandsregeln	Wie bewerten Sie die Vorgabe zur Einhaltung von Abstandsregeln?	/Absolut sinnlos und unangemessen<br>/Überwiegend sinnlos und unangemessen<br>/Weder noch<br>/Überwiegend sinnvoll und angemessen<br>/Absolut sinnvoll und angemessen	eigenes Item	-	-	-	-	-	-	-
4	IFP4	Einstellung zu Schulschließungen	Wie bewerten Sie frühzeitige Schulschließungen im Falle steigender COVID-19-Fallzahlen als Maßnahme?	/Absolut sinnlos und unangemessen<br>/Überwiegend sinnlos und unangemessen<br>/Weder noch<br>/Überwiegend sinnvoll und angemessen<br>/Absolut sinnvoll und angemessen	eigenes Item				-	-	-	-
5	IFP5	Nutzung Corona-Warn-App	Haben Sie die Corona-Warn-App installiert?	/Ja<br>/Nein	eigenes Item	-	-	-	-	-	-	-
6	IFP6	Intensität Gesundheitsbezogenes Informationsverhalten	Wenn Sie einmal an eine ganz gewöhnliche Woche während der Corona-Pandemie denken: An wie vielen Tagen dieser Woche informieren Sie sich über das Thema Corona?	An [Freitext] Tagen	eigenes Item	Intensität Gesundheitsbezogenes Informationsverhalten	-	-	-	-	-	-

7	IFP7	Gesundheitsbezogene Informationsquellen allgemein	Was sind für Sie die relevantesten Informationsquellen, aus denen Sie verbindliche Informationen rund um das Thema COVID-19-Pandemie beziehen?<br><br>(Mehrfachnennung möglich)	/in persönlichen Gesprächen mit Ärzt*innen, Therapeut*innen, Pflegekräften<br>/in persönlichen Gesprächen mit Apotheker*innen<br>/in persönlichen Gesprächen mit Familienangehörigen, Freund*innen, Kolleg*innen<br>/in persönlichen Gesprächen mit anderen Patient*innen oder Betroffenen (z. B. in einer Selbsthilfegruppe, im Wartezimmer)<br>/in Beratungsstellen, Gesundheits- oder Bildungseinrichtungen<br>/am Telefon bei Krankenkassen, Patienten- oder Verbraucherschutzorganisationen<br>/in kostenlosen Broschüren oder Zeitschriften von Krankenkassen, Apotheken oder anderen Anbietern (Printausgaben; offline)<br>/in Büchern, Gesundheitsratgebern, Lexika<br>/in Zeitungen oder Zeitschriften (z. B. Printausgabe der Tageszeitung; offline)<br>/im Radio (z. B. Auto- oder Küchenradio; offline)<br>/im Fernsehen (z. B. Kabel- oder Satelliten-Fernsehen; offline)<br>/auf sonstigem Wege (offline)<br>/Wikipedia oder andere Online-Lexika<br>/Internetseiten von Krankenkassen<br>/Gesundheitsportale (z. B. netdoktor, onmeda, gesundheit.de)<br>/Webseiten von Ärzt*innen, Krankenhäusern, Reha- oder Pflegeeinrichtungen<br><br>/Internetauftritte von Fachstellen (z. B. RKI, BAuA, UBA usw.)<br>/Ratgeber-Communitys (z. B. gutefrage.de, wer-weiss-was.de)<br>/Online- / Internetapotheken<br>/Gesundheitsforen und Communitys speziell zu Gesundheits- und Krankheitsthemen<br>/Vergleichsportale zur Suche von Ärzt*innen, Krankenhäusern und Pflegeheimen (z. B. Weiße Liste)<br>/Social Media (z. B. Facebook, Instagram, Snapchat, Twitter usw.)<br>/Blogs zu Gesundheitsthemen<br>/Webseiten gemeinnütziger Gesundheitsorganisationen, unabhängiger Patienten- oder Selbsthilfeorganisationen<br>/Medizinische Online-Beratung (z. B. Online-Sprechstunden von Ärzt*innen oder Krankenhäusern)<br>/Online-Nachrichtenseiten (z. B. tagesschau.de, Spiegel.de, bild.de, Zeit.de, faz.net)<br>/Videoplattformen (z. B. YouTube)<br>/Online-Radio, Audio-Streaming & Podcasts<br>/Online-TV & Video-Streaming (z. B. Netflix, Amazon Prime Video usw.)<br>/Suchmaschinen (z. B. Google)<br>/Sonstige Online-Angebote	Gesundheitsmonitor, z.B. Marstedt, 2018	Gesundheitsbezogene Informationsquellen allgemein	-	-	-	-	-	-
3b) Schulspezifisch												
8	CHP1	Zufriedenheit Hygienepläne der Landesbehörden	Durch die Hygienepläne der für Ihre Schule / Dienststelle zuständigen Landesbehörde zum Infektionsschutz an Schulen fühlen Sie sich gut geschützt.	/Stimme überhaupt nicht zu<br>/Stimme eher nicht zu<br>/Teils/teils<br>/Stimme eher zu<br>/Stimme voll und ganz zu<br>/Keine Antwort	eigenes Item	-	-	-	positiv	-	-	-
9	CHP2	Zufriedenheit Umsetzung Hygienepläne	Sie sind mit der Umsetzung der Hygienepläne durch die Schulleitung an Ihrer Schule zufrieden.	/Stimme überhaupt nicht zu<br>/Stimme eher nicht zu<br>/Teils/teils<br>/Stimme eher zu<br>/Stimme voll und ganz zu<br>/Keine Antwort	eigenes Item	-	-	-	positiv	-	-	-
10	CHP3	Information über Hygienepläne	Sie fühlen sich über Hygienepläne zum Infektionsschutz an Ihrer Schule / Dienststelle gut informiert.	/Stimme überhaupt nicht zu<br>/Stimme eher nicht zu<br>/Teils/teils<br>/Stimme eher zu<br>/Stimme voll und ganz zu<br>/Keine Antwort	eigenes Item	-	-	-	positiv	-	-	-
11	CHP4	Information über Hygienepläne Schüler*innen und Erziehungsberechtigte	Schüler*innen und deren Erziehungsberechtigte sind gut über die jeweils gültigen Hygienepläne informiert.	/Stimme überhaupt nicht zu<br>/Stimme eher nicht zu<br>/Teils/teils<br>/Stimme eher zu<br>/Stimme voll und ganz zu<br>/Keine Antwort	eigenes Item	-	-	-	positiv	-	-	-
12	CHP5	Teilnahme Infektionsschutzbelehrung	Wie lange liegt Ihre letzte Teilnahme an einer Infektionsschutzbelehrung zurück?	/<1 Jahr<br>/1-2 Jahre<br>/2-3 Jahre<br>/3-5 Jahre<br>/5-10 Jahre<br>/>10 Jahre<br>/noch nie teilgenommen<br>/ich weiß nicht	angelehnt an §35 Infektionsschutzgesetz	-	-	-	negativ	-	-	-
13	CHP6	Beurteilung Arbeitsschutz	Wurde an Ihrer Schule bereits eine Gefährdungsbeurteilung durchgeführt?	/Ja<br>/Nein<br>/Ich weiß nicht	eigenes Item							

14	CHP7	Beurteilung Arbeitsschutz	Wurde eine Gefährdungsbeurteilung im Rahmen der COVID-19-Pandemie aktualisiert?	/Ja<br>/Nein<br>/Ich weiß nicht	eigenes Item							
15	CHP8	Sicherheitsunterweisung	Fanden aufgrund der COVID-19-Pandemie Unterweisungen zu den besonderen Gefährdungen statt?	/Ja<br>/Nein<br>/Ich weiß nicht	eigenes Item							
16	CHP9	Sicherheitsunterweisung	Fanden aufgrund der COVID-19-Pandemie Unterweisungen zum Arbeiten im Home-Office statt?	/Ja<br>/Nein<br>/Ich weiß nicht	eigenes Item							
17	CHP10	Sicherheitsunterweisung	Fanden aufgrund der COVID-19-Pandemie Unterweisungen zum Schutz besonders gefährdeter Personen statt?	/Ja<br>/Nein<br>/Ich weiß nicht	eigenes Item							
18	CHP11	Gebrauch von Mund-Nase-Bedeckung	Wurden Sie von Ihrem Dienstherren über den korrekten Gebrauch von Mund-Nase-Bedeckungen informiert?	/Ja<br>/Nein<br>/Ich weiß nicht	eigenes Item							
19	CHP12	Maskenpausen	Wurden in Ihrem Schulaltag Zeiten realisiert, in denen keine Mund-Nase-Bedeckung getragen wurde (sog. "Maskenpausen")?	/Ja<br>/Nein<br>/Ich weiß nicht	eigenes Item							
20	CHC1	Einhaltung der Hygienepläne	An Ihrer Schule / Dienststelle werden die vorgeschriebenen Hygienepläne zum Umgang mit der COVID-19-Pandemie insgesamt eingehalten.	/Stimme überhaupt nicht zu<br>/Stimme eher nicht zu<br>/Teils/teils<br>/Stimme eher zu<br>/Stimme voll und ganz zu<br>/Keine Antwort	eigenes Item	-	-	-	positiv	-	-	-
21	CHC2	Einhaltung der Hygienepläne	Die vorgeschriebenen Hygienepläne werden eingehalten von…<br>**… Schüler*innen**	/Stimme überhaupt nicht zu<br>/Stimme eher nicht zu<br>/Teils/teils<br>/Stimme eher zu<br>/Stimme voll und ganz zu<br>/Keine Antwort	eigenes Item	-	-	-	positiv	-	-	-
22	CHC3	Einhaltung der Hygienepläne	Die vorgeschriebenen Hygienepläne werden eingehalten von…<br>**… Erziehungsberechtigten der Schüler*innen**	/Stimme überhaupt nicht zu<br>/Stimme eher nicht zu<br>/Teils/teils<br>/Stimme eher zu<br>/Stimme voll und ganz zu<br>/Keine Antwort	eigenes Item	-	-	-	positiv	-	-	-
23	CHC4	Einhaltung der Hygienepläne	Die vorgeschriebenen Hygienepläne werden eingehalten von…<br>**… Lehrkräfte**n	/Stimme überhaupt nicht zu<br>/Stimme eher nicht zu<br>/Teils/teils<br>/Stimme eher zu<br>/Stimme voll und ganz zu<br>/Keine Antwort	eigenes Item	-	-	-	positiv	-	-	-
24	CHC5	Einhaltung der Hygienepläne	Die vorgeschriebenen Hygienepläne werden eingehalten von…<br>**… Hausmeister / Reinigungskräften**	/Stimme überhaupt nicht zu<br>/Stimme eher nicht zu<br>/Teils/teils<br>/Stimme eher zu<br>/Stimme voll und ganz zu<br>/Keine Antwort	eigenes Item	-	-	-	positiv	-	-	-
25	CHC6	Einhaltung der Hygienepläne	Die vorgeschriebenen Hygienepläne werden eingehalten von…<br>**… Sonstigen Personen [Freitext]**	/Stimme überhaupt nicht zu<br>/Stimme eher nicht zu<br>/Teils/teils<br>/Stimme eher zu<br>/Stimme voll und ganz zu<br>/Keine Antwort	eigenes Item	-	-	-	positiv	-	-	-
26	CHC	Personalausfall	Kam es an Ihrer Schule zu Personalausfällen aufgrund von COVID-19 (z. B. wegen Zugehörigkeit zu Risikogruppen)	/Ja<br>/Nein	Angelehnt an Thema aus Corona-KiTa Befragung	-	-	-	negativ	-	-	-
27	CHC7a	Bewertung	(Filterfrage, falls „Ja“)<br>Empfinden Sie diese Veränderung als belastend?	/in sehr hohem Maße<br>/in hohem Maße<br>/zum Teil<br>/in geringem Maße<br>/in sehr geringem Maße<br>/gar nicht	eigenes Item	-	-	-	negativ	-	-	-
28	ABC1a	Stoß- / Querlüftungsmöglichkeit	Eine Stoß- bzw. Querlüftung oder der Betrieb vorhandener raumlufttechnischer Anlagen ist möglich …<br>**… in Klassenräumen**	/In keinem Fall<br>/Stimme eher nicht zu<br>/Teils/teils<br>/Stimme eher zu<br>/Stimme voll und ganz zu<br>/Keine Antwort	eigenes Item	-	-	-	positiv	-	-	-
29	ABC1b	Stoß- / Querlüftungsmöglichkeit	Eine Stoß- bzw. Querlüftung oder der Betrieb vorhandener raumlufttechnischer Anlagen ist möglich …<br>**… auf Fluren**	/In keinem Fall<br>/Stimme eher nicht zu<br>/Teils/teils<br>/Stimme eher zu<br>/Stimme voll und ganz zu<br>/Keine Antwort	eigenes Item	-	-	-	positiv	-	-	-
30	ABC1c	Stoß- / Querlüftungsmöglichkeit	Eine Stoß- bzw. Querlüftung oder der Betrieb vorhandener raumlufttechnischer Anlagen ist möglich …<br>**… im Lehrerzimmer**	/In keinem Fall<br>/Stimme eher nicht zu<br>/Teils/teils<br>/Stimme eher zu<br>/Stimme voll und ganz zu<br>/Keine Antwort	eigenes Item	-	-	-	positiv	-	-	-
31	ABC1d	Stoß- / Querlüftungsmöglichkeit	Eine Stoß- bzw. Querlüftung oder der Betrieb vorhandener raumlufttechnischer Anlagen ist möglich …<br>**… in sanitären Einrichtungen**	/In keinem Fall<br>/Stimme eher nicht zu<br>/Teils/teils<br>/Stimme eher zu<br>/Stimme voll und ganz zu<br>/Keine Antwort	eigenes Item	-	-	-	positiv	-	-	-

32	ABC1e	Stoß- / Querlüftungsmöglichkeit	Eine Stoß- bzw. Querlüftung oder der Betrieb vorhandener raumlufttechnischer Anlagen ist möglich …<br><br>**… in der Mensa / im Speisesaal**	/In keinem Fall<br>/Stimme eher nicht zu<br>/Teils/teils<br>/Stimme eher zu<br>/Stimme voll und ganz zu<br>/Keine Antwort	eigenes Item							
33	ABC1e	Stoß- / Querlüftungsmöglichkeit	Eine Stoß- bzw. Querlüftung oder der Betrieb vorhandener raumlufttechnischer Anlagen ist möglich …<br><br>**… in Sportstätten**	/In keinem Fall<br>/Stimme eher nicht zu<br>/Teils/teils<br>/Stimme eher zu<br>/Stimme voll und ganz zu<br>/Keine Antwort	eigenes Item	-	-	-	positiv	-	-	-
34	ABC1f	Stoß- / Querlüftungsmöglichkeit	Eine Stoß- bzw. Querlüftung oder der Betrieb vorhandener raumlufttechnischer Anlagen ist möglich …<br><br>**… in Mensa / Speisesaal**	/In keinem Fall<br>/Stimme eher nicht zu<br>/Teils/teils<br>/Stimme eher zu<br>/Stimme voll und ganz zu<br>/Keine Antwort	eigenes Item	-	-	-	positiv	-	-	-
35	ABC1g	Stoß- / Querlüftungsmöglichkeit	Eine Stoß- bzw. Querlüftung oder der Betrieb vorhandener raumlufttechnischer Anlagen ist möglich …<br><br>**… in sonstigen Bereichen [Freitext]**	/In keinem Fall<br>/Stimme eher nicht zu<br>/Teils/teils<br>/Stimme eher zu<br>/Stimme voll und ganz zu<br>/Keine Antwort	eigenes Item	-	-	-	positiv	-	-	-
36	ABC2	Infektionsschutzgerechtes Lüften	Die Vorgaben zum infektionsschutzgerechten Lüften werden umgesetzt mit Hilfe von…<br>(Mehrfachnennung möglich)	/… raumlufttechnischen Anlagen<br>/… Fenstern mit Dauerlüften<br>/… Fenstern mit Stoßlüften<br>/… sonstigen Maßnahmen	eigenes Item	-	-	-				
37	ABC3a	Einhaltung des Mindestabstands	Es ist möglich den vorgesehenen Mindestabstand einzuhalten…<br><br>**… in Klassenräumen**	/In keinem Fall<br>/Stimme eher nicht zu<br>/Teils/teils<br>/Stimme eher zu<br>/Stimme voll und ganz zu<br>/Keine Antwort	eigenes Item	-	-	-	positiv	-	-	-
38	ABC3b	Einhaltung des Mindestabstands	Es ist möglich den vorgesehenen Mindestabstand einzuhalten…<br><br>**… auf Fluren**	/In keinem Fall<br>/Stimme eher nicht zu<br>/Teils/teils<br>/Stimme eher zu<br>/Stimme voll und ganz zu<br>/Keine Antwort	eigenes Item	-	-	-	positiv	-	-	-
39	ABC3c	Einhaltung des Mindestabstands	Es ist möglich den vorgesehenen Mindestabstand einzuhalten…<br><br>**… im Lehrerzimmer**	/In keinem Fall<br>/Stimme eher nicht zu<br>/Teils/teils<br>/Stimme eher zu<br>/Stimme voll und ganz zu<br>/Keine Antwort	eigenes Item	-	-	-	positiv	-	-	-
40	ABC3d	Einhaltung des Mindestabstands	Es ist möglich den vorgesehenen Mindestabstand einzuhalten…<br><br>**… in sanitären Einrichtungen**	/In keinem Fall<br>/Stimme eher nicht zu<br>/Teils/teils<br>/Stimme eher zu<br>/Stimme voll und ganz zu<br>/Keine Antwort	eigenes Item	-	-	-	positiv	-	-	-
41	ABC3e	Einhaltung des Mindestabstands	Es ist möglich den vorgesehenen Mindestabstand einzuhalten…<br><br>**… auf dem Schulhof**	/In keinem Fall<br>/Stimme eher nicht zu<br>/Teils/teils<br>/Stimme eher zu<br>/Stimme voll und ganz zu<br>/Keine Antwort	eigenes Item	-	-	-	positiv	-	-	-
42	ABC3f	Einhaltung des Mindestabstands	Es ist möglich den vorgesehenen Mindestabstand einzuhalten…<br><br>**… in der Mensa / im Speisesaal**	/In keinem Fall<br>/Stimme eher nicht zu<br>/Teils/teils<br>/Stimme eher zu<br>/Stimme voll und ganz zu<br>/Keine Antwort	eigenes Item	-	-	-	positiv	-	-	-
43	ABC3g	Einhaltung des Mindestabstands	Es ist möglich den vorgesehenen Mindestabstand einzuhalten…<br><br>**… in Sportstätten**	/In keinem Fall<br>/Stimme eher nicht zu<br>/Teils/teils<br>/Stimme eher zu<br>/Stimme voll und ganz zu<br>/Keine Antwort	eigenes Item	-	-	-	positiv	-	-	-
44	ABC3h	Einhaltung des Mindestabstands	Es ist möglich den vorgesehenen Mindestabstand einzuhalten…<br><br>**… auf dem Schulweg**	/In keinem Fall<br>/Stimme eher nicht zu<br>/Teils/teils<br>/Stimme eher zu<br>/Stimme voll und ganz zu<br>/Keine Antwort	eigenes Item	-	-	-	positiv	-	-	-
45	ABC3i	Einhaltung des Mindestabstands	Es ist möglich den vorgesehenen Mindestabstand einzuhalten…<br><br>**… in sonstigen Bereichen [Freitext]**	/In keinem Fall<br>/Stimme eher nicht zu<br>/Teils/teils<br>/Stimme eher zu<br>/Stimme voll und ganz zu<br>/Keine Antwort	eigenes Item	-	-	-	positiv	-	-	-
46	ABC40	Maskenart	Welche Art von Maske tragen Sie im Schulalltag?<br>(Mehrfachnennung möglich)	/Alltagsmaske (aus Stoff)<br>/Einweg-OP-Maske<br>/FFP2-Maske<br>/FFP3-Maske	eigenes Item	-	-	-	positiv			

47	ABC4a	Einhaltung des Tragens einer Alltagsmaske	Das korrekte Tragen einer Mund-Nase-Bedeckung (über Mund und Nase) wird umgesetzt ...<br>**… in Klassenräumen (außerhalb des Unterrichts)**	/In keinem Fall<br>/Stimme eher nicht zu<br>/Teils/teils<br>/Stimme eher zu<br>/Stimme voll und ganz zu<br>/Keine Antwort	eigenes Item	-	-	-	positiv	-	-	-
48	ABC4b	Einhaltung des Tragens einer Alltagsmaske	Das korrekte Tragen einer Mund-Nase-Bedeckung (über Mund und Nase) wird umgesetzt ...<br>**… in Klassenräumen (während des Unterrichts)**	/In keinem Fall<br>/Stimme eher nicht zu<br>/Teils/teils<br>/Stimme eher zu<br>/Stimme voll und ganz zu<br>/Keine Antwort	eigenes Item	-	-	-	positiv	-	-	-
49	ABC4c	Einhaltung des Tragens einer Alltagsmaske	Das korrekte Tragen einer Mund-Nase-Bedeckung (über Mund und Nase) wird umgesetzt ...<br>**… auf Fluren**	/In keinem Fall<br>/Stimme eher nicht zu<br>/Teils/teils<br>/Stimme eher zu<br>/Stimme voll und ganz zu<br>/Keine Antwort	eigenes Item	-	-	-	positiv	-	-	-
50	ABC4d	Einhaltung des Tragens einer Alltagsmaske	Das korrekte Tragen einer Mund-Nase-Bedeckung (über Mund und Nase) wird umgesetzt ...<br>**… im Lehrerzimmer**	/In keinem Fall<br>/Stimme eher nicht zu<br>/Teils/teils<br>/Stimme eher zu<br>/Stimme voll und ganz zu<br>/Keine Antwort	eigenes Item	-	-	-	positiv	-	-	-
51	ABC4e	Einhaltung des Tragens einer Alltagsmaske	Das korrekte Tragen einer Mund-Nase-Bedeckung (über Mund und Nase) wird umgesetzt ...<br>**… in sanitären Einrichtungen**	/In keinem Fall<br>/Stimme eher nicht zu<br>/Teils/teils<br>/Stimme eher zu<br>/Stimme voll und ganz zu<br>/Keine Antwort	eigenes Item	-	-	-	positiv	-	-	-
52	ABC4f	Einhaltung des Tragens einer Alltagsmaske	Das korrekte Tragen einer Mund-Nase-Bedeckung (über Mund und Nase) wird umgesetzt ...<br>**… auf dem Schulhof**	/In keinem Fall<br>/Stimme eher nicht zu<br>/Teils/teils<br>/Stimme eher zu<br>/Stimme voll und ganz zu<br>/Keine Antwort	eigenes Item	-	-	-	positiv	-	-	-
53	ABC4g	Einhaltung des Tragens einer Alltagsmaske	Das korrekte Tragen einer Mund-Nase-Bedeckung (über Mund und Nase) wird umgesetzt ...<br>**… in der Mensa / im Speisesaal**	/In keinem Fall<br>/Stimme eher nicht zu<br>/Teils/teils<br>/Stimme eher zu<br>/Stimme voll und ganz zu<br>/Keine Antwort	eigenes Item	-	-	-	positiv	-	-	-
54	ABC4h	Einhaltung des Tragens einer Alltagsmaske	Das korrekte Tragen einer Mund-Nase-Bedeckung (über Mund und Nase) wird umgesetzt ...<br>**… in Sportstätten**	/In keinem Fall<br>/Stimme eher nicht zu<br>/Teils/teils<br>/Stimme eher zu<br>/Stimme voll und ganz zu<br>/Keine Antwort	eigenes Item	-	-	-	positiv	-	-	-
55	ABC4i	Einhaltung des Tragens einer Alltagsmaske	Das korrekte Tragen einer Mund-Nase-Bedeckung (über Mund und Nase) wird umgesetzt ...<br>**… auf dem Schulweg**	/In keinem Fall<br>/Stimme eher nicht zu<br>/Teils/teils<br>/Stimme eher zu<br>/Stimme voll und ganz zu<br>/Keine Antwort	eigenes Item	-	-	-	positiv	-	-	-
56	ABC4j	Einhaltung des Tragens einer Alltagsmaske	Das korrekte Tragen einer Mund-Nase-Bedeckung (über Mund und Nase) wird umgesetzt ...<br>**… in sonstigen Bereichen [Freitext]**	/In keinem Fall<br>/Stimme eher nicht zu<br>/Teils/teils<br>/Stimme eher zu<br>/Stimme voll und ganz zu<br>/Keine Antwort	eigenes Item	-	-	-	positiv	-	-	-
57	ABC5	Möglichkeit Händereinigung	An Ihrer Schule / Dienststelle ist es möglich eine hygienische Händereinigung durchzuführen (Händewaschen mit Seife und Einmalhandtüchern oder Händedesinfektion).	/In keinem Fall<br>/Stimme eher nicht zu<br>/Teils/teils<br>/Stimme eher zu<br>/Stimme voll und ganz zu<br>/Keine Antwort	eigenes Item	-	-	-	positiv	-	-	-

4. Auswirkungen des Schulbetriebs während der COVID-19-Pandemie auf Lehrkräfte

4a) Arbeitsbezogene Auswirkungen

1	AZ1	Arbeitszufriedenheit global	Wie zufrieden sind Sie mit Ihrer beruflichen Situation insgesamt?	/überhaupt nicht<br>/wenig<br>/ziemlich<br>/sehr stark<br>/extrem	Aus Fragebogen IfL	Arbeitszufriedenheit	-	-	positiv	-	-	-
2	AZ1a	Delta zu vor COVID-19-Pandemie	Wie würden Sie diesen Aspekt im Vergleich zu vor der COVID-19-Pandemie beschreiben?	/Derzeit viel besser als vor der COVID-19-Pandemie<br>/Derzeit etwas besser als vor der COVID-19-Pandemie<br>/Etwa so wie vor der COVID-19-Pandemie<br>/Derzeit etwas schlechter als vor der COVID-19-Pandemie<br>/Derzeit viel schlechter als vor der COVID-19-Pandemie	eigenes Item		-	-	-	-	-	-
3	AZ2	Zeitliche Anforderungen	Wie oft kommt es vor, dass Sie nicht genügend Zeit haben, alle Ihre Aufgaben zu erledigen?	/immer<br>/oft<br>/manchmal<br>/selten<br>/nie / fast nie	COPSOQ 2020	Quantitative Anforderungen	ja	nein	positiv	https://www.copsoq.de/copsoq-fragebogen/	Ramkissoon (2019) - Dissecting the effect of workplace exposures on workers'rating of psychological health and safety	Bethelsen et al. (2020) - Validation of the Copenhagen Psychosocial Questionnaire Version III
4	AZ2a	Delta zu vor COVID-19-Pandemie	Wie würden Sie diesen Aspekt im Vergleich zu vor der COVID-19-Pandemie beschreiben?	/Derzeit viel häufiger als vor der COVID-19-Pandemie<br>/Derzeit etwas häufiger als vor der COVID-19-Pandemie<br>/Etwa so häufig wie vor der COVID-19-Pandemie<br>/Derzeit etwas seltener als vor der COVID-19-Pandemie<br>/Derzeit viel seltener als vor der COVID-19-Pandemie	eigenes Item		-	-	-	-	-	-

5	SI1	Sinnerleben bei der Arbeit	Ist Ihre Arbeit sinnvoll?	/in sehr hohem Maße<br>/in hohem Maße<br>/zum Teil<br>/in geringem Maße<br>/in sehr gereingem Maß	COPSOQ 2020	Bedeutung der Arbeit	ja	nein	positiv	https://www.copsoq.de/copsoq-fragebogen/	Ramkissoon (2019) - Dissecting the effect of workplace exposures on workers’rating of psychological health and safety	Bethelsen et al. (2020) - Validation of the Copenhagen Psychosocial Questionnaire Version III
6	SI1a	Delta zu vor COVID-19-Pandemie	Wie würden Sie diesen Aspekt im Vergleich zu vor der COVID-19-Pandemie beschreiben?	/Derzeit viel mehr als vor der COVID-19-Pandemie<br>/Derzeit etwas mehr als vor der COVID-19-Pandemie<br>/Etwa so wie vor der COVID-19-Pandemie<br>/Derzeit etwas weniger als vor der COVID-19-Pandemie<br>/Derzeit viel weniger als vor der COVID-19-Pandemie	eigenes Item		-	-	-	-	-	-
7	PR1	Vorhersehbarkeit der Arbeit	Werden Sie rechtzeitig im Voraus über Veränderungen an Ihrem Arbeitsplatz informiert, z.B. über wichtige Entscheidungen, Veränderungen oder Pläne für die Zukunft?	/in sehr hohem Maße<br>/in hohem Maße<br>/zum Teil<br>/in geringem Maße<br>/in sehr gereingem Maß	COPSOQ 2020		ja	ja	positiv	https://www.copsoq.de/copsoq-fragebogen/	Ramkissoon (2019) - Dissecting the effect of workplace exposures on workers’rating of psychological health and safety	Bethelsen et al. (2020) - Validation of the Copenhagen Psychosocial Questionnaire Version III
8	PR1a	Delta zu vor COVID-19-Pandemie	Wie würden Sie diesen Aspekt im Vergleich zu vor der COVID-19-Pandemie beschreiben?	/Derzeit viel mehr als vor der COVID-19-Pandemie<br>/Derzeit etwas mehr als vor der COVID-19-Pandemie<br>/Etwa so wie vor der COVID-19-Pandemie<br>/Derzeit etwas weniger als vor der COVID-19-Pandemie<br>/Derzeit viel weniger als vor der COVID-19-Pandemie	eigenes Item		-	-	-	-	-	-
9	PR2	Vorhersehbarkeit der Arbeit	Erhalten Sie alle Informationen, die Sie brauchen, um Ihre Arbeit gut zu erledigen?	/in sehr hohem Maße<br>/in hohem Maße<br>/zum Teil<br>/in geringem Maße<br>/in sehr gereingem Maß	COPSOQ 2020	Vorhersehbarkeit der Arbeit	ja	ja	positiv	https://www.copsoq.de/copsoq-fragebogen/	Ramkissoon (2019) - Dissecting the effect of workplace exposures on workers’rating of psychological health and safety	Bethelsen et al. (2020) - Validation of the Copenhagen Psychosocial Questionnaire Version III
10	PR2a	Delta zu vor COVID-19-Pandemie	Wie würden Sie diesen Aspekt im Vergleich zu vor der COVID-19-Pandemie beschreiben?	/Derzeit viel mehr als vor der COVID-19-Pandemie<br>/Derzeit etwas mehr als vor der COVID-19-Pandemie<br>/Etwa so wie vor der COVID-19-Pandemie<br>/Derzeit etwas weniger als vor der COVID-19-Pandemie<br>/Derzeit viel weniger als vor der COVID-19-Pandemie	eigenes Item		-	-	-	-	-	-
11	EIN1	Einfluss auf die Arbeit	Haben Sie großen Einfluss auf Entscheidungen, die Ihre Arbeit betreffen?	/immer<br>/oft<br>/manchmal<br>/selten<br>/nie / fast nie	COPSOQ 2020		ja	ja	positiv	https://www.copsoq.de/copsoq-fragebogen/	Ramkissoon (2019) - Dissecting the effect of workplace exposures on workers’rating of psychological health and safety	Bethelsen et al. (2020) - Validation of the Copenhagen Psychosocial Questionnaire Version III
12	EIN1a	Delta zu vor COVID-19-Pandemie	Wie würden Sie diesen Aspekt im Vergleich zu vor der COVID-19-Pandemie beschreiben?	/Derzeit viel mehr als vor der COVID-19-Pandemie<br>/Derzeit etwas mehr als vor der COVID-19-Pandemie<br>/Etwa so wie vor der COVID-19-Pandemie<br>/Derzeit etwas weniger als vor der COVID-19-Pandemie<br>/Derzeit viel weniger als vor der COVID-19-Pandemie	eigenes Item		-	-	-	-	-	-
13	EIN2	Einfluss auf die Arbeit	Haben Sie Einfluss auf die Menge der Arbeit, die Ihnen übertragen wird?	/immer<br>/oft<br>/manchmal<br>/selten<br>/nie / fast nie	COPSOQ 2020	Einfluss auf die Arbeit	ja	ja	positiv	https://www.copsoq.de/copsoq-fragebogen/	Ramkissoon (2019) - Dissecting the effect of workplace exposures on workers’rating of psychological health and safety	Bethelsen et al. (2020) - Validation of the Copenhagen Psychosocial Questionnaire Version III
14	EIN2a	Delta zu vor COVID-19-Pandemie	Wie würden Sie diesen Aspekt im Vergleich zu vor der COVID-19-Pandemie beschreiben?	/Derzeit viel mehr als vor der COVID-19-Pandemie<br>/Derzeit etwas mehr als vor der COVID-19-Pandemie<br>/Etwa so wie vor der COVID-19-Pandemie<br>/Derzeit etwas weniger als vor der COVID-19-Pandemie<br>/Derzeit viel weniger als vor der COVID-19-Pandemie	eigenes Item		-	-	-	-	-	-
15	EIN3	Einfluss auf die Arbeit	Haben Sie Einfluss darauf, was Sie bei Ihrer Arbeit tun?	/immer<br>/oft<br>/manchmal<br>/selten<br>/nie / fast nie	COPSOQ 2020		ja	ja	positiv	https://www.copsoq.de/copsoq-fragebogen/	Ramkissoon (2019) - Dissecting the effect of workplace exposures on workers’rating of psychological health and safety	Bethelsen et al. (2020) - Validation of the Copenhagen Psychosocial Questionnaire Version III
16	EIN3a	Delta zu vor COVID-19-Pandemie	Wie würden Sie diesen Aspekt im Vergleich zu vor der COVID-19-Pandemie beschreiben?	/Derzeit viel mehr als vor der COVID-19-Pandemie<br>/Derzeit etwas mehr als vor der COVID-19-Pandemie<br>/Etwa so wie vor der COVID-19-Pandemie<br>/Derzeit etwas weniger als vor der COVID-19-Pandemie<br>/Derzeit viel weniger als vor der COVID-19-Pandemie	eigenes Item		-	-	-	-	-	-
17	EMO1	Emotionale Anforderungen	Gehört es zu Ihrer Arbeit, sich mit den persönlichen Problemen anderer Menschen zu beschäftigen?	/immer<br>/oft<br>/manchmal<br>/selten<br>/nie / fast nie	COPSOQ 2020		ja	ja	negativ	https://www.copsoq.de/copsoq-fragebogen/	Ramkissoon (2019) - Dissecting the effect of workplace exposures on workers’rating of psychological health and safety	Bethelsen et al. (2020) - Validation of the Copenhagen Psychosocial Questionnaire Version III
18	EMO1a	Delta zu vor COVID-19-Pandemie	Wie würden Sie diesen Aspekt im Vergleich zu vor der COVID-19-Pandemie beschreiben?	/Derzeit viel mehr als vor der COVID-19-Pandemie<br>/Derzeit etwas mehr als vor der COVID-19-Pandemie<br>/Etwa so wie vor der COVID-19-Pandemie<br>/Derzeit etwas weniger als vor der COVID-19-Pandemie<br>/Derzeit viel weniger als vor der COVID-19-Pandemie	eigenes Item	Emotionale Anforderungen	-	-	-	-	-	-

19	EMO2	Emotionale Anforderungen	Ist Ihre Arbeit emotional fordernd?	/In sehr hohem Maße<br>/In hohem Maße<br>/Zum Teil<br>/In geringem Maße<br>/In sehr geringem Maße	COPSOQ 2020	Emotionale Anforderungen	ja	ja	negativ	https://www.copsoq.de/copsoq-fragebogen/	Ramkissoon (2019) - Dissecting the effect of workplace exposures on workers'rating of psychological health and safety	Bethelsen et al. (2020) - Validation of the Copenhagen Psychosocial Questionnaire Version III
20	EMO2a	Delta zu vor COVID-19-Pandemie	Wie würden Sie diesen Aspekt im Vergleich zu vor der COVID-19-Pandemie beschreiben?	/Derzeit viel mehr als vor der COVID-19-Pandemie<br>/Derzeit etwas mehr als vor der COVID-19-Pandemie<br>/Etwa so wie vor der COVID-19-Pandemie<br>/Derzeit etwas weniger als vor der COVID-19-Pandemie<br>/Derzeit viel weniger als vor der COVID-19-Pandemie	eigenes Item		-	-	-	-	-	-
21	VER1	Verbergen von Emotionen	Verlangt Ihre Arbeit von Ihnen, dass Sie Ihre Gefühle verbergen?	/In sehr hohem Maße<br>/In hohem Maße<br>/Zum Teil<br>/In geringem Maße<br>/In sehr geringem Maße	COPSOQ 2020		ja	ja	negativ	https://www.copsoq.de/copsoq-fragebogen/	Ramkissoon (2019) - Dissecting the effect of workplace exposures on workers'rating of psychological health and safety	Bethelsen et al. (2020) - Validation of the Copenhagen Psychosocial Questionnaire Version III
22	VER1a	Delta zu vor COVID-19-Pandemie	Wie würden Sie diesen Aspekt im Vergleich zu vor der COVID-19-Pandemie beschreiben?	/Derzeit viel mehr als vor der COVID-19-Pandemie<br>/Derzeit etwas mehr als vor der COVID-19-Pandemie<br>/Etwa so wie vor der COVID-19-Pandemie<br>/Derzeit etwas weniger als vor der COVID-19-Pandemie<br>/Derzeit viel weniger als vor der COVID-19-Pandemie	eigenes Item		-	-	-	-	-	-
23	VER2	Verbergen von Emotionen	Verlangt Ihre Arbeit von Ihnen, sich mit Ihrer Meinung zurück zu halten?	/In sehr hohem Maße<br>/In hohem Maße<br>/Zum Teil<br>/In geringem Maße<br>/In sehr geringem Maße	COPSOQ 2020	Verbergen von Emotionen	ja	ja	negativ	https://www.copsoq.de/copsoq-fragebogen/	Ramkissoon (2019) - Dissecting the effect of workplace exposures on workers'rating of psychological health and safety	Bethelsen et al. (2020) - Validation of the Copenhagen Psychosocial Questionnaire Version III
24	VER2a	Delta zu vor COVID-19-Pandemie	Wie würden Sie diesen Aspekt im Vergleich zu vor der COVID-19-Pandemie beschreiben?	/Derzeit viel mehr als vor der COVID-19-Pandemie<br>/Derzeit etwas mehr als vor der COVID-19-Pandemie<br>/Etwa so wie vor der COVID-19-Pandemie<br>/Derzeit etwas weniger als vor der COVID-19-Pandemie<br>/Derzeit viel weniger als vor der COVID-19-Pandemie	eigenes Item		-	-	-	-	-	-
25	WPK3	Work-Privacy-Konflikte	Meine Arbeit beansprucht so viel Energie, dass sich dies negativ auf mein Privatleben auswirkt.	/In sehr hohem Maße<br>/In hohem Maße<br>/Zum Teil<br>/In geringem Maße<br>/In sehr geringem Maße	COPSOQ 2020		ja	ja	negativ	https://www.copsoq.de/copsoq-fragebogen/	Ramkissoon (2019) - Dissecting the effect of workplace exposures on workers'rating of psychological health and safety	Bethelsen et al. (2020) - Validation of the Copenhagen Psychosocial Questionnaire Version III
26	WPK3a	Delta zu vor COVID-19-Pandemie	Wie würden Sie diesen Aspekt im Vergleich zu vor der COVID-19-Pandemie beschreiben?	/Derzeit viel mehr als vor der COVID-19-Pandemie<br>/Derzeit etwas mehr als vor der COVID-19-Pandemie<br>/Etwa so wie vor der COVID-19-Pandemie<br>/Derzeit etwas weniger als vor der COVID-19-Pandemie<br>/Derzeit viel weniger als vor der COVID-19-Pandemie	eigenes Item	Work-Privacy Konflikt (2 Item-Version core-COPSOQ)	-	-	-	-	-	-
27	WPK4	Work-Privacy-Konflikte	Meine Arbeit nimmt so viel Zeit in Anspruch, dass sich dies negativ auf mein Privatleben auswirkt	/In sehr hohem Maße<br>/In hohem Maße<br>/Zum Teil<br>/In geringem Maße<br>/In sehr geringem Maße	COPSOQ 2020		ja	ja	negativ	https://www.copsoq.de/copsoq-fragebogen/	Ramkissoon (2019) - Dissecting the effect of workplace exposures on workers'rating of psychological health and safety	Bethelsen et al. (2020) - Validation of the Copenhagen Psychosocial Questionnaire Version III
28	WPK4a	Delta zu vor COVID-19-Pandemie	Wie würden Sie diesen Aspekt im Vergleich zu vor der COVID-19-Pandemie beschreiben?	/Derzeit viel mehr als vor der COVID-19-Pandemie<br>/Derzeit etwas mehr als vor der COVID-19-Pandemie<br>/Etwa so wie vor der COVID-19-Pandemie<br>/Derzeit etwas weniger als vor der COVID-19-Pandemie<br>/Derzeit viel weniger als vor der COVID-19-Pandemie	eigenes Item		-	-	-	-	-	-
29	UNT1	Unterstützung bei der Arbeit - Kollegen	Wie oft erhalten Sie bei Bedarf Hilfe und Unterstützung von Ihren Kollegen/innen?	/immer<br>/oft<br>/manchmal<br>/selten<br>/nie / fast nie	COPSOQ 2020	Unterstützung bei der Arbeit - Kollegen	ja	ja	positiv	https://www.copsoq.de/copsoq-fragebogen/	Ramkissoon (2019) - Dissecting the effect of workplace exposures on workers'rating of psychological health and safety	Bethelsen et al. (2020) - Validation of the Copenhagen Psychosocial Questionnaire Version III
30	UNT1a	Delta zu vor COVID-19-Pandemie	Wie würden Sie diesen Aspekt im Vergleich zu vor der COVID-19-Pandemie beschreiben?	/Derzeit viel häufiger als vor der COVID-19-Pandemie<br>/Derzeit etwas häufiger als vor der COVID-19-Pandemie<br>/Etwa so häufig wie vor der COVID-19-Pandemie<br>/Derzeit etwas seltener als vor der COVID-19-Pandemie<br>/Derzeit viel seltener als vor der COVID-19-Pandemie	eigenes Item		-	-	-	-	-	-
31	UNT2	Unterstützung bei der Arbeit - Vorgesetzte	Wie oft erhalten Sie bei Bedarf Hilfe und Unterstützung von Ihrem / Ihrer unmittelbaren Vorgesetzten?	/immer<br>/oft<br>/manchmal<br>/selten<br>/nie / fast nie	COPSOQ 2020	Unterstützung bei der Arbeit - Vorgesetzte	ja	ja	positiv	https://www.copsoq.de/copsoq-fragebogen/	Ramkissoon (2019) - Dissecting the effect of workplace exposures on workers'rating of psychological health and safety	Bethelsen et al. (2020) - Validation of the Copenhagen Psychosocial Questionnaire Version III
32	UNT2a	Delta zu vor COVID-19-Pandemie	Wie würden Sie diesen Aspekt im Vergleich zu vor der COVID-19-Pandemie beschreiben?	/Derzeit viel häufiger als vor der COVID-19-Pandemie<br>/Derzeit etwas häufiger als vor der COVID-19-Pandemie<br>/Etwa so häufig wie vor der COVID-19-Pandemie<br>/Derzeit etwas seltener als vor der COVID-19-Pandemie<br>/Derzeit viel seltener als vor der COVID-19-Pandemie	eigenes Item		-	-	-	-	-	-

33	FB1	Feedback / Rückmeldung	Wie oft spricht Ihr/e Vorgesetzte/r mit Ihnen über die Qualität Ihrer Arbeit?	/immer<br>/oft<br>/manchmal<br>/selten<br>/nie / fast nie	COPSOQ 2020		ja	ja	positiv	https://www.copsoq.de/copsoq-fragebogen/	Ramkissoon (2019) - Dissecting the effect of workplace exposures on workers'rating of psychological health and safety	Bethelsen et al. (2020) - Validation of the Copenhagen Psychosocial Questionnaire Version III
34	FB1a	Delta zu vor COVID-19-Pandemie	Wie würden Sie diesen Aspekt im Vergleich zu vor der COVID-19-Pandemie beschreiben?	/Derzeit viel häufiger als vor der COVID-19-Pandemie<br>/Derzeit etwas häufiger als vor der COVID-19-Pandemie<br>/Etwa so häufig wie vor der COVID-19-Pandemie<br>/Derzeit etwas seltener als vor der COVID-19-Pandemie<br>/Derzeit viel seltener als vor der COVID-19-Pandemie	eigenes Item		-	-	-	-	-	-
35	FB2	Feedback / Rückmeldung	Wie oft sprechen Ihre Kollegen/innen mit Ihnen über die Qualität Ihrer Arbeit?	/immer<br>/oft<br>/manchmal<br>/selten<br>/nie / fast nie	COPSOQ 2020	Feedback / Rückmeldung	ja	ja	positiv	https://www.copsoq.de/copsoq-fragebogen/	Ramkissoon (2019) - Dissecting the effect of workplace exposures on workers'rating of psychological health and safety	Bethelsen et al. (2020) - Validation of the Copenhagen Psychosocial Questionnaire Version III
36	FB2a	Delta zu vor COVID-19-Pandemie	Wie würden Sie diesen Aspekt im Vergleich zu vor der COVID-19-Pandemie beschreiben?	/Derzeit viel häufiger als vor der COVID-19-Pandemie<br>/Derzeit etwas häufiger als vor der COVID-19-Pandemie<br>/Etwa so häufig wie vor der COVID-19-Pandemie<br>/Derzeit etwas seltener als vor der COVID-19-Pandemie<br>/Derzeit viel seltener als vor der COVID-19-Pandemie	eigenes Item		-	-	-	-	-	-
37	UNG	Ungerechte Behandlung	Wie oft fühlen Sie sich durch Kollegen/innen oder Vorgesetzte zu Unrecht kritisiert, schikaniert oder vor anderen bloßgestellt?	/immer<br>/oft<br>/manchmal<br>/selten<br>/nie / fast nie	COPSOQ 2020	Ungerechte Behandlung	ja	ja	negativ	https://www.copsoq.de/copsoq-fragebogen/	Ramkissoon (2019) - Dissecting the effect of workplace exposures on workers'rating of psychological health and safety	Bethelsen et al. (2020) - Validation of the Copenhagen Psychosocial Questionnaire Version III
38	UNGa	Delta zu vor COVID-19-Pandemie	Wie würden Sie diesen Aspekt im Vergleich zu vor der COVID-19-Pandemie beschreiben?	/Derzeit viel häufiger als vor der COVID-19-Pandemie<br>/Derzeit etwas häufiger als vor der COVID-19-Pandemie<br>/Etwa so häufig wie vor der COVID-19-Pandemie<br>/Derzeit etwas seltener als vor der COVID-19-Pandemie<br>/Derzeit viel seltener als vor der COVID-19-Pandemie	eigenes Item		-	-	-	-	-	-
39	IFL1	Arbeitsbelastung	Bitte überprüfen Sie, wie sehr Sie das folgende Problem während der letzten Woche belastet hat.<br>**Wie stark fühlen Sie sich durch Ihre berufliche Situation belastet?**	/überhaupt nicht<br>/wenig<br>/ziemlich<br>/sehr stark<br>/extrem	IfL-Gesundheitsfragebogen	-	ja	ja	negativ			
40	IFL1a	Delta zu vor COVID-19-Pandemie	Wie würden Sie diesen Aspekt im Vergleich zu vor der COVID-19-Pandemie beschreiben?	/Derzeit viel mehr als vor der COVID-19-Pandemie<br>/Derzeit etwas mehr als vor der COVID-19-Pandemie<br>/Etwa so wie vor der COVID-19-Pandemie<br>/Derzeit etwas weniger als vor der COVID-19-Pandemie<br>/Derzeit viel weniger als vor der COVID-19-Pandemie	eigenes Item	-	-	-	-	-	-	-
41	IFL2	Ungerechte Behandlung	Im Berufsalltag habe ich oftmals das Gefühl, dass ich ungerecht behandelt werde.	/Starke Ablehnung<br>/Ablehnung<br>/Leichte Ablehnung<br>/Leichte Zustimmung<br>/Zustimmung<br>/Starke Zustimmung	IfL-Gesundheitsfragebogen	-						
42	IFL2a	Delta zu vor COVID-19-Pandemie	Wie würden Sie diesen Aspekt im Vergleich zu vor der COVID-19-Pandemie beschreiben?	/Derzeit viel mehr als vor der COVID-19-Pandemie<br>/Derzeit etwas mehr als vor der COVID-19-Pandemie<br>/Etwa so wie vor der COVID-19-Pandemie<br>/Derzeit etwas weniger als vor der COVID-19-Pandemie<br>/Derzeit viel weniger als vor der COVID-19-Pandemie	eigenes Item	-	-	-	-	-	-	-
43	IFL3	Gesundheit bis Pensionsalter	Wenn Sie an Ihren Gesundheitszustand und Ihre berufliche Leistungsfähigkeit denken: Glauben Sie, dass Sie bis zum Erreichen des Pensionsalters berufstätig sein können?	/Sicher<br>/Eher ja<br>/Unsicher<br>/Eher nein<br>/Auf keinen Fall	IfL-Gesundheitsfragebogen	-						
44	IFL3a	Delta zu vor COVID-19-Pandemie	Wie würden Sie diesen Aspekt im Vergleich zu vor der COVID-19-Pandemie beschreiben?	/Derzeit viel mehr als vor der COVID-19-Pandemie<br>/Derzeit etwas mehr als vor der COVID-19-Pandemie<br>/Etwa so wie vor der COVID-19-Pandemie<br>/Derzeit etwas weniger als vor der COVID-19-Pandemie<br>/Derzeit viel weniger als vor der COVID-19-Pandemie	eigenes Item	-	-	-	-	-	-	-
4b) Beanspruchungen (somatisch / psychisch) sowie private / soziale Herausforderungen												
45	WHO1	WHO-self rated general health	Wie würden Sie Ihren Gesundheitszustand im Allgemeinen beschreiben?	/sehr schlecht<br>/schlecht<br>/nicht ganz zufriedenstellend<br>/zufriedenstellend<br>/gut<br>/sehr gut	WHO	self-rated health	ja	ja	positiv	Übersetzt aus: Self-rated health as a valid indicator for health-equity analyses: evidence from the Italian health interview survey	Self-rated health as a valid indicator for health-equity analyses: evidence from the Italian health interview survey	-
46	WHO1a	Delta zu vor COVID-19-Pandemie	Wie würden Sie diesen Aspekt im Vergleich zu vor der COVID-19-Pandemie beschreiben?	/Derzeit viel besser als vor der COVID-19-Pandemie<br>/Derzeit etwas besser als vor der COVID-19-Pandemie<br>/Etwa so wie vor der COVID-19-Pandemie<br>/Derzeit etwas schlechter als vor der COVID-19-Pandemie<br>/Derzeit viel schlechter als vor der COVID-19-Pandemie	eigenes Item	-	-	-	-	-	-	-
47	COV1	Covid-19 persönliche Betroffenheit	Mussten Sie sich aufgrund eines Verdachtes auf COVID-19 (z. B. Symptome, Kontakt zu Personen mit bestätigter Erkrankung, Rückkehr aus Risikogebiet) in häusliche Quarantäne begeben?	/Ja<br>/Nein	Angelehnt an Befragung von Prof. Witthöft	-	-	-	-	-	-	-
48	COV2	Covid-19 persönliche Betroffenheit	Sind Sie aktuell oder waren Sie in der Vergangenheit bereits mit dem SARS-CoV-2-Virus infiziert (COVID-19)?	/Ja<br>/Nein	Angelehnt an Befragung von Prof. Witthöft	-	-	-	-	-	-	-

49	COV2a	Covid-19 persönliche Betroffenheit	(Filterfrage: falls COV2 = ja)<br>Wie schwer war der Verlauf ihrer COVID-19-Erkrankung?	/Keine Symptome<br>/Leicht (keine ärztliche Betreuung erforderlich)<br>/Mittelschwer (z. B. Husten, Beeinträchtigung des Geruchssinns / hausärztliche Betreuung in Anspruch genommen)<br>/Schwer (z. B. Atemnot, starke Schmerzen, / stationäre Behandlung in Krankenhaus notwendig)<br>/Sehr schwer (Intensivmedizinische Behandlung notwendig)	eigenes Item	-	-	-	-	-	-	-
50	COV3	Covid-19 persönliche Betroffenheit	Ist aktuell oder war in der Vergangenheit eine Ihnen nahestehende Person mit dem SARS-CoV-2-Virus infiziert (COVID-19)?	/Ja<br>/Nein	eigenes Item	-	-	-	-	-	-	-
51	P1	Somatische Beschwerden in den letzten 4 Wochen	Wie stark fühlten Sie sich im Verlauf der letzten 4 Wochen durch die folgenden Beschwerden beeinträchtigt?<br>**Bauchschmerzen**	/Nicht beeinträchtigt<br>/Wenig beeinträchtigt<br>/Stark beeinträchtigt	PHQ-15		ja	ja	negativ			
52	P2	Somatische Beschwerden in den letzten 4 Wochen	Wie stark fühlten Sie sich im Verlauf der letzten 4 Wochen durch die folgenden Beschwerden beeinträchtigt?<br>**Rückenschmerzen**	/Nicht beeinträchtigt<br>/Wenig beeinträchtigt<br>/Stark beeinträchtigt	PHQ-15		ja	ja	negativ			
53	P3	Somatische Beschwerden in den letzten 4 Wochen	Wie stark fühlten Sie sich im Verlauf der letzten 4 Wochen durch die folgenden Beschwerden beeinträchtigt?<br>**Schmerzen in Armen, Beinen oder Gelenken (Knie, Hüften usw.)**	/Nicht beeinträchtigt<br>/Wenig beeinträchtigt<br>/Stark beeinträchtigt	PHQ-15		ja	ja	negativ			
54	P4	Somatische Beschwerden in den letzten 4 Wochen	Wie stark fühlten Sie sich im Verlauf der letzten 4 Wochen durch die folgenden Beschwerden beeinträchtigt?<br>**Menstruationsschmerzen oder andere Probleme mit der Menstruation**	/Nicht beeinträchtigt<br>/Wenig beeinträchtigt<br>/Stark beeinträchtigt	PHQ-15		ja	ja	negativ			
55	P5	Somatische Beschwerden in den letzten 4 Wochen	Wie stark fühlten Sie sich im Verlauf der letzten 4 Wochen durch die folgenden Beschwerden beeinträchtigt?<br>**Schmerzen oder Probleme beim Geschlechtsverkehr**	/Nicht beeinträchtigt<br>/Wenig beeinträchtigt<br>/Stark beeinträchtigt	PHQ-15		ja	ja	negativ			
56	P6	Somatische Beschwerden in den letzten 4 Wochen	Wie stark fühlten Sie sich im Verlauf der letzten 4 Wochen durch die folgenden Beschwerden beeinträchtigt?<br>**Kopfschmerzen**	/Nicht beeinträchtigt<br>/Wenig beeinträchtigt<br>/Stark beeinträchtigt	PHQ-15		ja	ja	negativ			
57	P7	Somatische Beschwerden in den letzten 4 Wochen	Wie stark fühlten Sie sich im Verlauf der letzten 4 Wochen durch die folgenden Beschwerden beeinträchtigt?<br>**Schmerzen im Brustbein**	/Nicht beeinträchtigt<br>/Wenig beeinträchtigt<br>/Stark beeinträchtigt	PHQ-15	PHQ-15	ja	ja	negativ	Deutsche Übersetzung und Validierung des "Patient Health Questionaire" (PHQ) durch B. Löwe, S. Zipfel und W. Herzog, Universitätsklinikum Hamburg-Eppendorf und Universitätsklinikum Heidelberg (Englische Originalversion: Spitzer, Kroenke Williams, JAMA, 1999)	Kocalevent et al. 2013: Standardization of a screening instrument (PHQ-15) for somatization syndromes in the general population.	Kroenke K, Spitzer RL, Williams JB. The PHQ-15: Validity of a New Measure for Evaluating the Severity of Somatic Symptoms. Psychosom Med 2002;64:258-66.
58	P8	Somatische Beschwerden in den letzten 4 Wochen	Wie stark fühlten Sie sich im Verlauf der letzten 4 Wochen durch die folgenden Beschwerden beeinträchtigt?<br>**Schwindel**	/Nicht beeinträchtigt<br>/Wenig beeinträchtigt<br>/Stark beeinträchtigt	PHQ-15		ja	ja	negativ			
59	P9	Somatische Beschwerden in den letzten 4 Wochen	Wie stark fühlten Sie sich im Verlauf der letzten 4 Wochen durch die folgenden Beschwerden beeinträchtigt?<br>**Ohnmachtsanfälle**	/Nicht beeinträchtigt<br>/Wenig beeinträchtigt<br>/Stark beeinträchtigt	PHQ-15		ja	ja	negativ			
60	P10	Somatische Beschwerden in den letzten 4 Wochen	Wie stark fühlten Sie sich im Verlauf der letzten 4 Wochen durch die folgenden Beschwerden beeinträchtigt?<br>**Herzklopfen oder Herzrasen**	/Nicht beeinträchtigt<br>/Wenig beeinträchtigt<br>/Stark beeinträchtigt	PHQ-15		ja	ja	negativ			
61	P11	Somatische Beschwerden in den letzten 4 Wochen	Wie stark fühlten Sie sich im Verlauf der letzten 4 Wochen durch die folgenden Beschwerden beeinträchtigt?<br>**Kurzatmigkeit**	/Nicht beeinträchtigt<br>/Wenig beeinträchtigt<br>/Stark beeinträchtigt	PHQ-15		ja	ja	negativ			
62	P12	Somatische Beschwerden in den letzten 4 Wochen	Wie stark fühlten Sie sich im Verlauf der letzten 4 Wochen durch die folgenden Beschwerden beeinträchtigt?<br>**Verstopfung, nervöser Darm oder Durchfall**	/Nicht beeinträchtigt<br>/Wenig beeinträchtigt<br>/Stark beeinträchtigt	PHQ-15		ja	ja	negativ			
63	P13	Somatische Beschwerden in den letzten 4 Wochen	Wie stark fühlten Sie sich im Verlauf der letzten 4 Wochen durch die folgenden Beschwerden beeinträchtigt?<br>**Übelkeit, Blähungen oder Verdauungsbeschwerden**	/Nicht beeinträchtigt<br>/Wenig beeinträchtigt<br>/Stark beeinträchtigt	PHQ-15		ja	ja	negativ			
64	P14	Psychische Beschwerden in den letzten 2 Wochen	Wie stark fühlten Sie sich im Verlauf der letzten 2 Wochen durch die folgenden Beschwerden beeinträchtigt?<br>**Schwierigkeiten, ein- oder durchzuschlafen oder vermehrter Schlaf**	/Überhaupt nicht<br>/An einzelnen Tagen<br>/An mehr als der Hälfte der Tage<br>/Beinahe jeden Tag	PHQ-15		ja	ja	negativ			
65	P15	Psychische Beschwerden in den letzten 2 Wochen	Wie stark fühlten Sie sich im Verlauf der letzten 2 Wochen durch die folgenden Beschwerden beeinträchtigt?<br>**Müdigkeit oder Gefühl, keine Energie zu haben**	/Überhaupt nicht<br>/An einzelnen Tagen<br>/An mehr als der Hälfte der Tage<br>/Beinahe jeden Tag	PHQ-15		ja	ja	negativ			
66	RF1	Risikofaktoren persönlich	Welche der aktuell bekannten Risikofaktoren für einen schweren Krankheitsverlauf bei einer Covid-19-Erkrankung treffen auf Sie zu?<br>**Herz-Kreislauf-Erkrankung mit stark eingeschränkter Pumpfunktion des Herzens oder Folgeschäden (z. B. Herzinsuffizienz, koronare Herzkrankheit)**	/Ja<br>/Nein<br>/Keine Antwort	eigenes Item		-	-	negativ	-	-	-
67	RF2	Risikofaktoren persönlich	Welche der aktuell bekannten Risikofaktoren für einen schweren Krankheitsverlauf bei einer Covid-19-Erkrankung treffen auf Sie zu?<br>**Chronische Lungenerkrankung (z. B. COPD mit mittelmäßig bis stark eingeschränkter Lungenfunktion, Mukoviszidose)**	/Ja<br>/Nein<br>/Keine Antwort	eigenes Item		-	-	negativ	-	-	-

68	RF3	Risikofaktoren persönlich	Welche der aktuell bekannten Risikofaktoren für einen schweren Krankheitsverlauf bei einer Covid-19-Erkrankung treffen auf Sie zu?<br>**Schwere Magen-, Darmerkrankungen (z. B. chronisch entzündliche Darmerkrankungen mit Immunsuppression) und / oder fortgeschrittene Leberzirrhosen**	/Ja<br>/Nein<br>/Keine Antwort	eigenes Item	Risikofaktoren schwerer Verlauf COVID-19 - persönlich	-	-	negativ	-	-	-
69	RF4	Risikofaktoren persönlich	Welche der aktuell bekannten Risikofaktoren für einen schweren Krankheitsverlauf bei einer Covid-19-Erkrankung treffen auf Sie zu?<br>**Diabetes mellitus Typ I – insulinpflichtig oder mit Folgeschäden**	/Ja<br>/Nein<br>/Keine Antwort	eigenes Item		-	-	negativ	-	-	-
70	RF5	Risikofaktoren persönlich	Welche der aktuell bekannten Risikofaktoren für einen schweren Krankheitsverlauf bei einer Covid-19-Erkrankung treffen auf Sie zu?<br>**Krebserkrankung unter laufender Therapie oder mit Langzeittherapiefolgen**	/Ja<br>/Nein<br>/Keine Antwort	eigenes Item		-	-	negativ	-	-	-
71	RF6	Risikofaktoren persönlich	Welche der aktuell bekannten Risikofaktoren für einen schweren Krankheitsverlauf bei einer Covid-19-Erkrankung treffen auf Sie zu?<br>**Immundefizit (z. B. HI-Virusinfektion, Antikörpermangelsyndrome, zu wenig weiße Blutkörperchen)**	/Ja<br>/Nein<br>/Keine Antwort	eigenes Item		-	-	negativ	-	-	-
72	RF7	Risikofaktoren persönlich	Welche der aktuell bekannten Risikofaktoren für einen schweren Krankheitsverlauf bei einer Covid-19-Erkrankung treffen auf Sie zu?<br>**Therapie mit Immunsuppressiva (z. B. Cortison > 5mg pro Tag, Monoklonale Antikörper, Methotrexat)**	/Ja<br>/Nein<br>/Keine Antwort	eigenes Item		-	-	negativ	-	-	-
73	RF8	Risikofaktoren persönlich	Welche der aktuell bekannten Risikofaktoren für einen schweren Krankheitsverlauf bei einer Covid-19-Erkrankung treffen auf Sie zu?<br>**Starkes Untergewicht (BMI <16, z.B. aufgrund von Magersucht / Anorexie)**	/Ja<br>/Nein<br>/Keine Antwort	eigenes Item		-	-	negativ	-	-	-
74	RF9	Risikofaktoren soziales Umfeld	Lebt in Ihrem Haushalt eine Person, auf die mindestens einer der erfragten Risikofaktoren zutrifft?	/Ja<br>/Nein<br>/Keine Antwort	eigenes Item	Risikofaktoren schwerer Verlauf COVID-19 - soziales Umfeld	-	-	negativ	-	-	-
75	RF10	Risikofaktoren soziales Umfeld	Haben Sie regelmäßig persönlichen Kontakt mit einer Ihnen nahestehenden Person (z. B. Familie, Freundeskreis, Verein), auf die mindestens einer der erfragten Risikofaktoren zutrifft?	/Ja<br>/Nein<br>/Keine Antwort	eigenes Item		-	-	negativ	-	-	-
76	DEP1	Depression	Wie oft fühlten Sie sich im Verlauf der letzten 2 Wochen durch die folgenden Beschwerden beeinträchtigt?<br>**Wenig Interesse oder Freude an Ihren Tätigkeiten**	/Überhaupt nicht<br>/An einzelnen Tagen<br>/An mehr als der Hälfte der Tage<br>/Beinahe jeden Tag	PHQ-4	PHQ-4	ja	ja	negativ	Der Patient Health Questionaire (PHQ-4) wurde entwickelt von R. Spitzer, K. Kroenke, J. Williams & B. Löwe, 2005. Deutsche Version von B. Löwe et al., Universitätsklinikum Hamburg-Eppendorf, 2006	Kroenke, K., Spitzer, R. L., Williams, J. B. W., Löwe, B. (2009). An ultra-brief screening scale for anxiety and depression: the PHQ-4 Psychosomatics, 50, 613-621.	Kroenke, K., Spitzer, R. L., Williams, J. B. W., Löwe, B. (2009). An ultra-brief screening scale for anxiety and depression: the PHQ-4 Psychosomatics, 50, 613-621.
77	ANG1	Generalisierte Angst	Wie oft fühlten Sie sich im Verlauf der letzten 2 Wochen durch die folgenden Beschwerden beeinträchtigt?<br>**Nicht in der Lage sein, Sorgen zu stoppen oder zu kontrollieren**	/Überhaupt nicht<br>/An einzelnen Tagen<br>/An mehr als der Hälfte der Tage<br>/Beinahe jeden Tag	PHQ-4		ja	ja	negativ			
78	ANG2	Generalisierte Angst	Wie oft fühlten Sie sich im Verlauf der letzten 2 Wochen durch die folgenden Beschwerden beeinträchtigt?<br>**Nervosität, Ängstlichkeit oder Anspannung**	/Überhaupt nicht<br>/An einzelnen Tagen<br>/An mehr als der Hälfte der Tage<br>/Beinahe jeden Tag	PHQ-4		ja	ja	negativ			
79	DEP2	Depression	Wie oft fühlten Sie sich im Verlauf der letzten 2 Wochen durch die folgenden Beschwerden beeinträchtigt?<br>**Niedergeschlagenheit, Schwermut und Hoffnungslosigkeit**	/Überhaupt nicht<br>/An einzelnen Tagen<br>/An mehr als der Hälfte der Tage<br>/Beinahe jeden Tag	PHQ-4		ja	ja	negativ			
80	COB1	Hilflosigkeit	Ich fühle mich der aktuellen COVID-19-Pandemie hilflos ausgeliefert.	/Stimme überhaupt nicht zu<br>/Stimme eher nicht zu<br>/Teils / teils<br>/Stimme eher zu<br>/Stimme voll und ganz zu	Aus Corona-Befragung der TU-Chemnitz	-	ja	-	negativ	https://phil-web.phil.tu-chemnitz.de/ow/ls/index.php?r=survey/index&sid=551712&lang=de	-	-
81	COB2	Unsicherheit/Sorgen	Nicht zu wissen, wie lange die aktuelle COVID-19-Pandemie anhalten wird, bereitet mir Sorgen.	/Stimme überhaupt nicht zu<br>/Stimme eher nicht zu<br>/Teils / teils<br>/Stimme eher zu<br>/Stimme voll und ganz zu	Aus Corona-Befragung der TU-Chemnitz	-	ja	-	negativ	https://phil-web.phil.tu-chemnitz.de/ow/ls/index.php?r=survey/index&sid=551712&lang=de	-	-
82	COB3	Gesundheitliche Sorgen	Die Aussicht, während der COVID-19-Pandemie an meiner Schule / Dienststelle zu arbeiten, bereitet mir Sorgen im Hinblick auf meine Gesundheit.	/Stimme überhaupt nicht zu<br>/Stimme eher nicht zu<br>/Teils / teils<br>/Stimme eher zu<br>/Stimme voll und ganz zu	eigenes Item	-	-	-	negativ	-	-	-
83	CA1	Corona assoziierte Ängste	Wie stark ist Ihre Angst davor, sich mit dem SARS-CoV-2-Virus zu infizieren?	0 - keine Angst [Freitext] 100-sehr starke Angst	Aus Befragung von Prof. Witthöft	-	nein	-	negativ	-	-	-
84	CA1a	angstbesetzte Orte	Wo ist Ihre Angst sich mit dem SARS-CoV-2-Virus zu infizieren am stärksten?<br>(Mehrfachnennung möglich)	/Wohnstätte<br>/med. Behandlungseinrichtungen<br>/Arbeitsplatz / Schule<br>/Betreuungseinrichtung<br>/Freizeit<br>/Speisestätte<br>/Übernachtungsstätten<br>/Verkehrsmittel<br>/Geschäfte<br>/Sonstiger Ort	eigenes Item	-	-	-	-	Antwortoptionen angelehnt an RKI-Publikation zu Orten der Infektion	-	-
85	CA2	Erwarteter Krankheitsverlauf	Wenn Sie an COVID-19 erkranken würden. Wie hoch schätzen Sie die Wahrscheinlichkeit ein, dass diese Erkrankung einen schweren Verlauf nimmt?	0 - extrem unwahrscheinlich [Freitext] 100 - extrem Wahrscheinlich	Aus Befragung Uni Duisburg	-	nein	-	negativ	https://ww2.unipark.de/uc/2020/ospe.php?qb	-	-
86	CA3	Angst davor Überträger zu sein	Wie stark ist Ihre Angst davor, dass Sie selbst zum Überträger des SARS-CoV-2-Virus werden könnten, d.h. andere Personen in Ihrer Umgebung mit dem Corona-Virus anstecken könnten?	0 - keine Angst [Freitext] 100-sehr starke Angst	Aus Befragung von Prof. Witthöft	-	nein	-	negativ		-	-
87	CA4	Ansteckung Angehöriger	Wie stark ist Ihre Angst davor, dass Freunde oder Angehörige sich mit dem SARS-CoV-2-Virus infizieren?	0 - keine Angst [Freitext] 100-sehr starke Angst	Aus Befragung von Prof. Witthöft	-	nein	-	negativ	https://nachrichten.idw-online.de/2020/07/23/online-umfrage-wer-fuehlt-sich-aktuell-durch-die-corona-krise-belastet/	-	-
88	CA5	Beeinträchtigung durch Angst vor Covid-19	Wie sehr beeinträchtigt Sie die Angst vor einer COVID-19-Erkrankung im Alltag?	0 - gar nicht [Freitext] 100 - sehr stark	eigenes Item	-	-	-	negativ	-	-	-

89	EE1	Burnout	Wie oft fühlen Sie sich von Ihrer Arbeit ausgebrannt?	/Nie<br>/Mind. ein paar Mal im Jahr<br>/Mind. einmal im Monat<br>/Einige Male pro Monat<br>/Einmal pro Woche<br>/Mehrmals pro Woche<br>/Täglich<br>/Keine Antwort	West et al. 2012	Burnout	ja	ja	negativ	Übersetzung aus Korczak et al. (2010) basierend auf Büssing A, Perrar KM. Die Messung von Burnout. Untersuchung einer deutschen Fassung des Maslach Burnout Inventory (MBI-D). Diagnostica 1992; 38: 328-353.	West CP, Dyrbye LN, Satele DV, Sloan JA, Shanafelt TD. Concurrent validity of single-item measures of emotional exhaustion and depersonalization in burnout assessment. J Gen Intern Med. 2012;27(11):1445-1452. doi:10.1007/s11606-012-2015-7	keine Reliabilitätsmaße vorhanden
90	EE1a	Delta zu vor COVID-19-Pandemie	Wie würden Sie diesen Aspekt im Vergleich zu vor der COVID-19-Pandemie beschreiben?	/Derzeit viel häufiger als vor der COVID-19-Pandemie<br>/Derzeit etwas häufiger als vor der COVID-19-Pandemie<br>/Etwa so häufig wie vor der COVID-19-Pandemie<br>/Derzeit etwas seltener als vor der COVID-19-Pandemie<br>/Derzeit viel seltener als vor der COVID-19-Pandemie	eigenes Item		-	-	-	-	-	-
91	DP1	Burnout	Wie oft haben Sie das Gefühl, dass Sie im Umgang mit anderen Menschen gefühlloser geworden sind, seit Sie diese Arbeit ausüben?	/Nie<br>/Mind. ein paar Mal im Jahr<br>/Mind. einmal im Monat<br>/Einige Male pro Monat<br>/Einmal pro Woche<br>/Mehrmals pro Woche<br>/Täglich<br>/Keine Antwort	West et al. 2012		ja	ja	negativ	Übersetzung aus Korczak et al. (2010) basierend auf Büssing A, Perrar KM. Die Messung von Burnout. Untersuchung einer deutschen Fassung des Maslach Burnout Inventory (MBI-D). Diagnostica 1992; 38: 328-353.	West CP, Dyrbye LN, Satele DV, Sloan JA, Shanafelt TD. Concurrent validity of single-item measures of emotional exhaustion and depersonalization in burnout assessment. J Gen Intern Med. 2012;27(11):1445-1452. doi:10.1007/s11606-012-2015-7	keine Reliabilitätsmaße vorhanden
92	DP1a	Delta zu vor COVID-19-Pandemie	Wie würden Sie diesen Aspekt im Vergleich zu vor der COVID-19-Pandemie beschreiben?	/Derzeit viel häufiger als vor der COVID-19-Pandemie<br>/Derzeit etwas häufiger als vor der COVID-19-Pandemie<br>/Etwa so häufig wie vor der COVID-19-Pandemie<br>/Derzeit etwas seltener als vor der COVID-19-Pandemie<br>/Derzeit viel seltener als vor der COVID-19-Pandemie	eigenes Item		-	-	-	-	-	-
93	PRAE	Präsentismus	An wie vielen Tagen ist es bei Ihnen in den letzten 12 Monaten vorgekommen, dass Sie gearbeitet haben, obwohl Sie sich richtig krank gefühlt haben?	/Nie<br>/1-4 Tage<br>/5-9 Tage<br>/10-14 Tage<br>/15 Tage und mehr	Institut DGB-Index Gute Arbeit (2019) - Jahresbericht 2019 - Ergebnisse der Beschäftigtenbefragung zum DGB-Index Gute Arbeit 2019.	Präsentismus	ja	-	negativ	Institut DGB-Index Gute Arbeit (2019) - Jahresbericht 2019 - Ergebnisse der Beschäftigtenbefragung zum DGB-Index Gute Arbeit 2019.<br>Zok, K. (2008b). Krank zur Arbeit: Einstellungen und Verhalten von Frauen und Männern beim Umgang mit Krankheit am Arbeitsplatz. In B. Badura, H. Schröder & C. Vetter (Hrsg.), Fehlzeiten-Report 2007. Arbeit, Geschlecht & Gesundheit (S. 121-144). Heidelberg: Springer.	-	-
94	PA1	Physical Activity	An wie vielen Tagen in der vergangenen Woche waren Sie mindestens 30 Minuten oder mehr körperlich aktiv, sodass ihre Atemfrequenz erhöht war? Dazu kann Sport, Bewegung sowie zügiges Gehen oder Radfahren zur Erholung oder zum Erreichen von Orten gehören, jedoch nicht die Hausarbeit oder körperliche Aktivitäten, die Teil Ihrer Arbeit sind.	/0 Tage<br>/1 Tag<br>/2 Tage<br>/3 Tage<br>/4 Tage<br>/5 Tage<br>/6Tage<br>/7 Tage	Milton et al., 2011	Physical Activity	ja	ja	positiv	Milton, K.; Bull, F. C.; Bauman, A. (2011): Reliability and validity testing of a single-item physical activity measure. In: British journal of sports medicine 45 (3), S. 203–208. DOI: 10.1136/bjsm.2009.068395.	Milton, K.; Bull, F. C.; Bauman, A. (2011): Reliability and validity testing of a single-item physical activity measure. In: British journal of sports medicine 45 (3), S. 203–208. DOI: 10.1136/bjsm.2009.068395.	Milton, K.; Bull, F. C.; Bauman, A. (2011): Reliability and validity testing of a single-item physical activity measure. In: British journal of sports medicine 45 (3), S. 203–208. DOI: 10.1136/bjsm.2009.068395.
95	PA1a	Delta zu vor COVID-19-Pandemie	Wie würden Sie diesen Aspekt im Vergleich zu vor der COVID-19-Pandemie beschreiben?	/Derzeit viel häufiger als vor der COVID-19-Pandemie<br>/Derzeit etwas häufiger als vor der COVID-19-Pandemie<br>/Etwa so häufig wie vor der COVID-19-Pandemie<br>/Derzeit etwas seltener als vor der COVID-19-Pandemie<br>/Derzeit viel seltener als vor der COVID-19-Pandemie	eigenes Item		-	-	-	-	-	-
96	COB4	Suchtmittelgebruach	Ich nehme seit Beginn der COVID-19-Pandemie vermehrt Suchtmittel (z.B. Alkohol, Beruhigungsmittel) zu mir.	/Stimme überhaupt nicht zu<br>/Stimme eher nicht zu<br>/Teils / teils<br>/Stimme eher zu<br>/Stimme voll und ganz zu	eigenes Item	-	-	-	negativ	-	-	-
97	COB5	Problematisches Informationsverhalten	Ich informiere mich übermäßig häufig zur aktuellen Lage der COVID-19-Pandemie.	/Stimme überhaupt nicht zu<br>/Stimme eher nicht zu<br>/Teils / teils<br>/Stimme eher zu<br>/Stimme voll und ganz zu	eigenes Item	-			negativ	-	-	-

98	RAU1	Screening Rauchen	Rauchen Sie zur Zeit?	/Ja, täglich<br>/Ja, gelegentlich<br>/Nein, nicht mehr<br>/Nein, ich habe noch nie geraucht	Aus Gesundheitsfragebogen IfL	Rauchstatus	-	-	negativ			
99	RAU1a	Delta zu vor COVID-19-Pandemie	Wie würden Sie diesen Aspekt im Vergleich zu vor der COVID-19-Pandemie beschreiben?	/Derzeit viel mehr als vor der COVID-19-Pandemie<br>/Derzeit etwas mehr als vor der COVID-19-Pandemie<br>/Etwa so wie vor der COVID-19-Pandemie<br>/Derzeit etwas weniger als vor der COVID-19-Pandemie<br>/Derzeit viel weniger als vor der COVID-19-Pandemie	eigenes Item		-	-	-	-	-	-
100	COPS	Unfähigkeit abzuschalten	Wie häufig können Sie in Ihrer Freizeit die Arbeit nicht vergessen?	/immer<br>/oft<br>/manchmal<br>/selten<br>/nie / fast nie	COPSOQ 2020	Unfähigkeit abzuschalten	ja	ja	negativ	https://www.copsoq.de/copsoq-fragebogen/	Ramkissoon (2019) - Dissecting the effect of workplace exposures on workers'rating of psychological health and safety	Bethelsen et al. (2020) - Validation of the Copenhagen Psychosocial Questionnaire Version III
101	COPSa	Delta zu vor COVID-19-Pandemie	Wie würden Sie diesen Aspekt im Vergleich zu vor der COVID-19-Pandemie beschreiben?	/Derzeit viel häufiger als vor der COVID-19-Pandemie<br>/Derzeit etwas häufiger als vor der COVID-19-Pandemie<br>/Etwa so häufig wie vor der COVID-19-Pandemie<br>/Derzeit etwas seltener als vor der COVID-19-Pandemie<br>/Derzeit viel seltener als vor der COVID-19-Pandemie	eigenes Item		-	-	-	-	-	-
102	BFI1	Big-Five-Inventory-10 Screening Persönlichkeit	Ich bin eher zurückhaltend, reserviert.	/Trifft überhaupt nicht zu<br>/Trifft eher zu<br>/Weder noch<br>/Eher zutreffend<br>/Trifft voll und ganz zu	BFI-10	Extraversion	ja	ja	negativ	Rammstedt, Beatrice & Kemper, Christoph & Klein, MC & Beierlein, Constanze & Kovaleva, Anastassiya. (2013). Eine kurze Skala zur Messung der fünf Dimensionen der Persönlichkeit: Big-Five-Inventory-10 (BFI-10) [A Short Scale for Assessing the Big Five Dimensions of Personality - 10 Item Big Five Inventory (BFI-10)]. Methoden – Daten – Analysen. 7. 233-249.	Rammstedt, Beatrice & Kemper, Christoph & Klein, MC & Beierlein, Constanze & Kovaleva, Anastassiya. (2013). Eine kurze Skala zur Messung der fünf Dimensionen der Persönlichkeit: Big-Five-Inventory-10 (BFI-10) [A Short Scale for Assessing the Big Five Dimensions of Personality - 10 Item Big Five Inventory (BFI-10)]. Methoden – Daten – Analysen. 7. 233-249.	Rammstedt, Beatrice & Kemper, Christoph & Klein, MC & Beierlein, Constanze & Kovaleva, Anastassiya. (2013). Eine kurze Skala zur Messung der fünf Dimensionen der Persönlichkeit: Big-Five-Inventory-10 (BFI-10) [A Short Scale for Assessing the Big Five Dimensions of Personality - 10 Item Big Five Inventory (BFI-10)]. Methoden – Daten – Analysen. 7. 233-249.
103	BFI2	Big-Five-Inventory-10 Screening Persönlichkeit	Ich schenke anderen leicht Vertrauen, glaube an das Gute im Menschen.	/Trifft überhaupt nicht zu<br>/Trifft eher zu<br>/Weder noch<br>/Eher zutreffend<br>/Trifft voll und ganz zu	BFI-10	Verträglichkeit	ja	ja	positiv	Rammstedt, Beatrice & Kemper, Christoph & Klein, MC & Beierlein, Constanze & Kovaleva, Anastassiya. (2013). Eine kurze Skala zur Messung der fünf Dimensionen der Persönlichkeit: Big-Five-Inventory-10 (BFI-10) [A Short Scale for Assessing the Big Five Dimensions of Personality - 10 Item Big Five Inventory (BFI-10)]. Methoden – Daten – Analysen. 7. 233-249.	Rammstedt, Beatrice & Kemper, Christoph & Klein, MC & Beierlein, Constanze & Kovaleva, Anastassiya. (2013). Eine kurze Skala zur Messung der fünf Dimensionen der Persönlichkeit: Big-Five-Inventory-10 (BFI-10) [A Short Scale for Assessing the Big Five Dimensions of Personality - 10 Item Big Five Inventory (BFI-10)]. Methoden – Daten – Analysen. 7. 233-249.	Rammstedt, Beatrice & Kemper, Christoph & Klein, MC & Beierlein, Constanze & Kovaleva, Anastassiya. (2013). Eine kurze Skala zur Messung der fünf Dimensionen der Persönlichkeit: Big-Five-Inventory-10 (BFI-10) [A Short Scale for Assessing the Big Five Dimensions of Personality - 10 Item Big Five Inventory (BFI-10)]. Methoden – Daten – Analysen. 7. 233-249.

104	BFI3	Big-Five-Inventory-10 Screening Persönlichkeit	Ich bin bequem, neige zur Faulheit.	/Trifft überhaupt nicht zu<br>/Trifft eher zu<br>/Weder noch<br>/Eher zutreffend<br>/Trifft voll und ganz zu	BFI-10	Gewissenhaftigkeit	ja	ja	negativ	Rammstedt, Beatrice & Kemper, Christoph & Klein, MC & Beierlein, Constanze & Kovaleva, Anastassiya. (2013). Eine kurze Skala zur Messung der fünf Dimensionen der Persönlichkeit: Big-Five-Inventory-10 (BFI-10) [A Short Scale for Assessing the Big Five Dimensions of Personality - 10 Item Big Five Inventory (BFI-10)]. Methoden – Daten – Analysen. 7. 233-249.	Rammstedt, Beatrice & Kemper, Christoph & Klein, MC & Beierlein, Constanze & Kovaleva, Anastassiya. (2013). Eine kurze Skala zur Messung der fünf Dimensionen der Persönlichkeit: Big-Five-Inventory-10 (BFI-10) [A Short Scale for Assessing the Big Five Dimensions of Personality - 10 Item Big Five Inventory (BFI-10)]. Methoden – Daten – Analysen. 7. 233-249.	Rammstedt, Beatrice & Kemper, Christoph & Klein, MC & Beierlein, Constanze & Kovaleva, Anastassiya. (2013). Eine kurze Skala zur Messung der fünf Dimensionen der Persönlichkeit: Big-Five-Inventory-10 (BFI-10) [A Short Scale for Assessing the Big Five Dimensions of Personality - 10 Item Big Five Inventory (BFI-10)]. Methoden – Daten – Analysen. 7. 233-249.
105	BFI4	Big-Five-Inventory-10 Screening Persönlichkeit	Ich bin entspannt, lasse mich durch Stress nicht aus der Ruhe bringen.	/Trifft überhaupt nicht zu<br>/Trifft eher zu<br>/Weder noch<br>/Eher zutreffend<br>/Trifft voll und ganz zu	BFI-10	Neurotizismus	ja	ja	positiv	Rammstedt, Beatrice & Kemper, Christoph & Klein, MC & Beierlein, Constanze & Kovaleva, Anastassiya. (2013). Eine kurze Skala zur Messung der fünf Dimensionen der Persönlichkeit: Big-Five-Inventory-10 (BFI-10) [A Short Scale for Assessing the Big Five Dimensions of Personality - 10 Item Big Five Inventory (BFI-10)]. Methoden – Daten – Analysen. 7. 233-249.	Rammstedt, Beatrice & Kemper, Christoph & Klein, MC & Beierlein, Constanze & Kovaleva, Anastassiya. (2013). Eine kurze Skala zur Messung der fünf Dimensionen der Persönlichkeit: Big-Five-Inventory-10 (BFI-10) [A Short Scale for Assessing the Big Five Dimensions of Personality - 10 Item Big Five Inventory (BFI-10)]. Methoden – Daten – Analysen. 7. 233-249.	Rammstedt, Beatrice & Kemper, Christoph & Klein, MC & Beierlein, Constanze & Kovaleva, Anastassiya. (2013). Eine kurze Skala zur Messung der fünf Dimensionen der Persönlichkeit: Big-Five-Inventory-10 (BFI-10) [A Short Scale for Assessing the Big Five Dimensions of Personality - 10 Item Big Five Inventory (BFI-10)]. Methoden – Daten – Analysen. 7. 233-249.
106	BFI5	Big-Five-Inventory-10 Screening Persönlichkeit	Ich habe nur wenig künstlerisches Interesse.	/Trifft überhaupt nicht zu<br>/Trifft eher zu<br>/Weder noch<br>/Eher zutreffend<br>/Trifft voll und ganz zu	BFI-10	Offenheit	ja	ja	negativ	Rammstedt, Beatrice & Kemper, Christoph & Klein, MC & Beierlein, Constanze & Kovaleva, Anastassiya. (2013). Eine kurze Skala zur Messung der fünf Dimensionen der Persönlichkeit: Big-Five-Inventory-10 (BFI-10) [A Short Scale for Assessing the Big Five Dimensions of Personality - 10 Item Big Five Inventory (BFI-10)]. Methoden – Daten – Analysen. 7. 233-249.	Rammstedt, Beatrice & Kemper, Christoph & Klein, MC & Beierlein, Constanze & Kovaleva, Anastassiya. (2013). Eine kurze Skala zur Messung der fünf Dimensionen der Persönlichkeit: Big-Five-Inventory-10 (BFI-10) [A Short Scale for Assessing the Big Five Dimensions of Personality - 10 Item Big Five Inventory (BFI-10)]. Methoden – Daten – Analysen. 7. 233-249.	Rammstedt, Beatrice & Kemper, Christoph & Klein, MC & Beierlein, Constanze & Kovaleva, Anastassiya. (2013). Eine kurze Skala zur Messung der fünf Dimensionen der Persönlichkeit: Big-Five-Inventory-10 (BFI-10) [A Short Scale for Assessing the Big Five Dimensions of Personality - 10 Item Big Five Inventory (BFI-10)]. Methoden – Daten – Analysen. 7. 233-249.
107	BFI6	Big-Five-Inventory-10 Screening Persönlichkeit	Ich gehe aus mir heraus, bin gesellig.	/Trifft überhaupt nicht zu<br>/Trifft eher zu<br>/Weder noch<br>/Eher zutreffend<br>/Trifft voll und ganz zu	BFI-10	Extraversion	ja	ja	positiv	Rammstedt, Beatrice & Kemper, Christoph & Klein, MC & Beierlein, Constanze & Kovaleva, Anastassiya. (2013). Eine kurze Skala zur Messung der fünf Dimensionen der Persönlichkeit: Big-Five-Inventory-10 (BFI-10) [A Short Scale for Assessing the Big Five Dimensions of Personality - 10 Item Big Five Inventory (BFI-10)]. Methoden – Daten – Analysen. 7. 233-249.	Rammstedt, Beatrice & Kemper, Christoph & Klein, MC & Beierlein, Constanze & Kovaleva, Anastassiya. (2013). Eine kurze Skala zur Messung der fünf Dimensionen der Persönlichkeit: Big-Five-Inventory-10 (BFI-10) [A Short Scale for Assessing the Big Five Dimensions of Personality - 10 Item Big Five Inventory (BFI-10)]. Methoden – Daten – Analysen. 7. 233-249.	Rammstedt, Beatrice & Kemper, Christoph & Klein, MC & Beierlein, Constanze & Kovaleva, Anastassiya. (2013). Eine kurze Skala zur Messung der fünf Dimensionen der Persönlichkeit: Big-Five-Inventory-10 (BFI-10) [A Short Scale for Assessing the Big Five Dimensions of Personality - 10 Item Big Five Inventory (BFI-10)]. Methoden – Daten – Analysen. 7. 233-249.

108	BFI7	Big-Five-Inventory-10<br>Screening Persönlichkeit	Ich neige dazu, andere zu kritisieren.	/Trifft überhaupt nicht zu<br>/Trifft eher zu<br>/Weder noch<br>/Eher zutreffend<br>/Trifft voll und ganz zu	BFI-10	Verträglichkeit	ja	ja	negativ	Rammstedt, Beatrice & Kemper, Christoph & Klein, MC & Beierlein, Constanze & Kovaleva, Anastassiya. (2013). Eine kurze Skala zur Messung der fünf Dimensionen der Persönlichkeit: Big-Five-Inventory-10 (BFI-10) [A Short Scale for Assessing the Big Five Dimensions of Personality - 10 Item Big Five Inventory (BFI-10)]. Methoden – Daten – Analysen. 7. 233-249.	Rammstedt, Beatrice & Kemper, Christoph & Klein, MC & Beierlein, Constanze & Kovaleva, Anastassiya. (2013). Eine kurze Skala zur Messung der fünf Dimensionen der Persönlichkeit: Big-Five-Inventory-10 (BFI-10) [A Short Scale for Assessing the Big Five Dimensions of Personality - 10 Item Big Five Inventory (BFI-10)]. Methoden – Daten – Analysen. 7. 233-249.	Rammstedt, Beatrice & Kemper, Christoph & Klein, MC & Beierlein, Constanze & Kovaleva, Anastassiya. (2013). Eine kurze Skala zur Messung der fünf Dimensionen der Persönlichkeit: Big-Five-Inventory-10 (BFI-10) [A Short Scale for Assessing the Big Five Dimensions of Personality - 10 Item Big Five Inventory (BFI-10)]. Methoden – Daten – Analysen. 7. 233-249.
109	BFI8	Big-Five-Inventory-10<br>Screening Persönlichkeit	Ich erledige Aufgaben gründlich.	/Trifft überhaupt nicht zu<br>/Trifft eher zu<br>/Weder noch<br>/Eher zutreffend<br>/Trifft voll und ganz zu	BFI-10	Gewissenhaftigkeit	ja	ja	positiv	Rammstedt, Beatrice & Kemper, Christoph & Klein, MC & Beierlein, Constanze & Kovaleva, Anastassiya. (2013). Eine kurze Skala zur Messung der fünf Dimensionen der Persönlichkeit: Big-Five-Inventory-10 (BFI-10) [A Short Scale for Assessing the Big Five Dimensions of Personality - 10 Item Big Five Inventory (BFI-10)]. Methoden – Daten – Analysen. 7. 233-249.	Rammstedt, Beatrice & Kemper, Christoph & Klein, MC & Beierlein, Constanze & Kovaleva, Anastassiya. (2013). Eine kurze Skala zur Messung der fünf Dimensionen der Persönlichkeit: Big-Five-Inventory-10 (BFI-10) [A Short Scale for Assessing the Big Five Dimensions of Personality - 10 Item Big Five Inventory (BFI-10)]. Methoden – Daten – Analysen. 7. 233-249.	Rammstedt, Beatrice & Kemper, Christoph & Klein, MC & Beierlein, Constanze & Kovaleva, Anastassiya. (2013). Eine kurze Skala zur Messung der fünf Dimensionen der Persönlichkeit: Big-Five-Inventory-10 (BFI-10) [A Short Scale for Assessing the Big Five Dimensions of Personality - 10 Item Big Five Inventory (BFI-10)]. Methoden – Daten – Analysen. 7. 233-249.
110	BFI9	Big-Five-Inventory-10<br>Screening Persönlichkeit	Ich werde leicht nervös und unsicher.	/Trifft überhaupt nicht zu<br>/Trifft eher zu<br>/Weder noch<br>/Eher zutreffend<br>/Trifft voll und ganz zu	BFI-10	Neurotizismus	ja	ja	negativ	Rammstedt, Beatrice & Kemper, Christoph & Klein, MC & Beierlein, Constanze & Kovaleva, Anastassiya. (2013). Eine kurze Skala zur Messung der fünf Dimensionen der Persönlichkeit: Big-Five-Inventory-10 (BFI-10) [A Short Scale for Assessing the Big Five Dimensions of Personality - 10 Item Big Five Inventory (BFI-10)]. Methoden – Daten – Analysen. 7. 233-249.	Rammstedt, Beatrice & Kemper, Christoph & Klein, MC & Beierlein, Constanze & Kovaleva, Anastassiya. (2013). Eine kurze Skala zur Messung der fünf Dimensionen der Persönlichkeit: Big-Five-Inventory-10 (BFI-10) [A Short Scale for Assessing the Big Five Dimensions of Personality - 10 Item Big Five Inventory (BFI-10)]. Methoden – Daten – Analysen. 7. 233-249.	Rammstedt, Beatrice & Kemper, Christoph & Klein, MC & Beierlein, Constanze & Kovaleva, Anastassiya. (2013). Eine kurze Skala zur Messung der fünf Dimensionen der Persönlichkeit: Big-Five-Inventory-10 (BFI-10) [A Short Scale for Assessing the Big Five Dimensions of Personality - 10 Item Big Five Inventory (BFI-10)]. Methoden – Daten – Analysen. 7. 233-249.
111	BFI10	Big-Five-Inventory-10<br>Screening Persönlichkeit	Ich habe eine aktive Vorstellungskraft, bin fantasievoll.	/Trifft überhaupt nicht zu<br>/Trifft eher zu<br>/Weder noch<br>/Eher zutreffend<br>/Trifft voll und ganz zu	BFI-10	Offenheit	ja	ja	positiv	Rammstedt, Beatrice & Kemper, Christoph & Klein, MC & Beierlein, Constanze & Kovaleva, Anastassiya. (2013). Eine kurze Skala zur Messung der fünf Dimensionen der Persönlichkeit: Big-Five-Inventory-10 (BFI-10) [A Short Scale for Assessing the Big Five Dimensions of Personality - 10 Item Big Five Inventory (BFI-10)]. Methoden – Daten – Analysen. 7. 233-249.	Rammstedt, Beatrice & Kemper, Christoph & Klein, MC & Beierlein, Constanze & Kovaleva, Anastassiya. (2013). Eine kurze Skala zur Messung der fünf Dimensionen der Persönlichkeit: Big-Five-Inventory-10 (BFI-10) [A Short Scale for Assessing the Big Five Dimensions of Personality - 10 Item Big Five Inventory (BFI-10)]. Methoden – Daten – Analysen. 7. 233-249.	Rammstedt, Beatrice & Kemper, Christoph & Klein, MC & Beierlein, Constanze & Kovaleva, Anastassiya. (2013). Eine kurze Skala zur Messung der fünf Dimensionen der Persönlichkeit: Big-Five-Inventory-10 (BFI-10) [A Short Scale for Assessing the Big Five Dimensions of Personality - 10 Item Big Five Inventory (BFI-10)]. Methoden – Daten – Analysen. 7. 233-249.

112	SOZ0	Soziale Aspekte	Seit Beginn der COVID-19-Pandemie haben in meinem Leben private Belastungen insgesamt zugenommen.	/Stimme überhaupt nicht zu<br>/Stimme eher nicht zu<br>/Teils / teils<br>/Stimme eher zu<br>/Stimme voll und ganz zu<br>/keine Antwort			-	-	negativ	-	-	-
113	SOZ1	Soziale Aspekte	In der COVID-19-Pandemie erhalte ich genügend Unterstützung (emotional, praktisch) von Personen aus meinem sozialen Umfeld.	/Stimme überhaupt nicht zu<br>/Stimme eher nicht zu<br>/Teils / teils<br>/Stimme eher zu<br>/Stimme voll und ganz zu<br>/keine Antwort			-	-	positiv	-	-	-
114	SOZ2	Soziale Aspekte	Seit Beginn der COVID-19-Pandemie ist es in meinem Haushalt vermehrt zu Konflikten gekommen.	/Stimme überhaupt nicht zu<br>/Stimme eher nicht zu<br>/Teils / teils<br>/Stimme eher zu<br>/Stimme voll und ganz zu<br>/keine Antwort			-	-	negativ	-	-	-
115	SOZ3	Soziale Aspekte	Seit Beginn der COVID-19-Pandemie ist es zu Einschränkungen in meinen Freizeitaktivitäten (z. B. Sport, Vereinsarbeit, Treffen im Freundeskreis) gekommen.	/Stimme überhaupt nicht zu<br>/Stimme eher nicht zu<br>/Teils / teils<br>/Stimme eher zu<br>/Stimme voll und ganz zu<br>/keine Antwort			-	-	negativ	-	-	-
116	SOZ4	Soziale Aspekte	Die COVID-19-Pandemie bringt mich selbst / meinen Haushalt in große wirtschaftliche Schwierigkeiten.	/Stimme überhaupt nicht zu<br>/Stimme eher nicht zu<br>/Teils / teils<br>/Stimme eher zu<br>/Stimme voll und ganz zu<br>/keine Antwort			-	-	negativ	-	-	-
117	SOZ5	Soziale Aspekte	Mit der Betreuung meiner eigenen Kinder in Kita oder Schule, während der COVID-19-Pandemie, bin ich zufrieden.	/Stimme überhaupt nicht zu<br>/Stimme eher nicht zu<br>/Teils / teils<br>/Stimme eher zu<br>/Stimme voll und ganz zu<br>/keine Antwort			-	-	positiv	-	-	-
118	LLN	Belastungen durch Einsamkeit	Ich bin häufig allein, habe wenig Kontakte	/Nein trifft nicht zu<br>/Ja trifft zu und hat mich nicht belastet<br>/Ja trifft zu und hat mich wenig belastet<br>/Ja trifft zu und hat mich mittelmäßig belastet<br>/Ja trifft zu und hat mich stark belastet<br>/Keine Antwort	GHS-Studie. Single-Item zu Einsamkeit, Beutel et al., 2017	Einsamkeit	nein	ja	negativ	Beutel, M.E., Klein, E.M., Brähler, E. et al. Loneliness in the general population: prevalence, determinants and relations to mental health. BMC Psychiatry 17, 97 (2017). https://doi.org/10.1186/s12888-017-1262-x	-	-
119	LLNa	Delta zu vor COVID-19-Pandemie	Wie würden Sie diesen Aspekt im Vergleich zu vor der COVID-19-Pandemie beschreiben?	/Derzeit viel häufiger als vor der COVID-19-Pandemie<br>/Derzeit etwas häufiger als vor der COVID-19-Pandemie<br>/Etwa so häufig wie vor der COVID-19-Pandemie<br>/Derzeit etwas seltener als vor der COVID-19-Pandemie<br>/Derzeit viel seltener als vor der COVID-19-Pandemie	eigenes Item		-	-	-	-	-	-
120	RES1	Ressourcen	Die COVID-19-Pandemie hat zu positiven Veränderungen in meinem Leben geführt.	/Stimme überhaupt nicht zu<br>/Stimme eher nicht zu<br>/Teils / teils<br>/Stimme eher zu<br>/Stimme voll und ganz zu			-	-	positiv	-	-	-
121	RES2	Ressourcen	Ich habe das Gefühl, dass ich aktiv etwas Positives für die Gesellschaft in dieser COVID-19 Pandemie tun kann (z. B. vermehrt Hilfe anbieten)	/Stimme überhaupt nicht zu<br>/Stimme eher nicht zu<br>/Teils / teils<br>/Stimme eher zu<br>/Stimme voll und ganz zu	Aus Corona-Befragung der TU-Chemnitz		nein	-	positiv	https://phil-web.phil.tu-chemnitz.de/ow/ls/index.php?r=survey/index&sid=551712&lang=de	-	-
122	RES3	Ressourcen	Ich habe das Gefühl, dass ich aktiv etwas Positives für mich in dieser COVID-19-Pandemie tun kann.	/Stimme überhaupt nicht zu<br>/Stimme eher nicht zu<br>/Teils / teils<br>/Stimme eher zu<br>/Stimme voll und ganz zu	Aus Corona-Befragung der TU-Chemnitz	-	nein	-	positiv	https://phil-web.phil.tu-chemnitz.de/ow/ls/index.php?r=survey/index&sid=551712&lang=de	-	-

5. Sammlung von Best-Practice Beispielen a) bzgl. der Umsetzung des Infektionsschutzes sowie b) des Bildungsauftrages

Table S2. Bivariate correlations, means, and standard deviations of continuous variables.

				Correlation coefficient (Spearman)																																						
				p																																						
		M	*SD*	1	2	3	4	5	6	7	8	9	10	11	12	13	14	15	16	17	18	19	20	21	22	23	24	25	26	27	28	29	30	31	32	33	34	35	36	37	38	39
1	*Physical Activity*	3.95	2.02	--																																						
2	**Cigarette smoking**	1.62	0.94	-.018**	--																																					
3	***Persons in household***	2.71	1.19	-.038**	-.063**	--																																				
4	***Minor children in household***	1.75	0.97	-.064**	-.023**	.813**	--																																			
5	Number of classes taught	4.68	3.24	.003	.008	-.102**	-.097**	--																																		
6	*Lowest grade level taught*	5.00	3.04	.001	.069**	-.043**	-.027**	.313**	--																																	
7	*Highest grade level taught*	8.24	3.75	.007	.027**	-.059**	-.039**	.526**	.790**	--																																
8	***Emotional exhaustion***	2.65	1.77	-.088**	.023**	-.029**	-.016*	.027**	-.063**	-.046**	--																															
9	***Depersonalization***	1.07	1.58	-.027**	.027**	-.008	.003	.048**	.003	.005	.344**	--																														
10	***Loneliness***	2.58	1.49	-.076**	.034**	-.220**	-.138**	.021**	-.010	-.006	.210**	.143**	--																													
11	***General anxiety***	1.29	0.45	-.068**	.026**	-.016*	-.008	.001	-.043**	-.025**	.446**	.237**	.197**	--																												
12	***Depression***	1.25	0.43	-.076**	.044**	-.035**	-.015*	.040**	.001	.010	.443**	.279**	.230**	.564**	--																											
13	***Expected course of disease***	2.32	1.16	-.066**	.069**	-.076**	-.109**	.034**	-.001	-.019**	.217**	.110**	.109**	.161**	.132**	--																										
14	**School organizational processes**	4.88	0.90	-.031**	.011	-.003	-.006	-.016**	-.085**	-.071**	.323**	.139**	.092**	.270**	.249**	.137**	--																									
15	***Amount of information***	5.22	0.97	-.017*	.014*	-.010	-.001	.060**	.006	.030**	.279**	.144**	.081**	.198**	.185**	.119**	.403**	--																								
16	**Increase in the amount of work**	5.18	0.84	-.055**	.009	.015*	.014*	.046**	.005	.011	.324**	.132**	.090**	.237**	.220**	.159**	.425**	.394**	--																							
17	*Problems implementation of the educational mission*	5.05	0.83	-.001	.033**	-.012*	-.017**	-.009	-.027**	-.038**	.171**	.097**	.082**	.141**	.157**	.073**	.280**	.243**	.227**	--																						
18	**Change expectations of guardians**	4.72	0.97	-.023*	-.008	-.027**	-.019*	.017*	-.070**	-.054**	.218**	.129**	.077**	.189**	.181**	.136**	.229**	.243**	.292**	.202**	--																					
19	*Change achievemnt learning goals*	4.77	0.89	.006	.034**	-.026**	-.030**	.027**	.017**	-.003	.136**	.099**	.080**	.115**	.132**	.079**	.182**	.173**	.183**	.490**	.230**	--																				
20	**Private burdens**	3.41	1.21	-.071**	.002	.165**	.221**	-.019**	-.015*	-.013	.253**	.152**	.179**	.244**	.220**	.095**	.200**	.149**	.166**	.129**	.100**	.102**	--																			
21	***Household conflicts***	2.43	1.25	-.061**	.016*	.309**	.345**	-.018*	-.005	.003	.173**	.187**	.117**	.169**	.161**	.025**	.089**	.090**	.083**	.063**	.044**	.063**	.483**	--																		
22	**Restrictions in leasure activities**	4.75	0.64	-.071**	.000	.008	.021**	-.005	-.018*	-.009	.052**	-.001	.121**	.063**	.060**	.001	.090**	.075**	.063**	.080**	.030**	.055**	.135**	.067**	--																	
23	***Self-rated general health***	4.08	1.15	.165**	-.057**'	.027**	.026**	-.005	.021**	.029**	-.388**'	-.203**'	-.170**'	-.330**	-.297**'	-.355**'	-.196**'	-.150**'	-.193**'	-.096**'	-.135**'	-.086**'	-.200**	-.137**	-.016*	--																
24	***Somatic complaints in the last 4 weeks***	2.36	0.97	-.118**	.049**	-.019**	-.009	-.007	-.081**	-.076**	.485**	.255**	.197**	.446**	.391**	.278**	.281**	.250**	.284**	.148**	.219**	.128**	.254**	.188**	.069**	-.497**	--															
25	***Global job satisfaction***	2.70	0.86	.043**	-.028**	.022**	.012	-.036**	.048**	.046**	-.419**	-.216**	-.168**	-.317**	-.348**	-.159**	-.340**	-.243**	-.277**	-.205**	-.190**	-.160**	-.184**	-.119**	-.044**	.294**	-.315**	--														
26	**Workload**	3.34	0.94	-.088**	.008	.004	.013	.038**	-.038**	-.011	.525**	.221**	.168**	.406**	.375**	.181**	.471**	.358**	.476**	.234**	.265**	.170**	.275**	.154**	.093**	-.305**	.417**	-.437**	--													
27	**Work-privacy conflict**	3.47	1.03	-.116**	.004	.034**	.067**	.035**	-.030**	.001	.532**	.255**	.192**	.401**	.355**	.184**	.409**	.350**	.490**	.193**	.269**	.151**	.303**	.218**	.083**	-.333**	.437**	-.402**	.651**	--												
28	Meaningfulness of work	4.08	0.81	.013	-.005	.045**	.040**	-.146**	-.113**	-.125**	-.172**	-.204**	-.103**	-.120**	-.196**	-.065**	-.039**	-.059**	-.030**	-.048**	-.057**	-.068**	-.040**	-.074**	.021**	.143**	-.109**	.293**	-.100**	-.100**	--											
29	**Time requirements**	3.54	0.93	-.085**	.001	.058**	.080**	.013	-.033**	-.008	.382**	.162**	.095**	.268**	.233**	.106**	.313**	.279**	.380**	.158**	.191**	.118**	.203**	.140**	.070**	-.219**	.308**	-.287**	.472**	.549**	-.049**	--										
30	**Predictability of work**	2.82	1.12	.021**	.010	-.003	-.025**	-.040**	.010	-.010	-.169**	-.107**	-.091**	-.118**	-.129**	-.037**	-.181**	-.153**	-.136**	-.084**	-.093**	-.048**	-.094**	-.067**	-.042**	.091**	-.138**	.248**	-.185**	-.205**	.119**	-.171**	--									
31	Information needed for work	3.31	0.86	.004	-.011	.013*	-.000	-.050**	.004	-.007	-.209**	-.141**	-.097**	-.153**	-.164**	-.077**	-.194**	-.171**	-.147**	-.115**	-.112**	-.092**	-.110**	-.091**	-.020**	.133**	-.186**	.293**	-.204**	-.209**	.163**	-.183**	.609**	--								
32	***Influence on work***	3.00	0.98	.031**	.026**	.021**	-.005	-.123**	-.026**	-.057**	-.204**	-.121**	-.108**	-.156**	-.175**	-.099**	-.118**	-.114**	-.114**	-.076**	-.095**	-.070**	-.099**	-.072**	-.019**	.127**	-.171**	.298**	-.175**	-.177**	.214**	-.112**	.265**	.271**	--							
33	**Influence on the amount of work**	2.63	0.97	.055**	.002	.027**	.015*	-.087**	.013	-.010	-.293**	-.138**	-.112**	-.206**	-.196**	-.125**	-.225**	-.207**	-.258**	-.097**	-.165**	-.083**	-.132**	-.064**	-.033**	.175**	-.228**	.288**	-.304**	-.333**	.111**	-.262**	.272**	.261**	.444**	--						
34	***Work-related emotional demands***	4.20	0.79	.017*	.016*	-.014*	-.014*	-.168**	-.182**	-.214**	.264**	.116**	.080**	.237**	.179**	.114**	.290**	.224**	.229**	.221**	.220**	.172**	.148**	.059**	.072**	-.153**	.272**	-.196**	.327**	.320**	.099**	.234**	-.087**	-.104**	-.025**	-.130**	--					
35	***Health until retirement age***	3.27	1.15	.054**	-.026**	.044**	.055**	.011	.078**	.083**	-.445**	-.254**	-.135**	-.310**	-.297**	-.260**	-.258**	-.220**	-.216**	-.130**	-.165**	-.100**	-.157**	-.093**	-.032**	.426**	-.409**	.348**	-.338**	-.356**	.173**	-.251**	.145**	.176**	.182**	.230**	-.226**	--				
36	***Helplessness***	2.91	1.09	-.065**	.036**	.011	.017*	.005	-.029**	-.023**	.328**	.180**	.221**	.349**	.355**	.179**	.252**	.171**	.174**	.170**	.151**	.143**	.276**	.181**	.118**	-.225**	.311**	-.293**	.318**	.270**	-.123**	.175**	-.145**	-.148**	-.185**	-.187**	.177**	-.248**	--			
37	***Uncertainty/worry***	3.71	1.07	-.049**	.026**	.017*	.027**	-.039**	-.050**	-.057**	.235**	.104**	.194**	.279**	.263**	.097**	.223**	.145**	.132**	.190**	.109**	.157**	.266**	.164**	.184**	-.143**	.246**	-.204**	.248**	.204**	-.048**	.136**	-.087**	-.088**	-.112**	-.117**	.178**	-.177**	.553**	--		
38	***Health concerns***	3.43	1.26	-.039**	.030**	-.053**	-.051**	.079**	.009	.015*	.313**	.167**	.143**	.249**	.220**	.394**	.235**	.194**	.216**	.098**	.176**	.075**	.177**	.061**	.059**	-.263**	.312**	-.292**	.312**	.287**	-.080**	.186**	-.184**	-.201**	-.203**	-.229**	.165**	-.317**	.358**	.251**	--	
39	***Substance Use***	1.69	1.06	-.027**	.214**	.011	.034**	.022**	.041**	.044**	.127**	.149**	.079**	.129**	.147**	.009	.059**	.062**	.035**	.049**	.034**	.042**	.106**	.145**	.039**	-.096**	.143**	-.094**	.087**	.094**	-.078**	.065**	-.054**	-.074**	-.054**	-.051**	.037**	-.100**	.130**	.122**	.045**	--

Range of observed cases: *n* = 16,096-30,706; *M* = mean, *SD* = standard deviation, **: $p \leq .01$; *: $p \leq .05$.; variables in bold: significant ($p \leq .05$) associations with physical activity; variables in italics: significant ($p \leq .05$) associations with cigarette smoking.

Table S3. Relationship to cigarette smoking of all selected categorial variables.

	Association with cigarette smoking	
	χ^2	*p*
Gender (n = 21,231)	(3) = 145.465	< .001
Age (quartiles; n = 21,305)	(9) = 358.652	< .001
Primary school (n = 19,133)	(3) = 92.320	< .001
Secondary general school (n = 19,133)	(3) = 30.073	< .001
Secondary school (n = 19,133)	(3) = 10.701	.013
Academic secondary school (n = 19,133)	(3) = 76.338	< .001
Comprehensive school (n = 19,133)	(3) = 50.120	< .001
Special needs school (n = 19,133)	(3) = 12.343	.006
Vocational school (n = 19,133)	(3) = 52.505	< .001
Other school (n = 19,133)	(3) = 11.068	.011
Professional group—teacher (n = 20,808)	(3) = 37.483	< .001
Professional group—teaching aid (n = 20,808)	(3) = 49.640	< .001
Professional group—candidate (n = 20,808)	(3) = 30.598	< .001
Being part of school management (n = 21,247)	(3) = 6.681	.083
Employment—civil servant (n = 21,275)	(3) = 64.815	< .001
Employment—employed, permanent contract (n = 21,275)	(3) = 70.605	< .001
Employment—employed, fixed-term contract (n = 21,275)	(3) = 4.926	.117
Employment—other (n = 21,275)	(3) = 1.495	.684
Work schedule (n = 21,238)	(3) = 78.752	< .001
Subjects taught—German (n = 20,540)	(3) = 8.972	.030
Subjects taught—foreign languages (n = 20,540)	(3) = 2.382	.497
Subjects taught—STEM (n = 20,540)	(3) = 93.430	< .001
Subjects taught—social sciences (n = 20,540)	(3) = 12.722	.005
Subjects taught—artistic subjects (n = 20,540)	(3) = 25.255	< .001
Subjects taught—religion, philosophy, ethics (n = 20,540)	(3) = 13.075	.004
Subjects taught—physical education (n = 20,540)	(3) = 5.095	.165
Subjects taught—other (n = 20,540)	(3) = 57.467	< .001
Federal state—Baden-Württemberg (n = 21,122)	(3) = 20.063	< .001
Federal state—Bavaria (n = 21,122)	(3) = 2.868	.412
Federal state—Berlin (n = 21,122)	(3) = 79.494	< .001
Federal state—Brandenburg (n = 21,122)	(3) = 2.966	.397
Federal state—Bremen (n = 21,122)	(3) = 17.748	< .001
Federal state—Hamburg (n = 21,122)	(3) = 35.787	< .001
Federal state—Hesse (n = 21,122)	(3) = 4.030	.258
Federal state—Mecklenburg-Western Pomerania (n = 21,122)	(3) = 7.863	.049
Federal state—Lower Saxony (n = 21,122)	(3) = 3.168	.366
Federal state—North Rhine-Westphalia (n = 21,122)	(3) = 7.495	.058
Federal state—Rhineland-Palatinate (n = 21,122)	(3) = 53.770	< .001
Federal state—Saarland (n = 21,122)	(3) = 0.399	.940
Federal state—Saxony (n = 21,122)	(3) = 7.751	.051
Federal state—Saxony-Anhalt (n = 21,122)	(3) = 2.884	.410
Federal state—Schleswig-Holstein (n = 21,122)	(3) = 2.156	.541
Federal state—Thuringia (n = 21,122)	(3) = 0.977	.807
Multiple departments (n = 21,147)	(3) = 16.503	< .001

Close student contact (n = 21,153)	(3) = 13.598	.004
Care of students (n = 21,159)	(3) = 43.198	< .001

Note: χ^2 = Pearson Chi-square value.

Table S4. Relationship to physical activity of all selected categorial variables.

	Association with physical activity	
	χ^2	*p*
Gender (n = 21,199)	(7) = 17.655	.014
Age (quartiles; n = 21,273)	(21) = 146.180	< .001
Primary school (n = 19,101)	(7) = 23.123	.002
Secondary general school (n = 19,101)	(7) = 4.623	.706
Secondary school (n = 19,101)	(7) = 11.034	.137
Academic secondary school (n = 19,101)	(7) = 17.184	.016
Comprehensive school (n = 19,101)	(7) = 4.207	.756
Special needs school (n = 19,101)	(7) = 23.235	.002
Vocational school (n = 19,101)	(7) = 6.525	.480
Other school (n = 19,101)	(7) = 8.663	.278
Professional group—teacher (n = 20,778)	(7) = 6.372	.497
Professional group—teaching aid (n = 20,778)	(7) = 7.966	.336
Professional group—candidate (n = 20,778)	(7) = 17.348	.015
School management team (n = 21,215)	(7) = 5.629	.584
Employment—civil servant (n = 21,242)	(7) = 8.533	.288
Employment—employed, permanent contract (n = 21,242)	(7) = 9.034	.250
Employment—employed, fixed-term contract (n = 21,242)	(7) = 5.012	.658
Employment —other (n = 21,242)	(7) = 9.022	.251
Work schedule (n = 21,207)	(7) =28.364	< .001
Subjects taught—German (n = 20,513)	(7) = 3.822	.800
Subjects taught—foreign languages (n = 20,513)	(7) = 5.224	.633
Subjects taught—STEM (n = 20,513)	(7) = 27.726	< .001
Subjects taught—social sciences (n = 20,513)	(7) = 20.728	.004
Subjects taught—artistic subjects (n = 20,513)	(7) = 13.753	.056
Subjects taught—religion, philosophy, ethics (n = 20,513)	(7) = 6.731	.457
Subjects taught—physical education (n = 20,513)	(7) = 168.330	< .001
Subjects taught—other (n = 20,513)	(7) = 11.872	.105
Federal state—Baden-Württemberg (n = 21,090)	(7) = 5.455	.605
Federal state—Bavaria (n = 21,090)	(7) = 6.048	.534
Federal state—Berlin (n = 21,090)	(7) = 13.275	.066
Federal state—Brandenburg (n = 21,090)	(7) = 14.383	.045
Federal state—Bremen (n = 21,090)	(7) = 12.575	.083
Federal state—Hamburg (n = 21,090)	(7) = 16.487	.021
Federal state—Hesse (n = 21,090)	(7) = 3.391	.847
Federal state—Mecklenburg-Western Pomerania (n = 21,090)	(7) = 15.385	.031
Federal state—Lower Saxony (n = 21,090)	(7) = 11.250	.128
Federal state—North Rhine-Westphalia (n = 21,090)	(7) = 10.591	.157
Federal state—Rhineland-Palatinate (n = 21,090)	(7) = 15.600	.029
Federal state—Saarland (n = 21,090)	(7) = 5.760	.568
Federal state—Saxony (n = 21,090)	(7) = 7.453	.383
Federal state—Saxony-Anhalt (n = 21,090)	(7) = 8.100	.324
Federal state—Schleswig-Holstein (n = 21,090)	(7) = 9.207	.238
Federal state—Thuringia (n = 21,090)	(7) = 5.252	.629
Multiple departments (n = 21,117)	(7) = 8.250	.311

Close student contact (n = 21,124)	(7) = 42.934	< .001
Care of students (n = 21,131)	(7) = 28.953	< .001

Note: χ^2 = Pearson Chi-square value

Table S5. Binary logistic regression analysis to predict cigarette smoking by stepwise inclusion of the six independent variable groups.

	Cigarette smoking					
	Step 1: sociodemographic variables (R^2 = 0.019***)	Step 2: work-related variables – organizational / general conditions (R^2 = 0.040***)	Step 3: work-related variables – work related impacts and attitudes (R^2 = 0.046***)	Step 4: psychological variables (R^2 = 0.049***)	Step 5: SARS-CoV-2-related variables (R^2 = 0.057***)	Step 6: health-related variables (R^2 = 0.116***)
	OR (95% CI)	OR (95% CI)	OR (95% CI)	OR (95% CI)	OR (95% CI)	OR (95% CI)
**Sociodemographic variables**						
Gender (female)	0.669 (0.585─0.764)***	0.678 (0.587─0.783)***	0.661 (0.572─0.765)***	0.653 (0.564─0.755)***	0.629 (0.543─0.729)***	0.692 (0.595─0.804)***
Persons in household	0.805 (0.764─0.849)***	0.823 (0.779─0.870)***	0.826 (0.781─0.873)***	0.825 (0.780─0.872)***	0.826 (0.781─0.873)***	0.809 (0.764─0.856)***
**Work-related variables – organizational / general conditions**						
Primary school		0.750 (0.639─0.880)***	0.737 (0.628─0.865)***	0.732 (0.623─0.860)***	0.723 (0.615─0.850)***	0.736 (0.624─0.868)***
Academic secondary school		0.761 (0.647─0.896)***	0.777 (0.660─0.915)**	0.771 (0.654─0.908)**	0.786 (0.667─0.927)**	0.757 (0.640─0.895)***
Work schedule		1.151 (1.004─1.320)*	1.182 (1.030─1.356)*	1.169 (1.018─1.341)*	1.152 (1.003─1.322)*	1.153 (1.001─1.328)*
German		1.237 (1.083─1.413)**	1.235 (1.081─1.411)**	1.241 (1.086─1.418)***	1.228 (1.075─1.404)**	1.248 (1.088─1.430)**
STEM		0.844 (0.744─0.957)**	0.840 (0.740─0.953)**	0.841 (0.740─0.954)**	0.841 (0.741─0.956)**	0.861 (0.756─0.981)**
Social science		1.214 (1.071─1.377)**	1.212 (1.068─1.375)**	1.212 (1.068─1.375)**	1.212 (1.068─1.375)**	1.212 (1.065─1.379)**
Berlin		1.871 (1.547─2.264)***	1.907 (1.576─2.308)***	1.888 (1.559─2.286)***	1.969 (1.624─2.388)***	1.990 (1.633─2.425)***
Bremen		1.722 (1.078─2.750)*	1.700 (1.063─2.720)*	1.707 (1.067─2.732)*	1.734 (1.082─2.780)*	1.602 (0.988─2.598)
Working at multiple departments		1.255 (1.042─1.511)*	1.253 (1.040─1.509)*	1.256 (1.042─1.513)*	1.259 (1.044─1.518)*	1.209 (0.998─1.464)

Situations with close student contact		1.148 (1.007–1.309)*	1.116 (0.978–1.273)	1.115 (0.977–1.273)	1.113 (0.975–1.271)	1.102 (0.962–1.262)
**Work-related variables – work related im-pacts and attitudes**						
Global job satisfaction			0.844 (0.782–0.912)***	0.891 (0.821–0.967)**	0.895 (0.824–0.972)**	0.906 (0.832–0.986)*
Influence on work			1.077 (1.010–1.150)*	1.088 (1.020–1.161)*	1.091 (1.021–1.164)**	1.102 (1.030–1.178)**
Workability			0.933 (0.882–0.988)*	0.970 (0.913–1.030)	0.979 (0.920–1.041)	1.004 (0.943–1.070)
**Psychological variables**						
Emotional Exhaustion				1.052 (1.009–1.098)*	1.043 (0.999–1.088)	1.016 (0.972–1.062)
Depression				1.171 (1.004–1.366)*	1.119 (0.957–1.308)	0.984 (0.838–1.155)
**SARS-CoV-2-related variables**						
Burden due to problems with the implementation of the educational mission					1.095 (1.011–1.186)*	1.100 (1.013–1.193)*
Uncertainty					1.119 (1.049–1.192)***	1.060 (0.992–1.131)
Health Concerns					0.908 (0.857–0.961)***	0.920 (0.868–0.975)**
Expected course of disease					1.143 (1.080–1.211)***	1.152 (1.086–1.222)***
**Health-related variables**						
Physical activity						0.945 (0.915–0.975)***
Substance use						1.586 (1.507–1.669)***

Observed cases: n = 9,414; ***: $p \leq .001$; **: $p \leq .01$; *: $p \leq .05$; Step 1: χ^2 (2) = 97.949; Step 2: χ^2 (12) = 205.543; Step 3: χ^2 (15) = 239.880; Step 4: χ^2 (17) = 254.170; Step 5: χ^2 (21) = 296.789; Step 6: χ^2 (23) = 615.506.

Table S6. Binary logistic regression analysis to predict physical inactivity by stepwise inclusion of the six independent variable groups.

	Physical inactivity					
	Step 1: sociodemographic variables (R^2 = 0.004***)	Step 2: work-related variables – organizational / general conditions (R^2 = 0.013***)	Step 3: work-related variables – work related impacts and attitudes (R^2 = 0.022***)	Step 4: psychological variables (R^2 = 0.024***)	Step 5: SARS-CoV-2-related variables (R^2 = 0.031***)	Step 6: health-related variables (R^2 = 0.039***)
	OR (95% CI)	OR (95% CI)	OR (95% CI)	OR (95% CI)	OR (95% CI)	OR (95% CI)
**Sociodemographic variables**						
Age	0.991 (0.985─0.997)***	0.990 (0.984─0.996)**	0.992 (0.986─0.998)**	0.993 (0.987─0.999)*	0.991 (0.985─0.998)**	0.989 (0.983─0.995)***
Minor children in household	1.070 (1.003─1.141)***	1.051 (0.981─1.125)	1.039 (0.970─1.113)	1.056 (0.985─1.132)	1.034 (0.961─1.113)	1.038 (0.965─1.117)
**Work-related variables – organizational / general conditions**						
Primary school		1.170 (1.025─1.336)*	1.203 (1.051─1.375)**	1.201 (1.050─1.374)**	1.203 (1.051─1.377)**	1.203 (1.051─1.377)**
Work schedule		0.867 (0.759─0.990)*	0.878 (0.768─1.003)	0.878 (0.768─1.003)	0.890 (0.778─1.017)	0.904 (0.790─1.034)
Physical education		0.648 (0.558─0.753)***	0.666 (0.573─0.773)***	0.665 (0.572─0.773)***	0.666 (0.573─0.775)***	0.678 (0.583─0.789)***
**Work-related variables – work related im-pacts and attitudes**						
Work-privacy conflict			1.137 (1.056─1.225)***	1.116 (1.035─1.203)**	1.086 (1.006─1.173)*	1.037 (0.959─1.121)
Time requirements			1.106 (1.022─1.197)*	1.104 (1.202─1.194)*	1.096 (1.012─1.186)*	1.082 (0.999─1.171)
Work-related emotional demands			0.845 (0.774─0.924)***	0.843 (0.772─0.992)***	0.829 (0.758─0.907)***	0.820 (0.750─0.897)***
**Psychological variables**						

Loneliness				1.066 (1.022─1.112)**	1.047 (1.003─1.093)*	1.038 (0.994─1.084)
**SARS-CoV-2-related variables**						
Household conflicts					1.063 (1.008─1.122)*	1.047 (0.992─1.105)
Restrictions in leisure activities					1.203 (1.092─1.326)***	1.216 (1.103─1.340)***
Expected course of disease					1.081 (1.022─1.143)**	1.036 (0.977─1.098)
**Health-related variables**						
Self-rated general health						0.837 (0.788─0.889)

Observed cases: *n* = 6,260; ***: $p \leq .001$; **: $p \leq .01$; *: $p \leq .05$; Step 1: χ^2 (2) = 16.348; Step 2: χ^2 (5) = 54.433; Step 3: χ^2 (8) = 89.504; Step 4: χ^2 (9) = 98.481; Step 5: χ^2 (12) = 125.703; Step 6: χ^2 (13) = 159.724.
